# Phase 2, open-label, noncomparative clinical trial evaluating safety and efficacy of posaconazole in pediatric patients with proven/probable invasive aspergillosis or possible invasive fungal disease

**DOI:** 10.1128/aac.01305-25

**Published:** 2026-01-27

**Authors:** Hyoung Jin Kang, Antonio C. Arrieta, Catharina Dhooge, Ágnes Kelemen, Mercedes Macías-Parra, Lourdes Aranda, Yulia V. Dinikina, Imad Kassis, Simone Cesaro, Aimee Shepherd, Arvind K. Shah, Tiffany Mackey, Hetty Waskin, Matthew G. Johnson

**Affiliations:** 1 Seoul National University College of Medicine, Seoul, Republic of Korea; 2 Seoul National University Cancer Research Institute 65492, Seoul, Republic of Korea; 3 Seoul National University Children’s Hospital, Seoul, Republic of Korea; 4 Children’s Hospital of Orange County, Orange, California, USA; 5 University of California 8788, Irvine, California, USA; 6 Ghent University Hospital, Ghent, Belgium; 7 B.-A.Z. Vármegyei Central Hospital, Miskolc, Hungary; 8 Instituto Nacional de Pediatría 37759, Mexico City, Mexico; 9 Hospital Nacional Edgardo Rebagliati Martins 279700 , Lima, Peru; 10 Almazov National Medical Research Centre 123488, Saint Petersburg, Russia; 11 Rambam Medical Center, Haifa, Israel; 12 Azienda Ospedaliera Universitaria Integrata 9286, Verona, Italy; 13 Merck & Co., Inc. 2793 , Rahway, New Jersey, USA; 14 Merck & Co., Inc. (author working under contract with Parexel International, Atlanta, GA, USA), Rahway, New Jersey, USA; University Children's Hospital Münster, Münster, Germany

**Keywords:** triazole, children, invasive fungal infections, invasive mold infections, *Aspergillus*

## Abstract

Children with invasive aspergillosis (IA) experience significant morbidity and mortality. Posaconazole is a broad-spectrum triazole antifungal agent indicated for IA treatment in adolescents and adults. A phase 2, open-label, noncomparative, multinational clinical trial in pediatric participants (2-to-<18 years old, body weight ≥10 kg) with possible, probable, or proven IA was conducted. Participants received intravenous posaconazole for ≥1 week, after which they could switch to oral posaconazole for a total treatment duration <12 weeks. Posaconazole dosing and selection of oral formulation (tablet or oral suspension [PFS]) were weight-based. The primary endpoint was safety, assessed as treatment-related adverse events (TRAE) through 14 days after treatment cessation. Global clinical response was a secondary and all-cause mortality an exploratory endpoint. PFS palatability was assessed using a 5-point scale. Thirty-one participants (proven/probable IA *n* = 9, possible invasive fungal disease *n* = 22) received ≥1 dose of posaconazole; 14 were 2 to <12 years, and 17 were 12 to <18 years old. Median treatment duration was 49 (range: 2–88) days. Seven participants (22.6%; 95% confidence interval [CI]: 9.6, 41.1) had ≥1 TRAE (grade 1 or 2, all resolved). One participant discontinued treatment due to a nonserious TRAE. Favorable global clinical response rates through weeks 6 and 12 were 67.7% (95% CI: 48.6, 83.3) and 77.4% (95% CI: 58.9, 90.4), respectively (no relapses). Day 114 all-cause mortality was 12.9%. No participants experienced problems taking PFS, and 90.0% rated PFS palatability as very good to neutral. Posaconazole was well tolerated and associated with high clinical response rates in pediatric patients with IA.

**CLINICAL TRIALS:**

This study is registered with ClinicalTrials.gov as NCT04218851.

## INTRODUCTION

Invasive mold infections caused by *Aspergillus* spp. occur predominantly in severely immunocompromised patients and contribute substantially to the morbidity and mortality seen in these populations (1–4). In the pediatric setting, the incidence of invasive aspergillosis (IA) is significant in patients with hematologic malignancies and undergoing hematopoietic stem cell transplantation (HSCT) (5–11). Children and adolescents with acute myeloid leukemia (AML), with high-risk/relapsed acute lymphoblastic leukemia (ALL), and/or undergoing allogeneic HSCT (allo-HSCT) are most heavily impacted by IA, with case fatality rates of approximately 20%–50% (5–10). Since the number of immunocompromised children at risk of IA continues to increase, safe and effective options to prevent and treat IA in pediatric patients are urgently needed. Individualized mold-active prophylaxis during high-risk periods can effectively reduce IA incidence and mortality (8, 12–16), with empirical or preemptive antifungal therapy a broadly accepted alternative (2, 17–19). Voriconazole (in patients ≥2 years old) and liposomal amphotericin B are generally recommended as first-line therapy for IA in children (4, 19–22), but these recommendations are limited by the paucity of prospective clinical data in the pediatric setting, where new therapies to treat suspected or confirmed IA remain understudied. Pediatric-specific clinical trials are needed given the high unmet medical need for new therapy options with better efficacy and/or tolerability in this population (1, 4, 23).

Posaconazole is a triazole antifungal with broad-spectrum activity against clinically important yeast and mold pathogens, including most strains of *Aspergillus* spp., and with potential advantages in terms of tolerability and drug interactions (24, 25). Newer intravenous (IV), extended-release tablet, and powder for oral suspension (PFS) formulations achieve more predictable posaconazole exposures in pediatric patients than older oral suspension formulations (26, 27). A rigorously designed and conducted randomized, controlled, phase 3 study previously assessed the efficacy and safety of posaconazole (i.e., IV and oral tablet formulations) for the treatment of IA in adults, demonstrating non-inferiority to voriconazole (28). While 13- to 17-year-old patients (weighing ≥40 kg) were eligible, only 5 participants from that age group were ultimately enrolled. Another prospective phase 1b study evaluated posaconazole (i.e., IV and PFS formulations) pharmacokinetics and safety in neutropenic patients 2–17 years old at high risk of invasive fungal infections (26). Results from that trial suggested that the safety and tolerability profiles of posaconazole in children were similar to those in adults and also enabled the selection of appropriate pediatric dose regimens. Based largely on these two trials, the approved indications for posaconazole now include the primary treatment of IA in adults (and also in adolescents in the United States), as well as the prophylaxis and salvage treatment of invasive fungal infections in adults and pediatric patients (27, 29).

Additional prospectively obtained clinical data are required to further assess the potential value of posaconazole in the pediatric setting for IA treatment. We therefore conducted a phase 2 clinical trial evaluating the safety, pharmacokinetics, and efficacy of posaconazole for the treatment of IA in the pediatric population.

## MATERIALS AND METHODS

### Study design

This study (protocol MK-5592-104) was an open-label, noncomparative, phase 2 clinical trial in pediatric participants conducted at 29 study centers in 10 countries (Belgium, Greece, Hungary, Israel, Italy, Mexico, Peru, Republic of Korea, Russia, and USA) from October 2019 to July 2024 (clinicaltrials.gov NCT04218851). The full protocol for this trial, including details on all amendments to the protocol made during the trial, is available online as a Supplemental file. The study was conducted in accordance with principles of Good Clinical Practice and was approved by the appropriate (for each study site) institutional review boards and regulatory agencies. Written informed consent, as well as age-appropriate assent, was obtained from all participants as applicable and their legally acceptable representatives.

### Participants

Participants were 2 to <18 years old, with a body weight ≥10 kg and had a diagnosis of proven IA, probable IA, or possible invasive fungal disease (IFD). Disease diagnosis and classification (see Table S1 for detailed criteria) was based on EORTC/MSG consensus definitions (30, 31), which were modified to allow severe neutropenia of any duration as an acceptable host factor. Participants with ≥1 acceptable host factor for and ≥1 clinical criterion strongly suggestive of IA, but without mycologic evidence, were considered to have possible IFD. Potential participants were excluded if they had received ≥96 h of systemic antifungal therapy for the treatment of the current fungal infection or >13 days of mold-active azole prophylaxis prior to randomization. Other important exclusion criteria were chronic (≥30 days duration) IA, relapsed/recurrent IA, or refractory IA that had not responded to prior antifungal treatment; aspergilloma or allergic bronchopulmonary aspergillosis; cystic fibrosis or pulmonary sarcoidosis; a history of serious cardiac arrhythmias; and/or artificial ventilation at randomization.

### Treatment

Posaconazole treatment duration for individual participants was based on the investigator’s clinical judgment, but treatment was planned for up to 84 days (12 weeks). Posaconazole dosing was weight-based, as summarized in Table 1. Treatment was initiated with IV posaconazole for a minimum of 1 week. At any time from day 8 (i.e., the start of week 2) to day 64 (i.e., the start of week 10), participants could be switched to oral posaconazole if clinically appropriate, but such a switch was not required. Oral posaconazole was administered either in the form of PFS, for participants with a body weight of ≤40 kg at the time of switch, or in the form of a tablet (if body weight was >40 kg). After this initial switch in the posaconazole formulation, additional transitions between oral and IV posaconazole were permitted as clinically indicated, within the maximum total (IV and oral combined) treatment duration of 12 weeks.

**TABLE 1:** Posaconazole dosing regimens and durations^*a*^

Posaconazole formulation	Dosing regimen	Time frame of dosing
IV	Day 1: 6 mg/kg BID (maximum per dose: 300 mg) Day 2 through end of IV dosing: 6 mg/kg QD (maximum per dose: 300 mg)	Starting on day 1 and through day ≥7 (up to day 63)
Oral PFS (in participants with ≤40 kg body weight)	QD based on the weight band: 90 mg (10 to <12 kg); 120 mg (12 to <17 kg); 150 mg (17 to <21 kg); 180 mg (21 to <26 kg); 210 mg (26 to <36 kg); 240 mg (36–40 kg)	Starting on day ≥8 through day ≤84
Oral tablet (in participants with >40 kg body weight)	300 mg QD	Starting on day ≥8 through day ≤84

^*a*^ BID, twice daily. IV, intravenous. PFS, powder for delayed release oral suspension. QD, once daily.

### Assessments

Key study visits included on-treatment visits at week 6 (to be conducted between days 39 and 47 as per protocol) and at week 12 (to be conducted between days 81 and 89 as per protocol). In addition, an end of treatment (EOT) visit was conducted within 3 days of the last posaconazole dose (IV or oral) in participants who discontinued treatment prior to reaching week 12 for any reason (including treatment success). Post-treatment visits were scheduled for 14 days and 28 days after the last dose, and a final study visit occurred on day 114 (±7 days). Adverse events (AEs) were monitored from the start of IV study intervention through 14 days after completion of all posaconazole therapy (IV and oral). Participant survival was assessed through day 114 after treatment initiation.

### Endpoints

The safety population comprised all participants who received ≥1 dose of posaconazole. The full analysis set (FAS) population was defined as all participants with proven/probable IA or possible IFD; ≥1 observation for the respective efficacy endpoint after ≥1 dose of posaconazole; and baseline data if required for assessment of the respective endpoint.

The primary endpoint of this trial was the proportion of participants in the safety population who experienced ≥1 treatment-related adverse event (TRAE) while receiving posaconazole treatment (IV and oral) and through 14 days after EOT. Potential drug-induced liver injury (DILI) was prespecified as an event of clinical interest and was defined as either aspartate aminotransferase (AST) or alanine aminotransferase (ALT) ≥3 × upper limit of normal (ULN) in addition to both bilirubin ≥2 × ULN and alkaline phosphatase <2 × ULN during study treatment, with these changes considered related to posaconazole by the investigator. If these conditions were met, then the study therapy could be interrupted for up to 3 days; posaconazole treatment could then only be resumed if repeat monitoring of liver function testing no longer met these criteria and the initial change was not considered treatment-related.

Secondary efficacy endpoints were global clinical response and IA relapse. The global clinical response was assessed by the investigator as complete response, partial response, stable response, or progression of IA at the week 6 and week 12 visits in the FAS population; in the event of treatment discontinuation for any reason (including treatment success), global clinical response was assessed at the EOT visit. Assessment of the clinical response was based on standard 2008 EORTC/MSG consensus criteria (30); detailed definitions of each response category are provided in Table S2. IA relapse (defined as the re-emergence of clinical, radiographic, or other relevant abnormalities indicating IA) was assessed through 28 days after EOT in participants from the FAS population who had achieved complete or partial global clinical response at EOT. An important exploratory endpoint was all-cause mortality at study day 42 and day 114, assessed in the safety population.

Palatability and acceptability of the PFS formulation were assessed in all participants who received posaconazole PFS. The assessment of palatability was done on both the first and last days of PFS treatment using a 5-point facial hedonic scale (equivalent to tasting “very good,” “good,” “neither good nor bad,” “bad,” and “very bad”) and by asking about problems when taking the PFS dose (i.e., “refusing,” “spitting out” “throwing up or spitting up,” or “gagging”). The assessment was completed by the participant (if they were 14 to <18 year old), jointly by the participant and their caregiver/health care provider (if 5 to <14 years old), or by the caregiver/health care provider (if the participant was 2 to <5 years old). Posaconazole pharmacokinetics were also an important secondary endpoint, but these data are not presented here.

### Statistical analysis

Approximately 30 participants were to be enrolled overall, with the goal to achieve at least 15 participants who transitioned to oral therapy (either PFS or tablet) for ≥7 days after the end of intravenous therapy. Of the overall enrollment target, approximately 20 participants two to <12 years old (to achieve at least 10 participants in that age cohort who transitioned to oral posaconazole) and 10 participants 12 years to <18 years old (to achieve at least five participants in that age cohort who transitioned to oral posaconazole) were to be enrolled. Both age cohorts were enrolled simultaneously. The sample size was not selected based on statistical hypothesis testing but was similar to other recent studies in the same setting (32, 33).

All analyses of safety data and baseline characteristics were done in the safety population. For the primary endpoint, i.e., the percentage of participants who experienced TRAEs during the treatment period plus the first 14 days of follow-up, the corresponding 95% confidence interval (CI) was constructed using the Clopper-Pearson method. For analysis of the secondary efficacy endpoints in the FAS population, favorable global clinical response (i.e., treatment success) was defined as an investigator assessment of either complete clinical response or partial clinical response. Unfavorable global clinical response (i.e., treatment failure) was defined as an investigator assessment of either stable response or progression of IA; any death prior to week 6 or week 12 was also considered a clinical failure at the respective timepoint(s). According to the prespecified statistical analysis plan, global clinical response assessment results reported within a window of ±2 weeks around day 42 were included in the analysis of the week 6 outcome; for participants who discontinued treatment prior to this window, their EOT assessment result was carried forward to week 6 for purposes of this analysis. Similarly, assessment results reported within a window of ±4 weeks around day 84 were included in the analysis of the week 12 outcome, and for participants who discontinued treatment prior to this window, their EOT or week 6 assessment result (whichever came later if both were available) was carried forward to week 12. For the overall proportions of participants in the FAS population with a favorable global clinical response through the week 6 and week 12 visits, the corresponding 95% CI were constructed using the Clopper-Pearson method. All other data were analyzed using descriptive statistics. Primary, secondary efficacy, and exploratory endpoints were prospectively assessed in the overall analysis population. Safety was also prospectively assessed by age cohort (i.e., 2 to <12 years old versus 12 to <18 years old); secondary efficacy endpoints were retrospectively analyzed by age cohort. All missing values were excluded from analysis, and no adjustments were planned or made for multiplicity in any of the analyses. All statistical analyses were conducted using SAS version 9.4 (SAS Institute, Cary, NC, USA).

## RESULTS

### Participants

In total, 34 participants were screened, with 3 not meeting eligibility requirements. Of the 31 who were enrolled into the trial, all met criteria for inclusion into both the safety and FAS populations. Prior systemic antifungal agents (within the protocol-permitted time limits) for antifungal prophylaxis or treatment of suspected/proven IA were administered to 22/31 (71.0%) participants, i.e., fluconazole (*n* = 11 participants), echinocandins (*n* = 9), voriconazole (*n* = 7), amphotericin B formulations (*n* = 4), and/or other mold-active azoles (*n* = 2); more than one prior systemic antifungal agent was administered to nine participants. Twenty-seven participants (87.1%) completed the trial (i.e., completed all key study visits through the final visit); the remaining four participants died prior to completion, and this was the only reason why participants were considered to have discontinued the trial prematurely (Table S3). Two participants received less than 7 days of posaconazole. Both stopped it after 2 days of treatment: one participant due to an AE and the other due to physician decision.

Baseline demographic and clinical characteristics are shown in Table 2. Most participants (74.2%) were male. Almost half (45.2%) were 2 to <12 years old, and the median age was 12.0 years (range: 2–17). Most participants were enrolled with possible IFD (*n* = 22), followed by probable IA (*n* = 7) and proven IA (*n* = 2); details on how these diagnoses were made are shown in Table 3. All the IA cases represented pulmonary IA, except one case of sinonasal infection in the proven IA subgroup, and all cases of possible IFD also exhibited well-established radiographic signs indicative of pulmonary mold infection. Baseline data collected to diagnose and classify invasive fungal infection (Table 2) showed that the study population was highly immunosuppressed: 87.1% participants had a recent history of severe neutropenia, and several were receiving T-cell immunosuppressants (12.9%) or a prolonged course of corticosteroids (9.7%). The remaining four participants (*n* = 2 with probable IA and *n* = 2 with possible IFD, all in the older age cohort) who did not have a recent history of severe neutropenia as a qualifying host factor were all allo-HSCT recipients. According to primary medical history data, all participants had an underlying condition that predisposed them to IA, predominantly hematological malignancies (61.3%), other severe hematologic conditions (19.4%), or other aggressive malignant neoplasms (16.1%); of note, 29.0% were also allo-HSCT recipients.

**TABLE 2:** Key baseline demographic and clinical characteristics (safety population)^*e*^

Characteristic	2 to <12 years old (*N* = 14)		12 to <18 years old (*N* = 17)		Total (*N* = 31)	
	*n*	%	*n*	%	*n*	%
Sex						
Female	7	(50.0)	16	(94.1)	23	(74.2)
Male	7	(50.0)	1	(5.9)	8	(25.8)
Age						
Mean (SD)	6.9 years (2.7)		14.5 years (1.7)		11.1 years (4.4)	
Median (range)	8.0 years (2 to 11)		14.0 years (12 to 17)		12.0 years (2 to 17)	
Weight						
Mean (SD)	27.6 kg (12.7)		48.2 kg (8.6)		38.9 kg (14.8)	
Median (range)	25.2 kg (12.3 to 51.6)		48.9 kg (35.4 to 65.5)		43.5 kg (12.3 to 65.5)	
Race						
Asian	3	(21.4)	5	(29.4)	8	(25.8)
Multiple	2	(14.3)	4	(23.5)	6	(19.4)
White	9	(64.3)	8	(47.1)	17	(54.8)
Ethnicity						
Hispanic or Latino	2	(14.3)	5	(29.4)	7	(22.6)
Not Hispanic or Latino	12	(85.7)	12	(70.6)	24	(77.4)
Disease classification						
Proven IA	1	(7.1)	1	(5.9)	2	(6.5)
Probable IA	1	(7.1)	6	(35.3)	7	(22.6)
Possible IFD	12	(85.7)	10	(58.8)	22	(71.0)
Neutrophil count						
Mean mature neutrophils	1.137 × 10^9^/L		1.162 × 10^9^/L		1.151 × 10^9^/L	
Host factors^*a*^						
Recent severe neutropenia^*b*^	14	(100.0)	13	(76.5)	27	(87.1)
Allogeneic HSCT	2	(14.3)	7	(41.2)	9	(29.0)
T-cell immunosuppressants	1	(7.1)	3	(17.6)	4	(12.9)
Prolonged corticosteroid use	1	(7.1)	2	(11.8)	3	(9.7)
Inherited severe immunodeficiency	0	(0.0)	1^*c*^	(5.9)	1^*c*^	(3.2)
Underlying conditions^*d*^						
Hematologic malignancy	9	(64.3)	10	(58.8)	19	(61.3)
ALL	5	(35.7)	7	(41.2)	12	(38.7)
AML	3	(21.4)	3	(17.6)	6	(19.4)
Precursor B-lymphoblastic lymphoma	1	(7.1)	0	(0.0)	1	(3.2)
Non-hematologic malignancy	2	(14.3)	3	(17.6)	5	(16.1)
Medulloblastoma	1	(7.1)	2	(11.8)	3	(9.7)
Osteosarcoma	1	(7.1)	1	(5.9)	2	(6.5)
Non-malignant hematologic conditions	3	(21.4)	3	(17.6)	6	(19.4)
Aplastic anemia	1	(7.1)	3	(17.6)	4	(12.9)
Thalassemia^*c*^	1	(7.1)	0	(0.0)	1	(3.2)
Immune thrombocytopenia	1	(7.1)	0	(0.0)	1	(3.2)
Inherited immunodeficiency^*c*^	0	(0.0)	1	(5.9)	1	(3.2)
Hyper IgE syndrome^*c*^	0	(0.0)	1	(5.9)	1	(3.2)

^*a*^ Participants could have had more than one host factor. Reported prospectively by the investigator as part of IA diagnosis and classification to determine participant eligibility at study entry.

^*b*^ Defined as recent (i.e., within 30 days prior to screening) history of severe neutropenia (<0.5×10^9^ neutrophils/L) of any duration temporally related to the onset of fungal disease.

^*c*^ This participant was also an allo-HSCT recipient.

^*d*^ Retrospectively determined from primary medical history data.

^*e*^ ALL, acute lymphocytic leukemia. AML, acute myeloid leukemia. HSCT, hematopoietic stem cell transplant. IA, invasive aspergillosis. IFD, invasive fungal disease. n, number of participants in the analysis population who met the criterion of the corresponding row title. SD, standard deviation.

**TABLE 3:** Participants’ qualifying host factors, clinical criteria, and mycologic criteria (safety population)^*f*^^,*g*^

Characteristic	Proven IA (*N* = 2)	Probable IA (*N* = 7)	Possible IFD (*N* = 22)	Total (*N* = 31)
Host factors				
Recent history of severe neutropenia^*a*^	2	5	20	27 (87.1%)
Receipt of an allo-HSCT	0	4	5	9 (29.0%)
Treatment with T-cell immunosuppressants	0	2	2	4 (12.9%)
Prolonged use of corticosteroids	0	2	1	3 (9.7%)
Inherited severe immunodeficiency	0	0	1	1 (3.2%)
Clinical criteria				
Radiographic evidence suggestive of pulmonary mold infection	1	7	22	30 (96.8%)
Sinonasal infection	1	0	0	1 (3.2%)
Mycologic criteria				
Culture of sterile material	1^*b*^	0	0	1 (3.2%)
Microscopy of sterile material	1^*c*^	0	0	1 (3.2%)
Direct tests	2^*b*^^,*c*^	1^*d*^	0	3 (9.7%)
Indirect tests^*e*^	0	6	0	6 (19.4%)

^*a*^ Defined as recent (i.e., within 30 days prior to screening) history of severe neutropenia (<0.5 × 10^9^ neutrophils/L) of any duration temporally related to the onset of fungal disease.

^*b*^ Participant with ethmoid sinus mucus with many fungal hyphae on microscopy and culture grew *Aspergillus flavus*; nasal tissue culture grew *A. flavus*.

^*c*^ Participant with lung tissue histopathology with extensive necrotic tissue with abundant fungal organisms, mainly hyphae; biopsy results were positive for *Aspergillus fumigatus*.

^*d*^ Participant with bronchoalveolar lavage (BAL) fluid culture that grew *Aspergillus niger*.

^*e*^ Qualifying tests were galactomannan assay of serum, BAL fluid, and/or CSF or *Aspergillus* PCR of serum and/or BAL fluid. See Table S1 for details.

^*f*^ Participants could have had more than 1 qualifying host factor, clinical criterion, or mycologic criterion to be eligible for the study.

^*g*^ IA, invasive aspergillosis. IFD, invasive fungal disease. HSCT, hematopoietic stem cell transplant.

Overall, the median (range) total treatment duration was 49 (2–88) days. The median (range) duration of IV posaconazole was 8 (2–78) days, with nine participants receiving only IV treatment. Twelve participants were switched to posaconazole tablets, with a median (range) oral treatment duration of 67.5 (6–80) days, and 10 were switched to posaconazole PFS, with a median (range) duration of 48 (7–76) days. A total of 10 participants (32.3%) completed the maximum 12 weeks of treatment, 3 (9.7%) died while receiving treatment, and 2 (6.5%) discontinued posaconazole due to AEs. The remaining 16 (51.6%) participants discontinued treatment prior to week 12 due to investigator decision. The high rate of discontinuation from the study treatment prior to week 12 was expected because the actual treatment duration for each participant was based on the investigator’s clinical judgment. Of the 16 participants who discontinued treatment prior to week 12 due to physician decision, 14 were considered to have had a complete or partial response (11 of these had possible IFD, 2 had probable IA, and 1 proven IA), 1 had a stable response (with a diagnosis of possible IFD), and 1 had progression of IA (also with a diagnosis of possible IFD). Of the 8 participants who discontinued treatment prior to week 6 due to physician decision, 4 (all with possible IFD) were considered to have had a complete response, 2 (both with possible IFD) had a partial response, and 1 each (also both with possible IFD) had a stable response or disease progression.

### Safety

Seven participants (22.6%; 95% CI: 9.6, 41.1) had ≥1 TRAE (Table 4). All of these TRAEs were grade 1 or 2, nonserious, and resolved: abnormal liver enzymes (*n* = 3), gastrointestinal disorders (*n* = 2), feeling hot (*n* = 1), and infusion-related reaction (*n* = 1). Only one participant discontinued treatment due to a TRAE (i.e., increased liver function tests, severity grade 2). This participant had elevated ALT and bilirubin levels at baseline and on day 2 had liver function test results that met criteria for potential DILI. The participant was asymptomatic, the event was not considered serious, and no alternative etiology for the event was provided. Posaconazole was permanently discontinued right away (i.e., on day 2), and the investigator considered the event resolved on day 3. Follow-up testing was unremarkable, and the participant completed the trial.

**TABLE 4:** AE summary (safety population)^*e*^^,^^*f*^

Characteristic	2 to <12 years old (*N* = 14)		12 to <18 years old (*N* = 17)		Total (*N* = 31)	
	*n*	(%)	*n*	(%)	*n*	(%)
All-causality AEs	12	(85.7%)	15	(88.2%)	27	(87.1%)
Serious^*a*^	4	(28.6%)	8	(47.1%)	12	(38.7%)
Deaths^*a*^	1^*b*^	(7.1%)	3^*c*^	(17.6%)	4	(12.9%)
Leading to treatment discontinuation	1	(7.1%)	1	(5.9%)	2	(6.5%)
**Treatment-related AEs** ^*d*^	**2**	**(14.3%)**	**5**	**(29.4%)**	**7**	**(22.6%)**
Serious	0	(0.0%)	0	(0.0%)	0	(0.0%)
Deaths	0	(0.0%)	0	(0.0%)	0	(0.0%)
Leading to treatment discontinuation	0	(0.0%)	1	(5.9%)	1	(3.2%)

^*a*^ None of these were considered related to posaconazole.

^*b*^ Cause of death: extramedullary leukemic infiltration (participant discontinued posaconazole after 12 days of therapy due to extramedullary leukemic infiltration that was not treatment-related and achieved a partial response; died on study day 24 due to the same AE).

^*c*^ Causes of death: pulmonary hemorrhage (died on day 7 of therapy), hematemesis (died on day 11 of therapy), and thrombocytopenia (died on day 32 of therapy).

^*d*^ The proportion of participants with treatment-related AEs (highlighted in bold) was the primary endpoint of the trial.

^*e*^ AEs were assessed through 14 days after the last dose. All-causality AEs were treatment-emergent AEs regardless of whether they were determined by the investigator to be related to posaconazole. Treatment-related AEs were those that the investigator determined to be related to posaconazole.

^*f*^ AE, adverse event. *n*, number of participants with the indicated AE.

All-causality AEs (i.e., irrespective of whether the investigator deemed them related to posaconazole treatment or not) occurred in 87.1% of all participants (Table 4). The most frequent AEs (i.e., reported in ≥4 [15%] of participants) were as follows (Table S4): vomiting (32.3%), pyrexia (29.0%), hypertension (25.8%), abdominal pain (19.4%), febrile neutropenia (16.1%), diarrhea (16.1%), nausea (16.1%), and decreased appetite (16.1%). None of the hypertension AEs were considered treatment-related by the investigator; posaconazole therapy was continued without interruption during these AEs, and all the events resolved. Three of the eight participants with hypertension also had AEs of hypokalemia co-reported during the treatment period, i.e., 5 days after hypertension for 1 participant and before hypertension for 2 participants (4 days prior and 12 days prior). None of the cases of hypokalemia were considered treatment-related by the investigator, and posaconazole therapy was continued without interruption; all 3 cases were nonserious, and 2 of them resolved while the participant was still receiving study intervention.

Serious AEs, none of which were treatment-related, were reported for 38.7% and deaths due to AEs for 12.9% of participants (Table 3). The most common serious AEs (Table S5) were febrile neutropenia and sepsis, reported in 2 (6.5%) participants each. The deaths were reported as having been due to extramedullary leukemic infiltration (the participant discontinued posaconazole after 12 days of therapy, with partial response, due to non-treatment-related extramedullary leukemic infiltration and subsequently died from this same AE on day 24) in the 2 to <12 years old cohort and pulmonary hemorrhage (died on day 7 of therapy), hematemesis (died on day 11 of therapy), and thrombocytopenia (died on day 32 of therapy) in the 12 to <18 years old cohort. All these deaths occurred in trial participants who had possible IFD (with pulmonary involvement) and AML/ALL as the underlying condition.

### Efficacy

**Fig 1:**
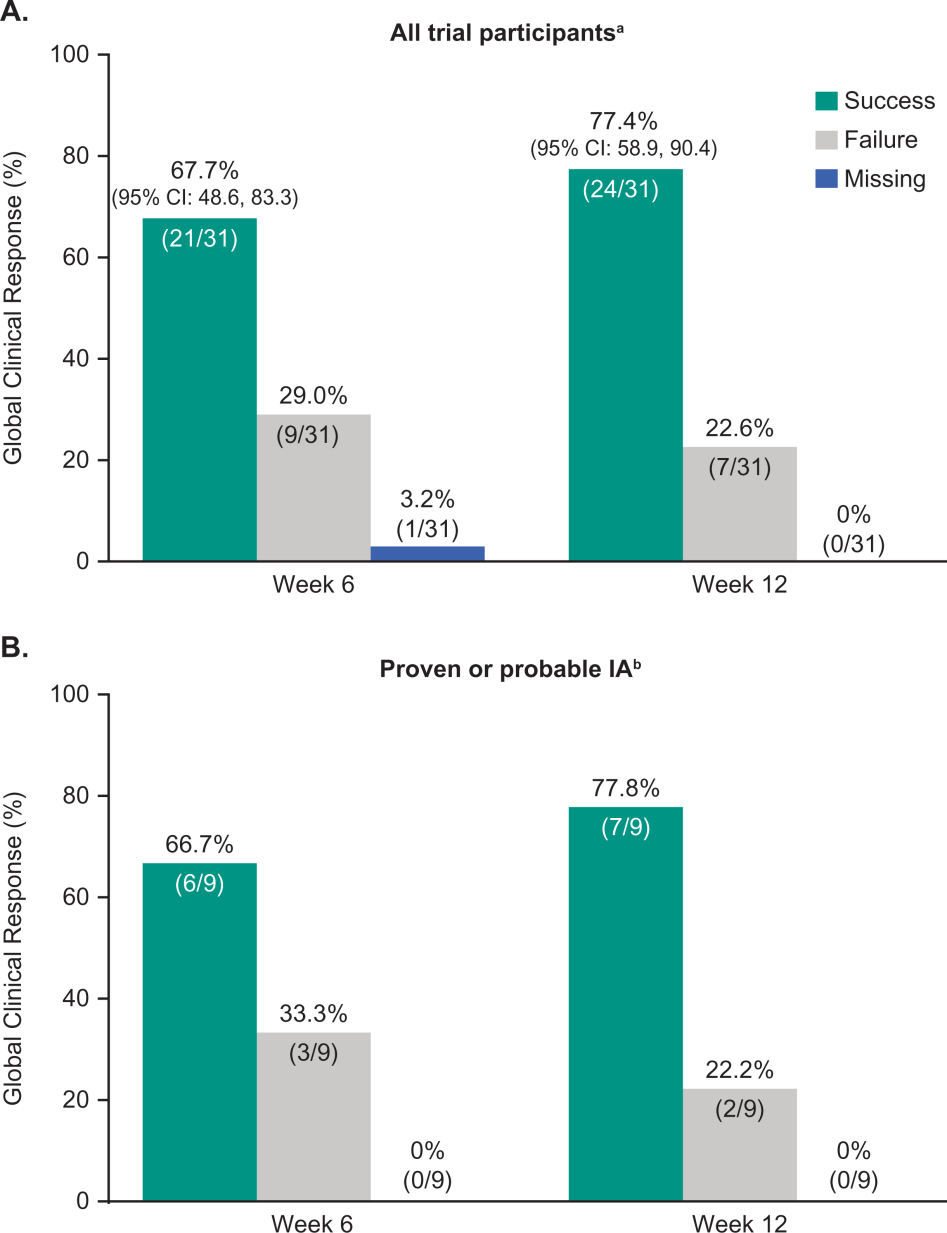
Global clinical response in the FAS population for (**A**) all trial participants and (**B**) participants with proven or probable IA. Analyzed in all participants with ≥1 observation for this endpoint after ≥1 posaconazole dose, with 95% CIs based on the Clopper-Pearson method for 2-sided exact 95% CI on a binomial proportion. Global clinical response assessment results reported within a window of ±2 weeks around day 42 were included in the analysis of the week 6 outcome; participants who discontinued treatment prior to this window had their EOT assessment result carried forward to week 6. Global clinical response assessment results reported within a window of ±4 weeks around day 84 were included in the analysis of the week 12 outcome; participants who discontinued treatment prior to this window had their EOT or week 6 assessment result (whichever came later if both were available) carried forward to week 12. IA, invasive aspergillosis. ^a^Secondary endpoint that was prospectively defined in the statistical analysis plan. ^b^*Post hoc* subgroup analysis of the secondary endpoint.

Global clinical success was achieved in 67.7% (95% CI: 48.6, 83.3) of participants in the FAS population through week 6 and 77.4% (95% CI: 58.9, 90.4) through week 12 (Fig. 1A). At week 6 and week 12, 9/21 (42.9%) and 15/24 (62.5%) of all clinical successes, respectively, were complete responses. Of the global clinical failures, 5/9 (55.6%) at week 6 and 5/7 (71.4%) at week 12 were due to disease progression or death, while conversely, the proportion of stable responses decreased from 4/9 (44.4%) to 2/7 (28.6%) (Table 5). In the subgroup of participants with either proven or probable IA, global clinical success was achieved in 6/9 participants (66.7%) at week 6 and 7/9 (77.8%) at week 12 (Fig. 1B); the respective response rates in proven IA were 1/2 (50.0%) and 2/2 (100.0%) and in probable IA 5/7 (71.4%) at both time points. In the possible IFD subgroup, clinical success was achieved in 15/22 participants (68.2%) at weeks 6 and 12.

**TABLE 5:** Summary of global clinical response (FAS^*a*^ population)

Characteristic	2 to <12 years old (*N* = 14)		12 to <18 years old (*N* = 17)		Total (*N* = 31)	
	*n*	(%)	*n*	(%)	*n*	(%)
Through week 6						
Success, complete response	6	(42.9%)	3	(17.6%)	9	(29.0%)
Success, partial response	3	(21.4%)	9	(52.9%)	12	(38.7%)
Failure, stable response	2	(14.3%)	2	(11.8%)	4	(12.9%)
Failure, progression of fungal disease	1	(7.1%)	0	(0.0%)	1	(3.2%)
Failure, death during the evaluation period	1	(7.1%)	3	(17.6%)	4	(12.9%)
Missing	1	(7.1%)	0	(0.0%)	1	(3.2%)
Through week 12						
Success, complete response	8	(57.1%)	7	(41.2%)	15	(48.4%)
Success, partial response	3	(21.4%)	6	(35.3%)	9	(29.0%)
Failure, stable response	1	(7.1%)	1	(5.9%)	2	(6.5%)
Failure, progression of fungal disease	1	(7.1%)	0	(0.0%)	1	(3.2%)
Failure, death during the evaluation period	1	(7.1%)	3	(17.6%)	4	(12.9%)
Missing	0	(0.0%)	0	(0.0%)	0	(0.0%)

^*a*^ FAS, full analysis set.

No IA relapses occurred during this trial. One participant with global clinical success died at 11 days post-treatment due to worsening of their underlying condition (i.e., ALL with hepatic extramedullary involvement), and thus relapse of IA could not be assessed in this participant. Day 42 all-cause mortality in the full safety population was 12.9% (4/31 participants), and no additional participants died through day 114. The mortality rate in 2 to <12-year-old participants was 7.1% (1/14), versus 17.6% (3/17) in 12 to <18-year-old participants. Mortality attributed specifically to IA was not assessed, but for one of these participants (i.e., in the younger age cohort), the cause of death was specifically reported as having been due to worsening of the underlying condition (i.e., progression of AML).

### Palatability

The palatability and acceptability of the posaconazole PFS formulation was assessed in all 10 participants who were switched to PFS treatment. No participant reported or was reported to have experienced any problems taking PFS (Table S6). For most participants (90.0%), PFS was reported as tasting “very good,” “good,” or “neither good nor bad” on both the first and the last day of dosing.

## DISCUSSION

This open-label, noncomparative, phase 2 clinical study evaluated the safety and efficacy of posaconazole for treatment of IA in children. The participants enrolled in our trial were at high risk of developing IA based on their baseline characteristics (30, 31, 34–36). All had underlying conditions that predisposed them to risk of IA (predominantly hematological malignancies), and almost 90% had a recent history of severe neutropenia temporally related to the onset of fungal disease, while all the remainder were allo-HSCT recipients. Posaconazole was generally well tolerated. While approximately half of participants discontinued posaconazole before week 12 due to physician decision, this result is not surprising since the treatment duration was based on the investigator’s clinical judgment. The majority (i.e., 14 of these 16 participants) had apparent treatment success so that further antifungal treatment was no longer needed. Of the 31 participants, about 20% experienced TRAEs, none of which were serious and only one (i.e., abnormal liver function tests) that resulted in (permanent) posaconazole discontinuation. The incidence of AEs, serious AEs, and TRAEs was generally comparable between the 2 to <12 and the 12 to <18-year-old age groups. The overall AE profile was as expected, and no new safety issues with posaconazole were identified. Global clinical success was achieved in 68% of all participants through week 6 and in 77% through week 12; these success rates were almost identical in the subgroup with proven/probable IA (i.e., 67% and 78%, respectively). Treatment outcomes were also generally comparable between both age groups. Day 42 all-cause mortality was 13%, with no additional deaths occurring after that time point; none of the deaths were related to posaconazole. Mortality attributable to IA was not assessed, but in three of the four deaths (i.e., from pulmonary hemorrhage, thrombocytopenia, and hematemesis in participants with ALL or AML), a potential contribution from the fungal infection cannot be excluded. Almost all participants who received the PFS formulation of posaconazole reported it to be palatable, and none experienced any problems taking PFS.

In the clinical development of antimicrobial treatments, efficacy can be extrapolated from adults to children if the infection under study does not substantially differ between age groups and if pharmacokinetic data show that similar drug exposures to adults can be attained through the pediatric dose regimen evaluated (9, 33, 37, 38). Safety, however, cannot simply be extrapolated, so this aspect is often the focus of smaller pediatric studies—safety was therefore also the primary endpoint in our trial. Importantly, the observed AE profile matched the known safety profile of posaconazole in both pediatric and adult populations (13, 26–29, 39–41). Similar to other clinical trials in this setting (28, 32, 33), most of our trial participants experienced one or more all-causality AEs, which is not surprising given the complexity of the enrolled study population. Both IA itself and the serious underlying conditions putting these patients at risk of developing IA are associated with a high incidence of collateral medical events, which in the context of a clinical trial would be reported as AEs. The specific all-causality AEs that were reported, and the relative frequency of those AEs, also aligned with expectations about the study population. Similarly, the specific TRAEs with posaconazole, i.e., elevated liver enzymes, gastrointestinal disorders, and infusion-related reactions, are among the most common posaconazole-associated AEs in children and adults.

The overall proportion of patients with TRAEs was 23%, largely in line with prior posaconazole clinical trials in children and adults as well as pediatric trials assessing voriconazole and isavuconazole. In the phase 3 trial comparing posaconazole with voriconazole for IA treatment (which randomized only 3 participants <18 years old to posaconazole), 30% of participants experienced AEs deemed related to posaconazole treatment, with 6% experiencing serious TRAEs and TRAEs that led to posaconazole discontinuation; the corresponding proportions were all greater with voriconazole. All-cause mortality was 15% (28). An open-label pediatric trial assessing voriconazole as treatment for IA reported TRAEs in 52% and serious TRAEs in 7% of participants; as expected for voriconazole, most of these were hepato-biliary, visual, and skin AEs (32). In another similar open-label trial, 29% of pediatric patients treated with isavuconazole had TRAEs (3% considered serious and 7% leading to isavuconazole discontinuation) (33). All-cause mortality in these pediatric phase 2 studies was 7% with isavuconazole and 16% with voriconazole (28, 32, 33).

Of note, posaconazole can inhibit the activities of enzymes 11β-hydroxylase (involved in aldosterone synthesis) and 11β-hydroxysteroid dehydrogenase type 2 (involved in cortisol inactivation), leading to an increase in mineralocorticoid activity independent of aldosterone. Posaconazole levels can therefore be associated with the development of pseudohyperaldosteronism, characterized by a combination of high blood pressure, low serum potassium levels, and suppressed renin and aldosterone (42–44). About one-quarter of our participants experienced hypertension (none treatment-related), of whom less than 40% also had hypokalemia (also none treatment-related). Since renin and aldosterone levels in those latter participants are not available, we cannot completely exclude potential pseudohyperaldosteronism; importantly, however, none of the affected participants had to interrupt or discontinue posaconazole therapy due to these AEs.

EORTC/MSG consensus definitions for invasive fungal infections in clinical research include all possible invasive infections (regardless of the causative pathogen) caused by any fungal pathogen under the category of “possible IFD” (31). Galactomannan or PCR testing of BAL fluid or PCR testing of tissue samples is accurate, but may often be too invasive for younger patients. Serum galactomannan assays, on the other hand, are easy to conduct but can be imprecise and are limited by diagnostic thresholds with difficult-to-interpret real-world clinical significance (35, 45–48). Serum galactomannan testing was done at baseline (for diagnostic purposes) and at the EOT visit (for assessing the treatment response); none of the participants with possible IFD had serum galactomannan above diagnostic cutoffs. Other than serum galactomannan, negative mycologic test results were not systematically recorded, nor were test results conclusively ruling out non-*Aspergillus* pathogens. IFDs caused by non-*Aspergillus* molds are more likely in children who received mold-active antifungals prior to disease onset (34, 49, 50). Since mold-active prophylaxis of ≥2 weeks was an exclusion criterion, the majority of possible IFD (i.e., occurring in vulnerable patients with host factors and radiographic evidence of invasive mold infection) in our trial would have been caused by *Aspergillus* spp. In any case, it is highly likely that in real-world patients similar to our trial participants with possible IFD, clinicians would rapidly initiate preemptive therapy with a broad-spectrum antifungal agent covering the possibility of both aspergillosis and mucormycosis (e.g., liposomal amphotericin B, isavuconazole, or posaconazole (21, 22, 51–56).

Efficacy was assessed using clinically relevant outcomes standard for antifungal trials: global clinical response, relapse, and all-cause mortality. We utilized the EORTC/MSG definitions (initially the 2008, then the 2020 criteria) for global response (30) for consistency with the adult posaconazole treatment study (28) and other recent IA treatment studies conducted in children (32, 33). Formal comparisons of our results with historical data or previously published clinical trials are not appropriate, in large part because these were all relatively small trials in which minor absolute differences in events (e.g., number of participants experiencing treatment failure) can result in noticeable differences in event rates (e.g., global response rates and all-cause mortality).

Our trial employed a rigorous study design that met all regulatory guidelines for pediatric clinical development of antifungal agents. Another strength was that the enrolled study population compared well to real-world patients likely to be treated with systemic mold-active therapies. On the other hand, this was an open-label, noncomparative phase 2 trial, considered to provide a lower quality of evidence than double-blind, randomized, controlled phase 3 clinical trials. Of note, the latter (comparing different antifungal agents) are not feasible in children given the fewer pediatric patients at risk for IA compared with at-risk adults. Due to the relatively small sample size of our trial, the results could be more prone to variability in the data, somewhat reducing our ability to accurately measure the true treatment effect of posaconazole. Clinical success rates, especially treatment outcomes by subgroup, thus need to be interpreted with caution. As discussed above, the high percentage of possible IFD cases was another limitation of this study. Since there were so few participants who had *Aspergillus* isolated from qualified samples and because the susceptibility of those isolates to posaconazole was not systematically assessed, the potential impact of azole resistance on clinical outcomes is unknown. While the vast majority of strains of *Aspergillus* spp. are known to be posaconazole-susceptible (25, 57–61), it is possible that undetected (baseline or treatment-emergent) drug resistance could have contributed to clinical failures. Treatment outcomes with systemic antifungal agents can also be negatively affected by subtherapeutic drug levels, one of the reasons that therapeutic drug monitoring (TDM) is sometimes recommended with certain antifungal therapies (4, 19, 53, 62–64). Posaconazole TDM is therefore used at some centers to guide dose adjustments but is not included in any regulatory label for posaconazole worldwide and was not employed in this study. It should also be noted that the newer posaconazole formulations used in our trial exhibit more predictable pharmacokinetics and less variability in drug exposures than the older oral formulations (26, 39).

In conclusion, our study provides prospective data on the safety and utility of posaconazole to treat IA in pediatric populations. Posaconazole exhibited an AE profile comparable to that observed in other pediatric and adult trials with this particular agent and reported for other mold-active triazoles in pediatrics. Posaconazole also yielded high clinical success rates. Based on the totality of the evidence (i.e., a good tolerability profile, no known safety issues in children, and efficacy in treating IA consistent with adults), posaconazole could be a treatment option for IA in children and adolescents.

## Data Availability

The data sharing policy, including procedures and restrictions, of Merck Sharp & Dohme LLC, a subsidiary of Merck & Co., Inc., Rahway, NJ, USA (MSD), is available at https://externaldatasharing-msd.com/.

## SUPPLEMENTAL MATERIAL

Supplemental material aac.01305-25-s0001.docx Tables S1 to S6.

Supplemental file aac.01305-25-s0002.pdf MK-5592-104 study protocol.

ASM does not own the copyrights to Supplemental Material that may be linked to, or accessed through, an article. The authors have granted ASM a non-exclusive, world-wide license to publish the Supplemental Material files. Please contact the corresponding author directly for reuse.
