# Supplementary material for: Phase 2, open-label, noncomparative clinical trial evaluating safety and efficacy of posaconazole in pediatric patients with proven/probable invasive aspergillosis or possible invasive fungal disease
Source: Antimicrob Agents Chemother. 2026 Jan 27;70(3):e01305-25. doi: 10.1128/aac.01305-25 (PMC12959131; doi:10.1128/aac.01305-25)
Supplement: Supplemental material — Tables S1 to S6. [file aac.01305-25-s0001.docx]

**Online supplementary material to:**

**Phase 2, open-label, non-comparative clinical trial evaluating safety and efficacy of posaconazole in pediatric patients with proven/probable invasive aspergillosis or possible invasive fungal disease**

Table of Contents

[Table S1: Disease diagnosis and classification criteria 2](#_Toc214893608)

[Table S2: Definitions for global clinical response 6](#_Toc214893609)

[Table S3: Disposition of all enrolled participants 7](#_Toc214893610)

[Table S4: Full listing of adverse events (regardless of assumed relationship to study intervention) from first dose of posaconazole up to and including 14 days after the last dose in the safety population 8](#_Toc214893611)

[Table S5: Full listing of serious adverse events (regardless of assumed relationship to study intervention) from first dose of posaconazole up to and including 14 days after the last dose in the safety population 16](#_Toc214893612)

[Table S6: Summary of palatability and acceptance of posaconazole powder for oral suspension in all participants who were treated with this formulation 18](#_Toc214893613)

# Table S1: Disease diagnosis and classification criteria

| **PROVEN invasive aspergillosis** |
| --- |
| **Tissue nucleic acid diagnosis:** Amplification of fungal DNA by PCR combined  with DNA sequencing when molds are seen in formalin-fixed paraffin-embedded tissue  OR  **Microscopic analysis of sterile material:** Tissue histopathologic, cytopathologic, or  direct microscopic examination of a needle aspiration or biopsy specimen showing  hyphal forms with evidence of associated tissue damage (either microscopically or as  an infiltrate or lesion by imaging)  OR  **Culture of sterile material:** Recovery of *Aspergillus* by culture from a  sample obtained by a sterile procedure from a normally sterile and clinically or  radiologically abnormal site consistent with an infectious disease process, excluding  BAL, paranasal or mastoid sinus cavity, and urine |
| **PROBABLE invasive aspergillosis** |
| At least 1 of the following **host factors**:   - Recent (i.e., within 30 days prior to screening) history of neutropenia (<0.5×10^9^ neutrophils/L [<500 neutrophils/mm^3^]) of any duration temporally related to the onset of fungal disease. - Hematologic malignancy (active malignancy, undergoing treatment for this malignancy, or remission in the recent past) - Receipt of solid organ transplant - Receipt of an allogeneic HSCT - Treatment with other recognized T-cell immune suppressants (e.g., calcineurin inhibitors, tumor necrosis factor-a blockers, lymphocyte specific monoclonal antibodies, or immunosuppressive nucleoside analogues) during the past 90 days - Prolonged use of corticosteroids (excluding among patients with allergic bronchopulmonary aspergillosis) at a mean minimum dose of ≥0.3 mg/kg/day of prednisone equivalent for ≥3 weeks during the past 60 days - Treatment with recognized B-cell immunosuppressants (e.g., Bruton’s tyrosine kinase inhibitors) - Acute graft-versus-host disease grade III or IV (involving the gut, lungs, or liver) that is refractory to first-line treatment with steroids - Inherited severe immunodeficiency (e.g., chronic granulomatous disease, severe combined immunodeficiency)   AND  At least 1 of the following **clinical criteria** (must be temporally related to the current episode of suspected aspergillosis):   - Pulmonary infection: Presence of 1 of the following signs on chest CT (every reasonable attempt should be made to exclude an alternative etiology)   - Dense, well-circumscribed lesions(s) with or without a halo sign   - Air-crescent sign   - Cavity   - Wedge-shaped and segmental or lobar consolidation - Tracheobronchitis: Tracheobronchial ulceration, nodule, pseudomembrane, plaque, or eschar seen on bronchoscopic analysis - Sinonasal infection:   - Acute localized pain (including pain radiating to the eye)   - Nasal ulcer with black eschar   - Extension from the paranasal sinus across bony barriers, including into the orbit - CNS infection: At least 1 of the following 2 signs   - Focal lesions on imaging   - Meningeal enhancement on MRI or CT   AND  At least 1 of the following **mycologic criteria**:   - Direct test   - Pulmonary infection:     - *Aspergillus* recovered by culture from sputum, BAL, bronchial brush, or aspirate     - Microscopical detection of fungal elements in sputum, BAL, bronchial brush, or aspirate indicating a mold   - Tracheobronchitis:     - *Aspergillus* recovered by culture from BAL or bronchial brush     - Microscopic detection of fungal elements in BAL or bronchial brush     - indicating a mold   - Sinonasal infection:     - Mold recovered by culture of sinus aspirate samples     - Microscopic detection of fungal elements in sinus aspirate samples indicating a mold - Indirect test   - Galactomannan detected in plasma, serum, BAL, or CSF     - Single serum or plasma ≥1.0     - BAL fluid ≥1.0     - Single serum or plasma ≥0.7 with BAL fluid ≥0.8     - CSF ≥1.0   - *Aspergillus* PCR     - Plasma, serum, or whole blood ≥2 consecutive PCR tests positive     - BAL fluid ≥2 duplicate PCR tests positive     - At least 1 PCR test positive in plasma, serum, or whole blood and ≥1 PCR test positive in BAL fluid |
| **POSSIBLE invasive fungal disease** |
| At least 1 of the following **host factors**:   - Recent history of neutropenia (<0.5×10^9^ neutrophils/L [<500 neutrophils/mm^3^]) of any duration but temporally related to the onset of fungal disease. - Hematologic malignancy (active malignancy, undergoing treatment for this malignancy, or remission in the recent past) - Receipt of solid organ transplant - Receipt of an allogeneic HSCT - Treatment with other recognized T-cell immune suppressants (e.g., calcineurin inhibitors, tumor necrosis factor-a blockers, lymphocyte specific monoclonal antibodies, or immunosuppressive nucleoside analogues) during the past 90 days - Prolonged use of corticosteroids (excluding among patients with allergic bronchopulmonary aspergillosis) at a mean minimum dose of ≥0.3 mg/kg/day of prednisone equivalent for ≥3 weeks during the past 60 days - Treatment with recognized B-cell immunosuppressants (e.g., Bruton’s tyrosine kinase inhibitors) - Acute graft-versus-host disease grade III or IV (involving the gut, lungs, or liver) that is refractory to first-line treatment with steroids - Inherited severe immunodeficiency (e.g., chronic granulomatous disease, severe combined immunodeficiency)   AND  At least 1 of the following **clinical criteria** (must be temporally related to the current episode of suspected aspergillosis):   - *Pulmonary infection*: Presence of 1 of the following signs on chest CT (every reasonable attempt should be made to exclude an alternative etiology)   - Dense, well-circumscribed lesions(s) with or without a halo sign   - Air-crescent sign   - Cavity   - Wedge-shaped and segmental or lobar consolidation - *Tracheobronchitis*: Tracheobronchial ulceration, nodule, pseudomembrane, plaque, or eschar seen on bronchoscopic analysis - *Sinonasal infection*:   - Acute localized pain (including pain radiating to the eye)   - Nasal ulcer with black eschar   - Extension from the paranasal sinus across bony barriers, including into the orbit - *CNS infection*: At least 1 of the following 2 signs   - Focal lesions on imaging   - Meningeal enhancement on MRI or CT |

# Table S2: Definitions for global clinical response

| **Outcome/Response** | **Definition** |
| --- | --- |
| Success | |
| Complete response | Survival within the prespecified period of observation, resolution of all attributable symptoms and signs of disease, resolution of radiological lesion(s), and documented clearance of infected sites that are accessible to repeated sampling. |
| Partial response | Survival within the prespecified period of observation, improvement in attributable symptoms and signs of disease, improvement of radiological lesion(s),^a^ and evidence of clearance of infected sites that are accessible to repeated sampling. In the case of radiological stabilization,^b^ resolution of all attributable symptoms and signs of fungal disease; or where biopsy of an infected site shows no evidence of hyphae; or where culture is negative. |
| Failure | |
| Stable response | Survival within the prespecified period of observation and minor or no improvement in fungal disease; or persistent isolation of *Aspergillus* or histological presence in infected sites. |
| Progression of fungal disease | Worsening of clinical symptoms and signs of disease plus new sites of disease or radiological worsening; or persistent isolation of *Aspergillus* from infected sites. |
| Death | Death during the prespecified period of evaluation, regardless of attribution to invasive fungal infection or not. |

^a^Improvement of radiological lesions is defined as at least 25% reduction in diameter of radiological lesion. ^b^Radiological stabilization is defined as 0%-25% reduction in the diameter of the lesion.

# Table S3: Disposition of all enrolled participants

|  | **2 to <12 years old** | | **12 to <18 years old** | | **Total** | |
| --- | --- | --- | --- | --- | --- | --- |
|  | n | (%) | n | (%) | n | (%) |
| Participants | 14 | - | 17 | - | 31 | - |
| **Study medication disposition** | | | | | | |
| Started | 14 | (100.0%) | 17 | (100.0%) | 31 | (100.0%) |
| Completed | 1 | (7.1%) | 9 | (52.9%) | 10 | (32.3%) |
| Discontinued | 13 | (92.9%) | 8 | (47.1%) | 21 | (67.7%) |
| Adverse event | 1 | (7.1%) | 1 | (5.9%) | 2 | (6.5%) |
| Death | 0 | (0.0%) | 3 | (17.6%) | 3 | (9.7%) |
| Investigator decision | 12 | (85.7%) | 4 | (23.5%) | 16 | (51.6%) |
| **Study disposition** | | | | | | |
| Completed | 13 | (92.9%) | 14 | (82.4%) | 27 | (87.1%) |
| Discontinued | 1 | (7.1%) | 3 | (17.6%) | 4 | (12.9%) |
| Death | 1 | (7.1%) | 3 | (17.6%) | 4 | (12.9%) |

# Table S4: Full listing of adverse events (regardless of assumed relationship to study intervention) from first dose of posaconazole up to and including 14 days after the last dose in the safety population

|  | **2 to <12 years old** | | | **12 to <18 years old** | | | **Total** | |
| --- | --- | --- | --- | --- | --- | --- | --- | --- |
|  | n | | (%) | n | (%) | | n | (%) |
| Participants in population | 14 | |  | 17 |  | | 31 |  |
| with one or more adverse events | 12 | | (85.7) | 15 | (88.2) | | 27 | (87.1) |
| with no adverse events | 2 | | (14.3) | 2 | (11.8) | | 4 | (12.9) |
| **Blood and lymphatic system disorders** | **5** | | **(35.7)** | **7** | **(41.2)** | | **12** | **(38.7)** |
| Anemia | 1 | | (7.1) | 2 | (11.8) | | 3 | (9.7) |
| Febrile neutropenia | 2 | | (14.3) | 3 | (17.6) | | 5 | (16.1) |
| Lymphocytosis | 0 | | (0.0) | 1 | (5.9) | | 1 | (3.2) |
| Myelosuppression | 0 | | (0.0) | 1 | (5.9) | | 1 | (3.2) |
| Neutropenia | 0 | | (0.0) | 2 | (11.8) | | 2 | (6.5) |
| Thrombocytopenia | 1 | | (7.1) | 1 | (5.9) | | 2 | (6.5) |
| Thrombotic microangiopathy | 1 | | (7.1) | 0 | (0.0) | | 1 | (3.2) |
| **Cardiac disorders** | **3** | | **(21.4)** | **1** | **(5.9)** | | **4** | **(12.9)** |
| Bradycardia | 1 | | (7.1) | 0 | (0.0) | | 1 | (3.2) |
| Sinus bradycardia | 1 | | (7.1) | 1 | (5.9) | | 2 | (6.5) |
| Tachycardia | 2 | | (14.3) | 0 | (0.0) | | 2 | (6.5) |
| **Congenital, familial and genetic disorders** | **1** | | **(7.1)** | **0** | **(0.0)** | | **1** | **(3.2)** |
| Aplasia | 1 | | (7.1) | 0 | (0.0) | | 1 | (3.2) |
| **Endocrine disorders** | **1** | | **(7.1)** | **0** | **(0.0)** | | **1** | **(3.2)** |
| Inappropriate antidiuretic hormone secretion | 1 | | (7.1) | 0 | (0.0) | | 1 | (3.2) |
| **Eye disorders** | **1** | | **(7.1)** | **0** | **(0.0)** | | **1** | **(3.2)** |
| Eyelid oedema | 1 | (7.1) | | 0 | | (0.0) | 1 | (3.2) |
| Oculogyric crisis | 1 | (7.1) | | 0 | | (0.0) | 1 | (3.2) |
| **Gastrointestinal disorders** | **7** | **(50.0)** | | **13** | | **(76.5)** | **20** | **(64.5)** |
| Abdominal pain | 4 | (28.6) | | 2 | | (11.8) | 6 | (19.4) |
| Abdominal pain lower | 0 | (0.0) | | 1 | | (5.9) | 1 | (3.2) |
| Abdominal pain upper | 0 | (0.0) | | 2 | | (11.8) | 2 | (6.5) |
| Anal fissure | 1 | (7.1) | | 1 | | (5.9) | 2 | (6.5) |
| Colitis | 0 | (0.0) | | 2 | | (11.8) | 2 | (6.5) |
| Constipation | 2 | (14.3) | | 1 | | (5.9) | 3 | (9.7) |
| Diarrhea | 3 | (21.4) | | 2 | | (11.8) | 5 | (16.1) |
| Hematemesis | 0 | (0.0) | | 2 | | (11.8) | 2 | (6.5) |
| Hematochezia | 0 | (0.0) | | 1 | | (5.9) | 1 | (3.2) |
| Lip dry | 0 | (0.0) | | 1 | | (5.9) | 1 | (3.2) |
| Mouth hemorrhage | 0 | (0.0) | | 1 | | (5.9) | 1 | (3.2) |
| Nausea | 1 | (7.1) | | 4 | | (23.5) | 5 | (16.1) |
| Neutropenic colitis | 1 | (7.1) | | 1 | | (5.9) | 2 | (6.5) |
| Rectal hemorrhage | 1 | (7.1) | | 0 | | (0.0) | 1 | (3.2) |
| Stomatitis | 2 | (14.3) | | 2 | | (11.8) | 4 | (12.9) |
| Vomiting | 3 | (21.4) | | 7 | | (41.2) | 10 | (32.3) |
| **General disorders and administration site conditions** | **5** | **(35.7)** | | **8** | | **(47.1)** | **13** | **(41.9)** |
| Administration site extravasation | 1 | (7.1) | | 0 | | (0.0) | 1 | (3.2) |
| Chills | 1 | (7.1) | | 0 | | (0.0) | 1 | (3.2) |
| Drug withdrawal syndrome | 0 | (0.0) | | 1 | | (5.9) | 1 | (3.2) |
| Fatigue | 1 | (7.1) | | 3 | | (17.6) | 4 | (12.9) |
| Feeling hot | 0 | (0.0) | | 1 | | (5.9) | 1 | (3.2) |
| Hypothermia | 1 | (7.1) | | 0 | | (0.0) | 1 | (3.2) |
| Infusion site thrombosis | 0 | (0.0) | | 1 | | (5.9) | 1 | (3.2) |
| Malaise | 0 | (0.0) | | 1 | | (5.9) | 1 | (3.2) |
| Mucosal inflammation | 0 | (0.0) | | 2 | | (11.8) | 2 | (6.5) |
| Oedema peripheral | 1 | (7.1) | | 1 | | (5.9) | 2 | (6.5) |
| Pain | 0 | (0.0) | | 1 | | (5.9) | 1 | (3.2) |
| Pyrexia | 3 | (21.4) | | 6 | | (35.3) | 9 | (29.0) |
| Systemic inflammatory response syndrome | 0 | (0.0) | | 1 | | (5.9) | 1 | (3.2) |
| **Hepatobiliary disorders** | **1** | **(7.1)** | | **2** | | **(11.8)** | **3** | **(9.7)** |
| Cholestasis | 1 | (7.1) | | 0 | | (0.0) | 1 | (3.2) |
| Hepatotoxicity | 0 | (0.0) | | 1 | | (5.9) | 1 | (3.2) |
| Hypertransaminasemia | 0 | (0.0) | | 1 | | (5.9) | 1 | (3.2) |
| **Immune system disorders** | **0** | **(0.0)** | | **2** | | **(11.8)** | **2** | **(6.5)** |
| Anaphylactic reaction | 0 | (0.0) | | 1 | | (5.9) | 1 | (3.2) |
| Hypogammaglobulinemia | 0 | (0.0) | | 1 | | (5.9) | 1 | (3.2) |
| **Infections and infestations** | **5** | **(35.7)** | | **8** | | **(47.1)** | **13** | **(41.9)** |
| Adenovirus infection | 0 | (0.0) | | 1 | | (5.9) | 1 | (3.2) |
| Adenovirus interstitial nephritis | 0 | (0.0) | | 1 | | (5.9) | 1 | (3.2) |
| BK virus infection | 0 | (0.0) | | 1 | | (5.9) | 1 | (3.2) |
| Conjunctivitis | 0 | (0.0) | | 1 | | (5.9) | 1 | (3.2) |
| Cytomegalovirus infection | 0 | (0.0) | | 1 | | (5.9) | 1 | (3.2) |
| Cytomegalovirus urinary tract infection | 0 | (0.0) | | 1 | | (5.9) | 1 | (3.2) |
| Device related infection | 0 | (0.0) | | 2 | | (11.8) | 2 | (6.5) |
| Device related sepsis | 1 | (7.1) | | 0 | | (0.0) | 1 | (3.2) |
| Gastroenteritis clostridial | 1 | (7.1) | | 0 | | (0.0) | 1 | (3.2) |
| Herpes zoster | 0 | (0.0) | | 1 | | (5.9) | 1 | (3.2) |
| Infectious disease carrier | 0 | (0.0) | | 1 | | (5.9) | 1 | (3.2) |
| Lower respiratory tract infection | 0 | (0.0) | | 1 | | (5.9) | 1 | (3.2) |
| Lymphadenitis bacterial | 1 | (7.1) | | 0 | | (0.0) | 1 | (3.2) |
| Nail infection | 1 | (7.1) | | 0 | | (0.0) | 1 | (3.2) |
| Paronychia | 0 | (0.0) | | 1 | | (5.9) | 1 | (3.2) |
| Pharyngitis | 0 | (0.0) | | 1 | | (5.9) | 1 | (3.2) |
| Pneumonia | 1 | (7.1) | | 1 | | (5.9) | 2 | (6.5) |
| Rhinitis | 0 | (0.0) | | 1 | | (5.9) | 1 | (3.2) |
| Sepsis | 2 | (14.3) | | 1 | | (5.9) | 3 | (9.7) |
| Staphylococcal sepsis | 0 | (0.0) | | 1 | | (5.9) | 1 | (3.2) |
| Upper respiratory tract infection | 0 | (0.0) | | 1 | | (5.9) | 1 | (3.2) |
| Urinary tract infection | 0 | (0.0) | | 1 | | (5.9) | 1 | (3.2) |
| Urinary tract infection enterococcal | 1 | (7.1) | | 0 | | (0.0) | 1 | (3.2) |
| Urinary tract infection viral | 0 | (0.0) | | 1 | | (5.9) | 1 | (3.2) |
| Viraemia | 0 | (0.0) | | 1 | | (5.9) | 1 | (3.2) |
| Viral hemorrhagic cystitis | 0 | (0.0) | | 1 | | (5.9) | 1 | (3.2) |
| Viral sepsis | 0 | (0.0) | | 1 | | (5.9) | 1 | (3.2) |
| Viruria | 0 | (0.0) | | 2 | | (11.8) | 2 | (6.5) |
| **Injury, poisoning and procedural complications** | **2** | **(14.3)** | | **2** | | **(11.8)** | **4** | **(12.9)** |
| Head injury | 1 | (7.1) | | 0 | | (0.0) | 1 | (3.2) |
| Infusion related reaction | 1 | (7.1) | | 0 | | (0.0) | 1 | (3.2) |
| Procedural pain | 0 | (0.0) | | 1 | | (5.9) | 1 | (3.2) |
| Spinal compression fracture | 0 | (0.0) | | 1 | | (5.9) | 1 | (3.2) |
| **Investigations** | **4** | **(28.6)** | | **8** | | **(47.1)** | **12** | **(38.7)** |
| Alanine aminotransferase increased | 0 | (0.0) | | 4 | | (23.5) | 4 | (12.9) |
| Aspartate aminotransferase increased | 0 | (0.0) | | 4 | | (23.5) | 4 | (12.9) |
| Blood bilirubin unconjugated increased | 0 | (0.0) | | 1 | | (5.9) | 1 | (3.2) |
| Blood creatinine increased | 0 | (0.0) | | 1 | | (5.9) | 1 | (3.2) |
| Electrocardiogram QT prolonged | 2 | (14.3) | | 0 | | (0.0) | 2 | (6.5) |
| Gamma-glutamyltransferase increased | 0 | (0.0) | | 1 | | (5.9) | 1 | (3.2) |
| Hepatic enzyme increased | 1 | (7.1) | | 0 | | (0.0) | 1 | (3.2) |
| Liver function test increased | 0 | (0.0) | | 1 | | (5.9) | 1 | (3.2) |
| Neutrophil count decreased | 1 | (7.1) | | 0 | | (0.0) | 1 | (3.2) |
| Platelet count decreased | 0 | (0.0) | | 1 | | (5.9) | 1 | (3.2) |
| Weight decreased | 1 | (7.1) | | 1 | | (5.9) | 2 | (6.5) |
| Weight increased | 0 | (0.0) | | 1 | | (5.9) | 1 | (3.2) |
| **Metabolism and nutrition disorders** | **4** | **(28.6)** | | **7** | | **(41.2)** | **11** | **(35.5)** |
| Decreased appetite | 2 | (14.3) | | 3 | | (17.6) | 5 | (16.1) |
| Hyperglycemia | 1 | (7.1) | | 1 | | (5.9) | 2 | (6.5) |
| Hypoalbuminemia | 2 | (14.3) | | 0 | | (0.0) | 2 | (6.5) |
| Hypocalcemia | 0 | (0.0) | | 1 | | (5.9) | 1 | (3.2) |
| Hypokalemia | 2 | (14.3) | | 1 | | (5.9) | 3 | (9.7) |
| Hypomagnesaemia | 1 | (7.1) | | 1 | | (5.9) | 2 | (6.5) |
| Hyponatremia | 1 | (7.1) | | 0 | | (0.0) | 1 | (3.2) |
| Hypophosphatasemia | 1 | (7.1) | | 0 | | (0.0) | 1 | (3.2) |
| Hypoproteinemia | 1 | (7.1) | | 0 | | (0.0) | 1 | (3.2) |
| **Musculoskeletal and connective tissue disorders** | **1** | **(7.1)** | | **8** | | **(47.1)** | **9** | **(29.0)** |
| Arthralgia | 0 | (0.0) | | 4 | | (23.5) | 4 | (12.9) |
| Back pain | 0 | (0.0) | | 1 | | (5.9) | 1 | (3.2) |
| Flank pain | 0 | (0.0) | | 1 | | (5.9) | 1 | (3.2) |
| Muscle spasms | 0 | (0.0) | | 1 | | (5.9) | 1 | (3.2) |
| Muscular weakness | 0 | (0.0) | | 1 | | (5.9) | 1 | (3.2) |
| Musculoskeletal pain | 0 | (0.0) | | 1 | | (5.9) | 1 | (3.2) |
| Myalgia | 1 | (7.1) | | 3 | | (17.6) | 4 | (12.9) |
| Pain in extremity | 0 | (0.0) | | 3 | | (17.6) | 3 | (9.7) |
| **Neoplasms benign, malignant and unspecified (incl cysts and polyps)** | **1** | **(7.1)** | | **0** | | **(0.0)** | **1** | **(3.2)** |
| Leukemic infiltration extramedullary | 1 | (7.1) | | 0 | | (0.0) | 1 | (3.2) |
| **Nervous system disorders** | **0** | **(0.0)** | | **6** | | **(35.3)** | **6** | **(19.4)** |
| Dizziness | 0 | (0.0) | | 1 | | (5.9) | 1 | (3.2) |
| Dysgeusia | 0 | (0.0) | | 1 | | (5.9) | 1 | (3.2) |
| Headache | 0 | (0.0) | | 4 | | (23.5) | 4 | (12.9) |
| Neuralgia | 0 | (0.0) | | 1 | | (5.9) | 1 | (3.2) |
| Somnolence | 0 | (0.0) | | 1 | | (5.9) | 1 | (3.2) |
| **Psychiatric disorders** | **1** | **(7.1)** | | **3** | | **(17.6)** | **4** | **(12.9)** |
| Anxiety | 0 | (0.0) | | 1 | | (5.9) | 1 | (3.2) |
| Delirium | 0 | (0.0) | | 2 | | (11.8) | 2 | (6.5) |
| Depressed mood | 0 | (0.0) | | 1 | | (5.9) | 1 | (3.2) |
| Depression | 1 | (7.1) | | 0 | | (0.0) | 1 | (3.2) |
| Insomnia | 0 | (0.0) | | 1 | | (5.9) | 1 | (3.2) |
| **Renal and urinary disorders** | **0** | **(0.0)** | | **6** | | **(35.3)** | **6** | **(19.4)** |
| Acute kidney injury | 0 | (0.0) | | 2 | | (11.8) | 2 | (6.5) |
| Bladder spasm | 0 | (0.0) | | 1 | | (5.9) | 1 | (3.2) |
| Dysuria | 0 | (0.0) | | 2 | | (11.8) | 2 | (6.5) |
| Hematuria | 0 | (0.0) | | 3 | | (17.6) | 3 | (9.7) |
| Hydronephrosis | 0 | (0.0) | | 1 | | (5.9) | 1 | (3.2) |
| Kidney enlargement | 0 | (0.0) | | 1 | | (5.9) | 1 | (3.2) |
| Nephrolithiasis | 0 | (0.0) | | 2 | | (11.8) | 2 | (6.5) |
| Nephropathy toxic | 0 | (0.0) | | 2 | | (11.8) | 2 | (6.5) |
| Pollakiuria | 0 | (0.0) | | 1 | | (5.9) | 1 | (3.2) |
| Pyelocaliectasis | 0 | (0.0) | | 1 | | (5.9) | 1 | (3.2) |
| Renal hemorrhage | 0 | (0.0) | | 1 | | (5.9) | 1 | (3.2) |
| Renal tubular disorder | 0 | (0.0) | | 1 | | (5.9) | 1 | (3.2) |
| Urinary bladder hemorrhage | 0 | (0.0) | | 1 | | (5.9) | 1 | (3.2) |
| Urinary incontinence | 0 | (0.0) | | 1 | | (5.9) | 1 | (3.2) |
| Urinary retention | 0 | (0.0) | | 1 | | (5.9) | 1 | (3.2) |
| **Reproductive system and breast disorders** | **0** | **(0.0)** | | **2** | | **(11.8)** | **2** | **(6.5)** |
| Genital pain | 0 | (0.0) | | 1 | | (5.9) | 1 | (3.2) |
| Testicular pain | 0 | (0.0) | | 1 | | (5.9) | 1 | (3.2) |
| **Respiratory, thoracic and mediastinal disorders** | **6** | **(42.9)** | | **6** | | **(35.3)** | **12** | **(38.7)** |
| Cough | 0 | (0.0) | | 1 | | (5.9) | 1 | (3.2) |
| Dyspnea | 0 | (0.0) | | 1 | | (5.9) | 1 | (3.2) |
| Epistaxis | 2 | (14.3) | | 1 | | (5.9) | 3 | (9.7) |
| Hemoptysis | 0 | (0.0) | | 1 | | (5.9) | 1 | (3.2) |
| Hyperventilation | 0 | (0.0) | | 1 | | (5.9) | 1 | (3.2) |
| Oropharyngeal pain | 1 | (7.1) | | 1 | | (5.9) | 2 | (6.5) |
| Pulmonary hemorrhage | 0 | (0.0) | | 1 | | (5.9) | 1 | (3.2) |
| Pulmonary hypertension | 1 | (7.1) | | 0 | | (0.0) | 1 | (3.2) |
| Pulmonary oedema | 1 | (7.1) | | 0 | | (0.0) | 1 | (3.2) |
| Respiratory failure | 1 | (7.1) | | 0 | | (0.0) | 1 | (3.2) |
| Wheezing | 1 | (7.1) | | 0 | | (0.0) | 1 | (3.2) |
| **Skin and subcutaneous tissue disorders** | **4** | **(28.6)** | | **4** | | **(23.5)** | **8** | **(25.8)** |
| Dermatitis | 0 | (0.0) | | 1 | | (5.9) | 1 | (3.2) |
| Dermatitis acneiform | 0 | (0.0) | | 1 | | (5.9) | 1 | (3.2) |
| Dermatitis contact | 1 | (7.1) | | 0 | | (0.0) | 1 | (3.2) |
| Hyperhidrosis | 0 | (0.0) | | 1 | | (5.9) | 1 | (3.2) |
| Rash | 2 | (14.3) | | 0 | | (0.0) | 2 | (6.5) |
| Rash (popular) | 1 | (7.1) | | 0 | | (0.0) | 1 | (3.2) |
| Skin ulcer | 0 | (0.0) | | 1 | | (5.9) | 1 | (3.2) |
| **Vascular disorders** | **4** | **(28.6)** | | **6** | | **(35.3)** | **10** | **(32.3)** |
| Hypertension | 3 | (21.4) | | 5 | | (29.4) | 8 | (25.8) |
| Hypotension | 1 | (7.1) | | 0 | | (0.0) | 1 | (3.2) |
| Vena cava thrombosis | 0 | (0.0) | | 1 | | (5.9) | 1 | (3.2) |
| Every participant is counted a single time for each applicable row and column. Adverse events were followed for up to and including 14 days after the last dose. Medical Dictionary for Regulatory Activities (MedDRA) version 26.1 was used in the adverse event reporting for this study. | | | | | | | | |

# Table S5: Full listing of serious adverse events (regardless of assumed relationship to study intervention) from first dose of posaconazole up to and including 14 days after the last dose in the safety population

|  | **2 to <12 years old** | | **12 to <18 years old** | | **Total** | |
| --- | --- | --- | --- | --- | --- | --- |
|  | n | (%) | n | (%) | n | (%) |
| Participants in population | 14 |  | 17 |  | 31 |  |
| with one or more adverse events | 4 | (28.6) | 8 | (47.1) | 12 | (38.7) |
| with no adverse events | 10 | (71.4) | 9 | (52.9) | 19 | (61.3) |
| **Blood and lymphatic system disorders** | **2** | **(14.3)** | **2** | **(11.8)** | **4** | **(12.9)** |
| Anemia | 1 | (7.1) | 0 | (0.0) | 1 | (3.2) |
| Febrile neutropenia | 1 | (7.1) | 1 | (5.9) | 2 | (6.5) |
| Thrombocytopenia | 0 | (0.0) | 1 | (5.9) | 1 | (3.2) |
| **Gastrointestinal disorders** | **1** | **(7.1)** | **1** | **(5.9)** | **2** | **(6.5)** |
| Hematemesis | 0 | (0.0) | 1 | (5.9) | 1 | (3.2) |
| Stomatitis | 1 | (7.1) | 0 | (0.0) | 1 | (3.2) |
| **General disorders and administration site conditions** | **1** | **(7.1)** | **1** | **(5.9)** | **2** | **(6.5)** |
| Administration site extravasation | 1 | (7.1) | 0 | (0.0) | 1 | (3.2) |
| Mucosal inflammation | 0 | (0.0) | 1 | (5.9) | 1 | (3.2) |
| **Infections and infestations** | **3** | **(21.4)** | **3** | **(17.6)** | **6** | **(19.4)** |
| Cytomegalovirus infection | 0 | (0.0) | 1 | (5.9) | 1 | (3.2) |
| Device related sepsis | 1 | (7.1) | 0 | (0.0) | 1 | (3.2) |
| Gastroenteritis clostridial | 1 | (7.1) | 0 | (0.0) | 1 | (3.2) |
| Lymphadenitis bacterial | 1 | (7.1) | 0 | (0.0) | 1 | (3.2) |
| Sepsis | 1 | (7.1) | 1 | (5.9) | 2 | (6.5) |
| Viral sepsis | 0 | (0.0) | 1 | (5.9) | 1 | (3.2) |
| **Investigations** | **0** | **(0.0)** | **1** | **(5.9)** | **1** | **(3.2)** |
| Weight decreased | 0 | (0.0) | 1 | (5.9) | 1 | (3.2) |
| **Neoplasms benign, malignant and unspecified (incl cysts and polyps)** | **1** | **(7.1)** | **0** | **(0.0)** | **1** | **(3.2)** |
| Leukemic infiltration extramedullary | 1 | (7.1) | 0 | (0.0) | 1 | (3.2) |
| **Renal and urinary disorders** | **0** | **(0.0)** | **3** | **(17.6)** | **3** | **(9.7)** |
| Nephrolithiasis | 0 | (0.0) | 1 | (5.9) | 1 | (3.2) |
| Nephropathy toxic | 0 | (0.0) | 1 | (5.9) | 1 | (3.2) |
| Urinary bladder hemorrhage | 0 | (0.0) | 1 | (5.9) | 1 | (3.2) |
| **Respiratory, thoracic and mediastinal disorders** | **1** | **(7.1)** | **1** | **(5.9)** | **2** | **(6.5)** |
| Pulmonary hemorrhage | 0 | (0.0) | 1 | (5.9) | 1 | (3.2) |
| Respiratory failure | 1 | (7.1) | 0 | (0.0) | 1 | (3.2) |
| Every participant is counted a single time for each applicable row and column. Adverse events were followed for up to and including 14 days after the last dose. Medical Dictionary for Regulatory Activities (MedDRA) version 26.1 was used in the reporting of this study. | | | | | | |

# Table S6: Summary of palatability and acceptance of posaconazole powder for oral suspension in all participants who were treated with this formulation

|  | **First Day** | **Last Day** |
| --- | --- | --- |
|  | n (%) | n (%) |
| Number of Participants Completing Palatability Questionnaire | 10 | 10 |
| **Person Completing the Palatability Questionnaire** | | |
| The health care provider (e.g., physician, nurse, medical assistant or nursing assistant caring for the participant) | 2 (20.0) | 2 (20.0) |
| The parent/primary caregiver | 4 (40.0) | 4 (40.0) |
| The participant | 3 (30.0) | 3 (30.0) |
| The participant and parent/primary caregiver | 1 (10.0) | 1 (10.0) |
| **Taste** | | |
| Very good | 2 (20.0) | 1 (10.0) |
| Good | 4 (40.0) | 4 (40.0) |
| Very bad | 1 (10.0) | 1 (10.0) |
| Neither good nor bad | 3 (30.0) | 4 (40.0) |
| **Problems Taking Dose** | | |
| Any problem | 0 (0.0) | 0 (0.0) |
| Refusing | 0 (0.0) | 0 (0.0) |
| Spitting out | 0 (0.0) | 0 (0.0) |
| Vomiting or spitting up | 0 (0.0) | 0 (0.0) |
| Gagging | 0 (0.0) | 0 (0.0) |
| Other | 0 (0.0) | 0 (0.0) |
| Percentages are based on the number of participants completing the palatability questionnaire on the first day and last day of treatment with posaconazole powder for oral suspension. | | |
