# Supplementary material for: Phase 2, open-label, noncomparative clinical trial evaluating safety and efficacy of posaconazole in pediatric patients with proven/probable invasive aspergillosis or possible invasive fungal disease
Source: Antimicrob Agents Chemother. 2026 Jan 27;70(3):e01305-25. doi: 10.1128/aac.01305-25 (PMC12959131; doi:10.1128/aac.01305-25)
Supplement: Supplemental file — MK-5592-104 study protocol. [file aac.01305-25-s0002.pdf]

This protocol may have been redacted to remove any information that is proprietary to Merck & Co., Inc., Rahway, NJ, USA.

This report may include approved and non-approved uses, formulations, or treatment regimens. The results reported may not reflect the overall profile of a product. Before prescribing any product mentioned in this report, healthcare professionals should consult local prescribing information for the product approved in their country.

Copyright © 2025 Merck & Co., Inc., Rahway, NJ, USA and its affiliates. All rights reserved.
Not for regulatory or commercial use.

Title Page

THIS PROTOCOL AMENDMENT AND ALL OF THE INFORMATION RELATING TO IT ARE CONFIDENTIAL AND PROPRIETARY PROPERTY OF MERCK SHARP & DOHME LLC, RAHWAY, NJ, USA (MSD).

Protocol Title: A Phase 2, Open-Label, Non-Comparative Clinical Trial to Study the Safety and Efficacy of Posaconazole (POS, MK-5592) in Pediatric Participants Aged 2 to Less Than 18 Years With Invasive Aspergillosis

Protocol Number: 104-04

Compound Number: MK-5592

Sponsor Name:

Merck Sharp & Dohme LLC
(hereafter referred to as the Sponsor or MSD)

Legal Registered Address:

126 East Lincoln Avenue

P.O. Box 2000

Rahway, NJ 07065 USA

Regulatory Agency Identifying Number(s):

IND	51,316; 75,061; 125,097
EudraCT	2019-002267-10

Approval Date: 02 September 2022

Sponsor Signatory

Typed Name: Date
Title:

Protocol-specific Sponsor contact information can be found in the Investigator Study File Binder (or equivalent).

Investigator Signatory

I agree to conduct this clinical study in accordance with the design outlined in this protocol and to abide by all provisions of this protocol.

Typed Name: Date
Title:

DOCUMENT HISTORY

Document	Date of Issue	Overall Rationale
Amendment 04	02-SEP-2022	To revise exclusion criteria to include the specific K values used in the modified Schwartz formula for males of different ages.
Amendment 03	19-JUL-2022	To 1) align dosing of the powder for suspension formulation with the dosing algorithm given in the approved product labeling for pediatric use, 2) update the definitions of possible, probable, or proven IA to align with the 2020 EORTC/MSG criteria, and 3) update the prohibited and allowed medications lists to align with approved product labeling.
Amendment 02	15-DEC-2020	To provide details for IV dosing requirements; to clarify procedures (ie, ECG and diagnostic imaging) in the Schedule of Activities; to clarify maximum blood volume and method for calculating creatinine clearance; to remove pregnancy exclusion to avoid potential confusion with the pregnancy criteria in the list of inclusions; and to update informed consent text to align with current informed consent/assent procedures.
Amendment 01	21-APR-2020	To remove the device-related adverse event reporting language and the Future Biomedical Research (FBR) substudy from the protocol.
Original Protocol	17-JUL-2019	Not applicable

PROTOCOL AMENDMENT SUMMARY OF CHANGES

Amendment: 04

Overall Rationale for the Amendments:

To revise exclusion criteria to include the specific K values used in the modified Schwartz formula for males of different ages.

Summary of Changes Table:

Section # and Name	Description of Change	Brief Rationale
5.2 Exclusion Criteria	Exclusion criterion 13 was revised to show the proportionality constant (K) separately for pre-and post-adolescent male children and to change the lower bound of the age range for male and female children from 1 year to 2 years.	To revise exclusion criteria to include the specific K values used in the modified Schwartz formula for males of different ages.
1.1 Synopsis<br>1.2 Schema<br>4.3.1 Starting Dose for This Study<br>6.1 Study Intervention(s) Administered	Added Table 1 to outline weight-band dosing in Sec. 4.3.1 and added reference to Table 1 in other sections outlined here.	To align the dosing of the PFS formulation with approved product labeling for the PFS when used in the pediatric population and achieve a similar target exposure for all participants.

Section # and Name	Description of Change	Brief Rationale
1.1 Synopsis<br>1.2 Schema<br>1.3 Schedule of Activities<br>3 Hypotheses, objectives, and endpoints<br>4.1 Overall Design<br>4.2.1.1 Efficacy Endpoints<br>5.1 Inclusion Criteria<br>8.1.4 Medical History<br>8.1.5.1 Prior Medications8.2.2 Diagnostic Imaging<br>8.2.3 Mycology Testing<br>8.2.4 IFI Assessment<br>8.2.7 *Aspergillus* Galactomannan EIA and PCR<br>9.5.1 Efficacy Analysis Population<br>10.9 Appendix 9	• Updated definitions used for IA disease classification and included possible in addition to proven and probable IA.<br>• Added statement to say that medical history should include any history of graft vs host disease.<br>• Clarified text about the recording of chemotherapeutic agents for active treatment of malignancy, as well as T-cell and B-cell immunosuppressive therapies.<br>• Text added to ensure that recording of imaging includes sufficient details to reflect use of the EORTC/MSG 2020 criteria to support the classification of IA.<br>• Secondary objectives were revised to add 'possible' to IA.<br>• Added note in SoA for optional PCR testing in the Mycology testing row.	Updated to reflect the 2020 EORTC/MSG criteria's classification of possible, probable, or proven IA.

Section # and Name	Description of Change	Brief Rationale
6.5 Concomitant Therapy<br>6.5.1 Medications Allowed with Caution and Clinical Monitoring	Updated Table 4 Prohibited Medications During Study Treatment:<br>• Relocated the following prohibited medications to medications allowed with caution: anthracycline, trisenox, cyclophosphamide, vinca alkaloids, and sirolimus.<br>• Added venetoclax to the prohibited medication list.<br>Updated Table 5 list of medications allowed with caution and monitoring to add anthracycline, trisenox, cyclophosphamide, vinca alkaloids, and sirolimus.<br>Updated Table 5 Additional Medications Allowed During the Study That Require Monitoring and/or Dose Adjustment to add antineoplastics, chemotherapy agents, and tyrosine kinase inhibitors.	To align with allowable/prohibited medications as noted in approved product labeling with the inclusion of cautionary language.

Section # and Name	Description of Change	Brief Rationale
2.1 Study Rationale<br>2.2.1 Pharmaceutical and Therapeutic Background<br>2.3 Benefit/Risk Assessment<br>4.2 Scientific Rationale for Study Design<br>4.2.2 Rationale for the Use of Comparator/Placebo<br>4.3.3 Rationale for Dose Interval and Study Design	Updated text to reflect data from completed studies and current product labeling:<br>• Added clinical study data and approval regarding POS IV and tablet for the primary treatment of IA.<br>• Added clinical study data and approval regarding POS IV, PFS, and tablet for use in pediatric patients 2 to <18 years of age in the salvage treatment and the prophylaxis of invasive fungal infections.<br>• Updated text to reflect new pediatric and adult efficacy and safety information for IV, tablet, and PFS formulations.	Updated background/rationale to reflect additional study data/status and new health authority approvals for pediatric and adult patients.
1.1 Synopsis<br>4.1 Overall Design	Updated wording for evaluable participants for the efficacy evaluation in each age cohort.	Clarified that the FAS population includes study participants with possible, probable, or proven IA and who have at least 1 postrandomization assessment. The inclusion of possible, probable, or proven IA as the population to be analyzed in the efficacy analyses reflects the use of a similar population used in the primary efficacy analyses of the adult study (P069) of posaconazole in the treatment of invasive aspergillosis. Data from that study were used to support the registration of posaconazole IV and oral for the treatment of invasive aspergillosis in adults.

Section # and Name	Description of Change	Brief Rationale
5.2 Exclusion Criteria	• Updated Table 2 Prohibited Medications Before Start of Study Treatment.<br>• Removed Gilbert's disease.<br>• Added hereditary fructose intolerance.<br>• Removed use of CPAP/BPAP as an exclusion criterion.	• To align the allowed and prohibited medications with the most recent approved product labeling.<br>• Current approved product labeling allows posaconazole to be given to patients with Gilbert's disease.<br>• Approved product labeling recommends against the use of posaconazole PFS in patients with hereditary fructose intolerance due to the presence of sucrose and sorbitol in the suspending vehicle that is admixed with the PFS powder.<br>• Allowance of CPAP/BPAP reflects the potential use of these modalities in patients with pulmonary aspergillosis and respiratory insufficiency.
2.2.3 Ongoing Clinical Studies	Updated to reflect that there are currently only 2 ongoing studies for posaconazole, this study (P104) and a Phase 2 study (P127).	Updated to reflect the current status of ongoing clinical studies.
4.2.1.2 Safety Endpoints	Clarified that the primary study endpoint is the safety of POS in the pediatric population as assessed by the evaluation of treatment-related AEs. Other assessments may include all AEs, ECGs, and laboratory tests.	Clarified the wording in this section to align with the wording in the SAP related to the primary study endpoint.

Section # and Name	Description of Change	Brief Rationale
5.1 Inclusion Criteria	Modified Inclusion Criterion 7 to require that participants must weigh at least 10 kg to be included in the study.	The restriction to be placed on inclusion of participants who weigh at least 10 kg reflects the approved labeled dosing table for the PFS that requires children 2 to <18 years of age to weigh at least 10 kg to receive PFS dosing.
7.1 Discontinuation of Study Intervention	Updated text to allow study intervention to be interrupted for up to 3 days with repeat monitoring of liver function testing. Study intervention may be resumed if repeated liver function testing parameters decreases to lower values.	Transient interruption of study medication is considered as appropriate clinical management of the study participants if the liver function testing parameter abnormalities are transient, and if the abnormalities are judged unrelated to study drug.
9.1 Statistical Analysis Plan	Updated to reflect amendment changes.	Updated for clarity.
1.1 Synopsis<br>3 Hypotheses, Objectives, and Endpoints<br>9.4.1.2 Pharmacokinetic Endpoints<br>9.6.2 Pharmacokinetic Analysis	Updated sections to explain that modeling analysis will be based on actual dose administered, and that certain PK parameters will be reported in a separate report.	The updated text provides additional details that the modeling analyses will be based on the dose of IV and oral formulations given.
9.6.3 Safety Analysis	Updated text to specify that safety summaries will be presented in aggregate.	The updated text clarified the data presentation format for the summary of safety analyses.

Section # and Name	Description of Change	Brief Rationale
8.2.1 Global Clinical Response<br>9.9.1 Efficacy Analysis	• Global clinical response definition expanded in text.<br>• Updated text to outline the global clinical response seen in adolescents and adults.<br>• Updated Observed Global Clinical Response Table 9 to remove first 2 rows.	• To more clearly define global clinical response.<br>• To align with observed data in trials/literature as it relates to expected clinical response.<br>• Based upon data from the adult study (P069), the observed global clinical response rates in participants with invasive aspergillosis after 12 weeks of therapy are expected to be in the range of 40%, thus rates higher than 60% have been removed from Table 9.
8.9.1 Screening	• Clarified text to say that informed consent/assent must be obtained before any study-specific procedures are performed other than those procedures conducted for the purpose of routine clinical care.<br>• Added imaging and mycology testing to the list of local laboratory tests.	• The text related to informed consent was modified to clarify the timeframe in which study-specific procedures may be obtained relative to the signing of the informed consent/assent.<br>• There was a clarification as to the source of imaging and mycology testing to indicate that local testing results would be used. These tests will be conducted to support the disease diagnosis and classification of possible, probable, or proven invasive aspergillosis.
8.9.2.3 End of Treatment (EOT) Visits	Clarified that participants who withdraw consent for participation or who are withdrawn from the study for any reason should complete both the EOT Visit and the final mortality assessment at Day 114.	Updated for clarity.

Section # and Name	Description of Change	Brief Rationale
1.1 Synopsis<br>8.1.1 Informed Consent/Assent<br>8.1.1.1 General Informed Consent/Assent<br>8.9.1 Screening	Modified to ensure that assent is used in all places where ICF is discussed.	To align with global consent/assent requirements for adolescent and pediatric populations.
Title Page<br>10.1.1 Code of Conduct for Trials	Sponsor entity name and address change.	Merck Sharp & Dohme Corp. underwent an entity name and address change to Merck Sharp & Dohme LLC, Rahway, NJ, USA. This conversion resulted only in an entity name change and update to the address.
4.4 Beginning and End of Study Definition	Added study start definition for European Economic Area countries.	To align with the EU CTR.
8.4 Adverse Events (AEs), Serious Adverse Events (SAEs), and Other Reportable Safety Events<br>10.3.1 Definitions of Medication Error, Misuse, and Abuse	Added section 10.3.1 to define medication error, misuse and abuse and indicate that AEs associated with the above should be reported.	To align with the EU CTR.
10.7 Appendix 7	Remove Czech Republic country-specific language.	The Czech Republic is no longer being considered for participation in the study.

Section # and Name	Description of Change	Brief Rationale
Throughout Document	Minor administrative, formatting, grammatical, and typographical changes were made throughout the document.	To ensure clarity and accurate interpretation of the intent of the protocol.

Table of Contents

DOCUMENT HISTORY 3
PROTOCOL AMENDMENT SUMMARY OF CHANGES 4
1 PROTOCOL SUMMARY 20
1.1 Synopsis 20
1.2 Schema 24
1.3 Schedule of Activities (SoA) 25
2 INTRODUCTION 33
2.1 Study Rationale 33
2.2 Background 34
2.2.1 Pharmaceutical and Therapeutic Background 34
2.2.2 Preclinical and Clinical Studies 34
2.2.3 Ongoing Clinical Studies 35
2.3 Benefit/Risk Assessment 35
3 HYPOTHESES, OBJECTIVES, AND ENDPOINTS 35
4 STUDY DESIGN 37
4.1 Overall Design 37
4.2 Scientific Rationale for Study Design 38
4.2.1 Rationale for Endpoints 38
4.2.1.1 Efficacy Endpoints 38
4.2.1.2 Safety Endpoints 39
4.2.1.3 Pharmacokinetic Endpoints 39
4.2.1.4 Additional Endpoints 39
4.2.2 Rationale for the Use of Comparator/Placebo 39
4.3 Justification for Dose 39
4.3.1 Starting Dose for This Study 39
4.3.2 Maximum Dose/Exposure for This Study 40
4.3.3 Rationale for Dose Interval and Study Design 40
4.4 Beginning and End of Study Definition 41
4.4.1 Clinical Criteria for Early Study Termination 41
5 STUDY POPULATION 41
5.1 Inclusion Criteria 41
5.2 Exclusion Criteria 44
5.3 Lifestyle Considerations 47
5.4 Screen Failures 47
5.5 Participant Replacement Strategy 47

6 STUDY INTERVENTION........48
6.1 Study Intervention(s) Administered........48
6.2 Preparation/Handling/Storage/Accountability........50
6.2.1 Dose Preparation........50
6.2.2 Handling, Storage, and Accountability........50
6.3 Measures to Minimize Bias: Randomization and Blinding........51
6.3.1 Intervention Assignment........51
6.3.2 Stratification........51
6.3.3 Blinding........51
6.4 Study Intervention Compliance........51
6.5 Concomitant Therapy........51
6.5.1 Medications Allowed With Caution and Clinical Monitoring........53
6.5.2 Rescue Medications and Supportive Care........56
6.6 Dose Modification (Escalation/Titration/Other)........56
6.7 Intervention After the End of the Study........56
6.8 Clinical Supplies Disclosure........57
7 DISCONTINUATION OF STUDY INTERVENTION AND PARTICIPANT WITHDRAWAL........57
7.1 Discontinuation of Study Intervention........57
7.2 Participant Withdrawal From the Study........58
7.3 Lost to Follow-up........58
8 STUDY ASSESSMENTS AND PROCEDURES........59
8.1 Administrative and General Procedures........59
8.1.1 Informed Consent/Assent........59
8.1.1.1 General Informed Consent/Assent........60
8.1.2 Inclusion/Exclusion Criteria........60
8.1.3 Participant Identification Card........60
8.1.4 Medical History........61
8.1.5 Prior and Concomitant Medications Review........61
8.1.5.1 Prior Medications........61
8.1.5.2 Concomitant Medications........61
8.1.6 Assignment of Screening Number........61
8.1.7 Assignment of Treatment/Randomization Number........62
8.1.8 Study Intervention Administration........62
8.1.9 Discontinuation and Withdrawal........62
8.1.10 Participant Blinding/Unblinding........63
8.1.11 Calibration of Equipment........63
8.2 Efficacy/Immunogenicity Assessments........63

8.2.1 Global Clinical Response 63
8.2.2 Diagnostic Imaging 64
8.2.3 Mycology Testing 65
8.2.4 IFI Assessment 65
8.2.5 Relapse Assessment 65
8.2.6 Mortality Assessment 65
8.2.7 *Aspergillus* Galactomannan EIA and PCR 65
8.3 Safety Assessments 66
8.3.1 Physical Examinations 66
8.3.2 Vital Signs 67
8.3.3 Electrocardiograms 67
8.3.4 Clinical Safety Laboratory Assessments 67
8.3.5 Assessment of Palatability and Acceptability 68
8.4 Adverse Events (AEs), Serious Adverse Events (SAEs), and Other Reportable Safety Events 68
8.4.1 Time Period and Frequency for Collecting AE, SAE, and Other Reportable Safety Event Information 69
8.4.2 Method of Detecting AEs, SAEs, and Other Reportable Safety Events......71
8.4.3 Follow-up of AE, SAE, and Other Reportable Safety Event Information...71
8.4.4 Regulatory Reporting Requirements for SAE 71
8.4.5 Pregnancy and Exposure During Breastfeeding 71
8.4.6 Disease-related Events and/or Disease-related Outcomes Not Qualifying as AEs or SAEs 72
8.4.7 Events of Clinical Interest (ECIs) 72
8.5 Treatment of Overdose 72
8.6 Pharmacokinetics 72
8.6.1 Blood Collection for Plasma MK-5592 73
8.7 Pharmacodynamics 73
8.8 Future Biomedical Research Sample Collection 73
8.9 Visit Requirements 73
8.9.1 Screening 73
8.9.2 Treatment Period Visits 73
8.9.2.1 Day 1 Visit 73
8.9.2.2 Treatment Period 74
8.9.2.3 End of Treatment (EOT) Visits 74
8.9.3 Post-Treatment Visits 74
9 STATISTICAL ANALYSIS PLAN 74
9.1 Statistical Analysis Plan Summary 74

9.2 Responsibility for Analyses/In-House Blinding 76
9.3 Hypotheses/Estimation 76
9.4 Analysis Endpoints....76
9.4.1 Efficacy/Pharmacokinetic Endpoints....76
9.4.1.1 Efficacy Endpoints....76
9.4.1.2 Pharmacokinetic Endpoints 76
9.4.1.3 Tertiary/Exploratory Endpoints 77
9.4.2 Safety Endpoints 77
9.4.3 Additional Endpoints 77
9.4.4 Derivations of Efficacy/Immunogenicity/Pharmacokinetics Endpoints....77
9.5 Analysis Populations....77
9.5.1 Efficacy Analysis Populations 77
9.5.2 Pharmacokinetic Analysis Population 78
9.5.3 Safety Analysis Populations 78
9.6 Statistical Methods....78
9.6.1 Efficacy Analysis 78
9.6.2 Pharmacokinetic Analysis....78
9.6.3 Safety Analysis 78
9.6.4 Other Analyses....79
9.7 Interim Analyses 80
9.8 Multiplicity 80
9.9 Sample Size and Power Calculations 80
9.9.1 Efficacy Analysis....80
9.9.2 Safety Analysis 80
9.10 Subgroup Analyses....81
9.11 Compliance (Medication Adherence)....81
9.12 Extent of Exposure....82
10 SUPPORTING DOCUMENTATION AND OPERATIONAL CONSIDERATIONS 83
10.1 Appendix 1: Regulatory, Ethical, and Study Oversight Considerations 83
10.1.1 Code of Conduct for Clinical Trials....83
10.1.2 Financial Disclosure....85
10.1.3 Data Protection....85
10.1.3.1 Confidentiality of Data 86
10.1.3.2 Confidentiality of Participant Records....86
10.1.3.3 Confidentiality of IRB/IEC Information....86
10.1.4 Committees Structure....86
10.1.4.1 Executive Oversight Committee 86

10.1.4.2 External Data Monitoring Committee 87
10.1.5 Publication Policy 87
10.1.6 Compliance with Study Registration and Results Posting Requirements ...87
10.1.7 Compliance with Law, Audit, and Debarment 88
10.1.8 Data Quality Assurance 88
10.1.9 Source Documents 89
10.1.10 Study and Site Closure 90
10.2 Appendix 2: Clinical Laboratory Tests 91
10.3 Appendix 3: Adverse Events: Definitions and Procedures for Recording, Evaluating, Follow-up, and Reporting 93
10.3.1 Definitions of Medication Error, Misuse, and Abuse: 93
10.3.2 Definition of AE 93
10.3.3 Definition of SAE 94
10.3.4 Additional Events Reported 95
10.3.5 Recording AE and SAE 96
10.3.6 Reporting of AEs, SAEs, and Other Reportable Safety Events to the Sponsor 99
10.4 Appendix 4: Device Events, Adverse Device Events, and Medical Device Incidents: Definitions, Collection, and Documentation 101
10.5 Appendix 5: Contraceptive Guidance 102
10.5.1 Definitions 102
10.5.2 Contraception Requirements 103
10.6 Appendix 6: Collection and Management of Specimens for Future Biomedical Research 104
10.7 Appendix 7: Country-specific Requirements 105
10.8 Appendix 8: Abbreviations 106
10.9 Appendix 9: Criteria for Diagnosis of Invasive Aspergillosis 108
11 REFERENCES 113

LIST OF TABLES

Table 1 Dosing of the PFS 40
Table 2 Prohibited Medications Before Start of Study Treatment....................45
Table 3 Study Treatments 49
Table 4 Prohibited Medications During Study Treatment....................52
Table 5 Additional Medications Allowed During the Study That Require Monitoring and/or Dose Adjustment 54
Table 6 Global Clinical Response using 2008 EORTC/MSG Definitions 64
Table 7 Reporting Time Periods and Time Frames for Adverse Events and Other Reportable Safety Events....................70
Table 8 Analysis Strategy for Safety Parameters....................79
Table 9 Summary of Potential Observed Global Clinical Response Rates and 95% Confidence Intervals....................80
Table 10 Estimate of Incidence of a Specific AE and Upper Limit of 95% CI Based on a Hypothetical Number of Participants with that Specific AE ..81
Table 11 Protocol-Required Safety and Other Laboratory Assessments....................91
Table 12 Approximate Blood/Tissue Volumes Drawn/Collected by Trial Visit and by Sample Types 92

LIST OF FIGURES

Figure 1 Trial Design 24

1 PROTOCOL SUMMARY

1.1 Synopsis

Protocol Title: A Phase 2, Open-Label, Non-Comparative Clinical Trial to Study the Safety and Efficacy of Posaconazole (POS, MK-5592) in Pediatric Participants Aged 2 to Less Than 18 Years With Invasive Aspergillosis

Short Title: Posaconazole (MK-5592) IV and oral in children with invasive aspergillosis

Acronym: Not applicable

Hypotheses, Objectives, and Endpoints:

There are no hypotheses for this trial. The following objectives and endpoints will be evaluated in male and female pediatric participants aged 2 to <18 years with invasive aspergillosis (IA):

Primary Objectives	Primary Endpoints
- Objective: To evaluate the safety of POS (IV and oral formulations overall)	- Treatment-related AEs
Secondary Objectives	Secondary Endpoints
- Objective: To evaluate the efficacy of POS (IV and oral formulations overall) in participants with possible, probable, or proven IA	- Global clinical response (partial or complete response)
- Objective: To evaluate relapse in participants with possible, probable, or proven IA who have completed treatment with POS (IV and oral formulations overall) and achieved favorable global clinical response (complete or partial)	- Relapse of IA, defined as the re-emergence of clinical, radiographic, or other relevant abnormalities indicating IA
- Objective: To characterize the PK of POS overall and by formulation	- Key PK parameters, consisting of Cavg, Cmin, Cmax, AUC, and Tmax, using sparse plasma concentration sampling (steady state trough and peak)<br>- Analysis of exposure-response (efficacy and safety) relationships

- Objective: To summarize the palatability and acceptability of POS powder for suspension (PFS) formulation	- Participants' categorical perception of the taste of the PFS formulation

Overall Design:

Study Phase	Phase 2
Primary Purpose	Treatment
Indication	Invasive aspergillosis
Population	Male and female participants aged ≥2 to <18 years with invasive aspergillosis
Study Type	Interventional
Intervention Model	Single Group<br>This is a multi-site study.
Type of Control	No treatment control
Study Blinding	Unblinded Open-label
Masking	No Masking
Estimated Duration of Study	The Sponsor estimates that the study will require approximately 3 years from the time the first participant (or their legally acceptable representative) provides documented informed consent/assent until the last participant's last study-related contact.

Number of Participants:

Approximately 30 participants will be enrolled in the trial to achieve at least 15 participants in the Full Analysis Set (FAS) population (defined in Section 9.5.1) as characterized below.

Participants will be enrolled into the following age cohorts, which will enroll simultaneously:

- Age Cohort 1 (aged 2 to <12 years): approximately 20 participants enrolled to achieve at least 10 in the FAS population who transition to oral therapy (PFS or tablet) for at least 7 days after the end of IV treatment with POS.

- Age Cohort 2 (aged 12 years to <18 years): approximately 10 participants enrolled to achieve at least 5 in the FAS population who transition to oral therapy (PFS or tablet) for at least 7 days after the end of IV treatment with POS.

Intervention Groups and Duration:

<table>
<tr><td>Intervention Groups</td><td>
<table>
<tr><th>Treatment Name</th><th>Drug</th><th>Dose Strength</th><th>Dose Frequency</th><th>Route of Admin-istration</th><th>Regimen/ Treatment Period[b]</th><th>Use</th></tr>
<tr><td>Posaconazole</td><td>Posaconazole IV [a]</td><td>18 mg/mL</td><td>QD, except BID on Day 1</td><td>IV infusion</td><td>Day 1: BID 6 mg/kg

Day 2 through end of IV dosing:
QD 6 mg/kg</td><td rowspan="3">Test Product</td></tr>
<tr><td>Posaconazole</td><td>If weight ≤40 kg: Posaconazole PFS</td><td>30 mg/mL</td><td>QD</td><td>Oral</td><td>Days 8 – 84: weight-band dosing, see Table 1.</td></tr>
<tr><td>Posaconazole</td><td>If weight >40 kg: Posaconazole Tablet</td><td>100 mg</td><td>QD</td><td>Oral</td><td>Days 8 – 84: 300 mg</td></tr>
<tr><td colspan="7">Abbreviations: BID = twice daily; IV = intravenous; PFS = powder for delayed-release oral suspension; QD = once daily.
a: There is a maximum dose of 300 mg per administration; on Day 1 only, dosing frequency is BID, with up to 300 mg IV in each dose.
b: The posaconazole IV formulation is to be administered for a minimum of 7 days; oral formulations (PFS or tablet) may be administered beginning on Day 8, as clinically indicated.</td></tr>
</table>

Other current or former name(s) or alias(es) for study intervention(s) are as follows: MK-5592; Noxafil; SCH 56592.</td></tr>
<tr><td>Total Number</td><td>There will be a single intervention arm.</td></tr>
<tr><td>Duration of Participation</td><td>Each participant will participate in the trial for up to approximately 18 weeks (128 days) from the time the participant or legally acceptable representative provides documented informed consent/assent through the final contact. After a screening phase of up to 2 weeks (14 days), each participant will be followed for the trial duration of approximately 114 days. All participants are to be followed for the entire trial period regardless of the duration of study therapy, unless consent/assent has been withdrawn. Participants are to receive assigned study treatment for 6 to 12 weeks (42 to 84 days); actual treatment duration for each participant will be based on the investigator’s clinical judgment.</td></tr>
</table>

Study Governance Committees:

Steering Committee	No
Executive Oversight Committee	Yes
Data Monitoring Committee	Yes
Clinical Adjudication Committee	No
Other Oversight Committee	No
Study governance considerations are outlined in Appendix 1.	

Study Accepts Healthy Volunteers: No

A list of abbreviations used in this document can be found in Appendix 8.

1.2 Schema

The trial design is depicted in Figure 1.

Figure 1 Trial Design

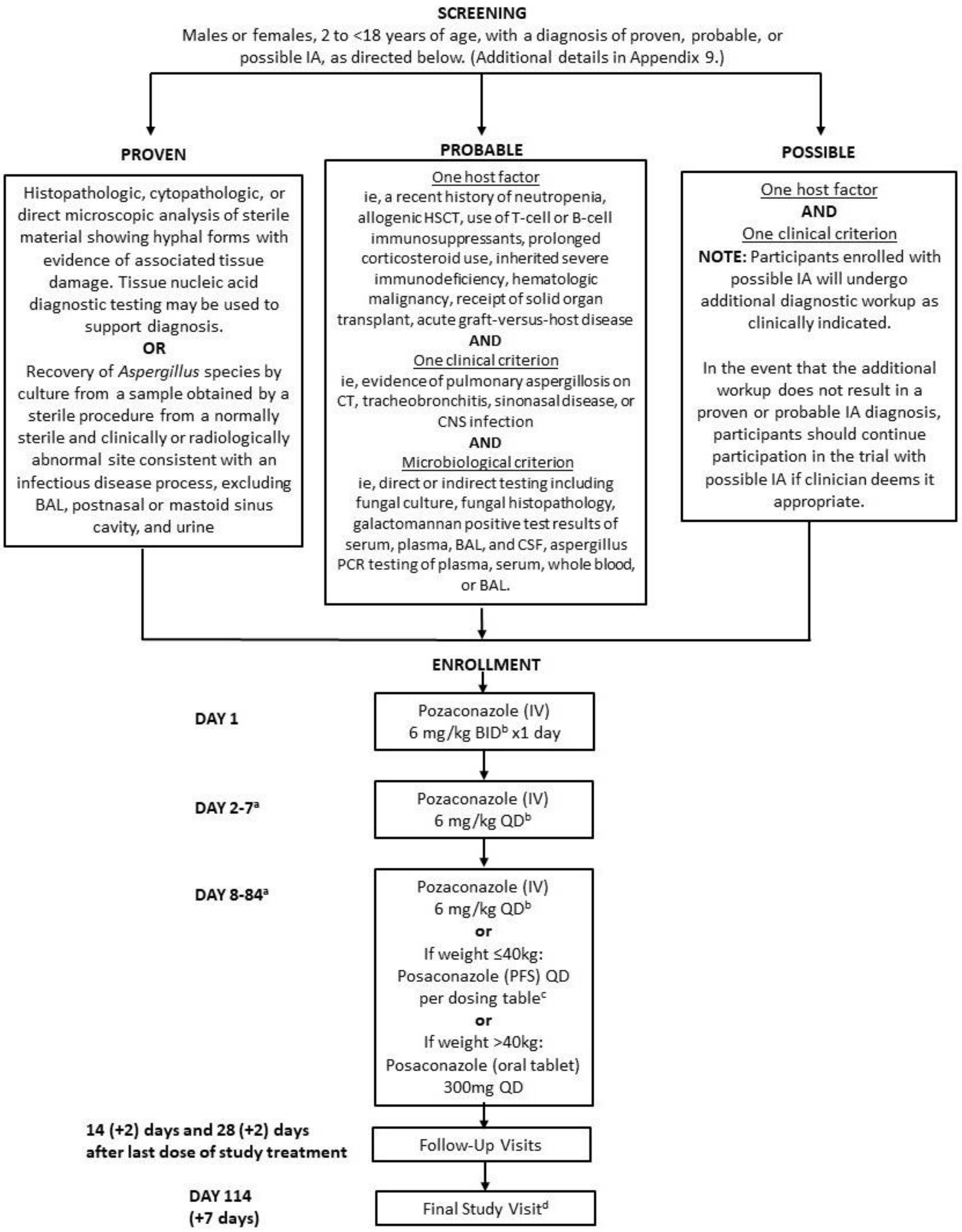

a. All participants will initiate treatment with IV study treatment and may transition to oral study treatment after a minimum of 7 days on IV study treatment and as clinically indicated, as described in Section 4.3. The planned duration of study treatment is 6 to 12 weeks (42 to 84 days); the actual treatment duration for each participant will be based on clinical judgment of the investigator.
b. To a maximum dose of 300mg per administration.
c. Refer to Table 1 in Section 4.3.1 for weight-based dosing of the formulation for participants weighing ≤40kg.
d. Mortality will be assessed through the Day 114 visit for all participants

1.3 Schedule of Activities (SoA)

**Study Period**	**Screening**	**Treatment**									**Follow-Up**			**Unsch**	**Notes**
**Visit Number/Title**	**1 Screening**	**2 Baseline**	**3**	**4**	**5**	**6**	**7**	**8**	**9**	**EOT[a]**	**10**	**11**	**12**	**Unsch**	a. Participants who discontinue study therapy before Wk 12 should have an EOT Visit within 3 days after EOT.
**Scheduled Day/ Week and Window**	**Days -14 to -1**	**Day 1**	**Day 3 (±1 day)**	**Wk 1 (Days 7-9)**	**Wk 2 (Days 12-18)**	**Wk 4 (Days 25-33)**	**Wk 6 (Days 39-47)**	**Wk 9 (Days 60-68)**	**Wk 12 (Days 81-89)**	**EOT**	**14 (+2) days after last dose**	**28 (+2) days after last dose**	**Day 114 (+7 days)**	**Unsch[b]**	b. Procedures at unscheduled visits to be performed as clinically appropriate.
**Administrative Procedures**															
Assign screening number	X														
Obtain informed consent/assent	X														
Issue participant identification card	X	X													See Section 8.1.3
Review inclus./ exclus. criteria	X	X													
Medical history	X														
Prior/concom. medication review	X	X	X	X	X	X	X	X	X	X	X	X		X	
**Dosing Procedures**															
Stratification and treatment allocation no. assigned by IRT		X													

Study Period	Screening	Treatment									Follow-Up			Unsch	Notes
**Visit Number/Title**	**1 Screening**	**2 Baseline**	**3**	**4**	**5**	**6**	**7**	**8**	**9**	**EOT[a]**	**10**	**11**	**12**	**Unsch**	a. Participants who discontinue study therapy before Wk 12 should have an EOT Visit within 3 days after EOT.
**Scheduled Day/ Week and Window**	**Days -14 to -1**	**Day 1**	**Day 3 (±1 day)**	**Wk 1 (Days 7-9)**	**Wk 2 (Days 12-18)**	**Wk 4 (Days 25-33)**	**Wk 6 (Days 39-47)**	**Wk 9 (Days 60-68)**	**Wk 12 (Days 81-89)**	**EOT**	**14 (+2) days after last dose**	**28 (+2) days after last dose**	**Day 114 (+7 days)**	**Unsch[b]**	b. Procedures at unscheduled visits to be performed as clinically appropriate.
POS (MK-5592) administration		X	X	X	X	X	X	X	X					X	Daily from Day 1 through Week 12. IV dose on Day 1 BID; subsequent doses QD.
Collect unused medications					X	X	X	X	X	X				X	For participants receiving oral formulations only.
Dosing compliance assessment					X	X	X	X	X	X				X	
**Safety and Tolerability Procedures**															
Vital signs (heart rate, blood pressure, respiratory rate, temperature)	X	X	X	X	X	X	X	X	X	X	X	X		X	While on IV treatment, vital signs to be collected daily. While on oral treatment, vital signs to be collected at each visit.

**Study Period**	**Screening**	**Treatment**									**Follow-Up**			**Unsch**	**Notes**
**Visit Number/Title**	**1 Screening**	**2 Baseline**	**3**	**4**	**5**	**6**	**7**	**8**	**9**	**EOT[a]**	**10**	**11**	**12**	**Unsch**	a. Participants who discontinue study therapy before Wk 12 should have an EOT Visit within 3 days after EOT.
**Scheduled Day/ Week and Window**	**Days -14 to -1**	**Day 1**	**Day 3 (±1 day)**	**Wk 1 (Days 7-9)**	**Wk 2 (Days 12-18)**	**Wk 4 (Days 25-33)**	**Wk 6 (Days 39-47)**	**Wk 9 (Days 60-68)**	**Wk 12 (Days 81-89)**	**EOT**	**14 (+2) days after last dose**	**28 (+2) days after last dose**	**Day 114 (+7 days)**	**Unsch[b]**	b. Procedures at unscheduled visits to be performed as clinically appropriate.
Urine or serum pregnancy test (Females of childbearing potential only)	X	X				X		X	X	X	X	X			If pregnancy test perform at Screening and Day 1 visit occurs >24 hours later, pregnancy test to be repeated at Day 1 visit.
Full physical examination	X														
Directed physical examination		X		X	X	X	X	X	X	X	X	X		X	
Body weight (kg)	X	X		X	X	X	X	X							After Day 1, weight to be measured when oral treatment is initiated (within 3 days before initiating oral treatment).
Height	X														
AE/SAE Review	X	X	X	X	X	X	X	X	X	X	X	X		X	

**Study Period**	**Screening**	**Treatment**									**Follow-Up**			**Unsch**	**Notes**
**Visit Number/Title**	**1 Screening**	**2 Baseline**	**3**	**4**	**5**	**6**	**7**	**8**	**9**	**EOT[a]**	**10**	**11**	**12**	**Unsch**	a. Participants who discontinue study therapy before Wk 12 should have an EOT Visit within 3 days after EOT.
**Scheduled Day/ Week and Window**	**Days -14 to -1**	**Day 1**	**Day 3 (±1 day)**	**Wk 1 (Days 7-9)**	**Wk 2 (Days 12-18)**	**Wk 4 (Days 25-33)**	**Wk 6 (Days 39-47)**	**Wk 9 (Days 60-68)**	**Wk 12 (Days 81-89)**	**EOT**	**14 (+2) days after last dose**	**28 (+2) days after last dose**	**Day 114 (+7 days)**	**Unsch[b]**	b. Procedures at unscheduled visits to be performed as clinically appropriate.
12-lead ECG	X	X	X	X					X	X				X	Day 1: predose. While on POS IV, ECG to be obtained after completion of infusion. While on oral POS, ECG can be performed without regard to timing of study treatment. See Section 10.7, Appendix 7 for country-specific ECG requirements.
Hematology and serum chemistry	X	X	X	X	X	X	X	X	X	X	X	X		X	At Day 1, repeat hematology and chemistry only if >2 days since screening laboratory assessments.

Study Period	Screening	Treatment									Follow-Up			Unsch	Notes
**Visit Number/Title**	**1 Screening**	**2 Baseline**	**3**	**4**	**5**	**6**	**7**	**8**	**9**	**EOT[a]**	**10**	**11**	**12**	**Unsch**	a. Participants who discontinue study therapy before Wk 12 should have an EOT Visit within 3 days after EOT.
**Scheduled Day/ Week and Window**	**Days -14 to -1**	**Day 1**	**Day 3 (±1 day)**	**Wk 1 (Days 7-9)**	**Wk 2 (Days 12-18)**	**Wk 4 (Days 25-33)**	**Wk 6 (Days 39-47)**	**Wk 9 (Days 60-68)**	**Wk 12 (Days 81-89)**	**EOT**	**14 (+2) days after last dose**	**28 (+2) days after last dose**	**Day 114 (+7 days)**	**Unsch[b]**	b. Procedures at unscheduled visits to be performed as clinically appropriate.
PFS: Assessment of palatability and acceptability				X	X	X	X	X	X	X					For participants receiving PFS formulation, to be performed only on first and last days of PFS treatment.
**Efficacy Procedures**															
Assess global clinical response							X		X	X					
Diagnostic imaging	X[c]	X[d]			X[d]	X[d]	X[d]	X[d]	X[d]	X[c]	X[d]	X[d]		X	c Required for all sites of infection only at screening and EOT. d. At other visits, if clinical condition changes, additional imaging can be performed at the clinician's discretion.

Study Period	Screening	Treatment									Follow-Up			Unsch	Notes
**Visit Number/Title**	**1 Screening**	**2 Baseline**	**3**	**4**	**5**	**6**	**7**	**8**	**9**	**EOT[a]**	**10**	**11**	**12**	**Unsch**	a. Participants who discontinue study therapy before Wk 12 should have an EOT Visit within 3 days after EOT.
**Scheduled Day/ Week and Window**	**Days -14 to -1**	**Day 1**	**Day 3 (±1 day)**	**Wk 1 (Days 7-9)**	**Wk 2 (Days 12-18)**	**Wk 4 (Days 25-33)**	**Wk 6 (Days 39-47)**	**Wk 9 (Days 60-68)**	**Wk 12 (Days 81-89)**	**EOT**	**14 (+2) days after last dose**	**28 (+2) days after last dose**	**Day 114 (+7 days)**	**Unsch[b]**	b. Procedures at unscheduled visits to be performed as clinically appropriate.
IFI assessment		X	X	X	X	X	X	X	X	X	X	X			MSG/EORTC criteria; details in Appendix 9.
Assessment of relapse											X	X			Relapse to be assessed only in participants who have achieved a favorable clinical response and have completed study treatment.
Mortality assessment			X	X	X	X	X	X	X	X	X	X	X	X	Mortality to be assessed through Day 114 visit for all participants.

Study Period	Screening	Treatment									Follow-Up			Unsch	Notes
**Visit Number/Title**	**1 Screening**	**2 Baseline**	**3**	**4**	**5**	**6**	**7**	**8**	**9**	**EOT[a]**	**10**	**11**	**12**	**Unsch**	a. Participants who discontinue study therapy before Wk 12 should have an EOT Visit within 3 days after EOT.
**Scheduled Day/ Week and Window**	**Days -14 to -1**	**Day 1**	**Day 3 (±1 day)**	**Wk 1 (Days 7-9)**	**Wk 2 (Days 12-18)**	**Wk 4 (Days 25-33)**	**Wk 6 (Days 39-47)**	**Wk 9 (Days 60-68)**	**Wk 12 (Days 81-89)**	**EOT**	**14 (+2) days after last dose**	**28 (+2) days after last dose**	**Day 114 (+7 days)**	**Unsch[b]**	b. Procedures at unscheduled visits to be performed as clinically appropriate.
Sample for *Aspergillus* galactomannan EIA	X	X	X	X	X	X	X	X	X	X	X	X		X	If collected within 7 days of Day 1, can be used for screening purposes. Collect samples as clinically indicated.
Mycology testing (direct and indirect test)	X	X		X	X	X	X	X	X	X				X	Required at Screening, Wk 6 & 12, and EOT visits, unless clinically inappropriate. Additional optional testing, including *Aspergillus* PCR, will be performed as clinically indicated. See Appendix 9.

Study Period	Screening	Treatment									Follow-Up			Unsch	Notes
**Visit Number/Title**	**1 Screening**	**2 Baseline**	**3**	**4**	**5**	**6**	**7**	**8**	**9**	**EOT[a]**	**10**	**11**	**12**	**Unsch**	a. Participants who discontinue study therapy before Wk 12 should have an EOT Visit within 3 days after EOT.
**Scheduled Day/ Week and Window**	**Days -14 to -1**	**Day 1**	**Day 3 (±1 day)**	**Wk 1 (Days 7-9)**	**Wk 2 (Days 12-18)**	**Wk 4 (Days 25-33)**	**Wk 6 (Days 39-47)**	**Wk 9 (Days 60-68)**	**Wk 12 (Days 81-89)**	**EOT**	**14 (+2) days after last dose**	**28 (+2) days after last dose**	**Day 114 (+7 days)**	**Unsch[b]**	b. Procedures at unscheduled visits to be performed as clinically appropriate.
**PK/Biomarkers**															
Plasma PK assessment		X		X	X	X	X	X	X	X				X	
Abbreviations: AE = adverse event; ECG = electrocardiogram; BAL = broncho-alveolar lavage; EIA = enzyme immunoassay; EOT = end of treatment; IA = invasive aspergillosis; IFI = invasive fungal infection; IRT = interactive response technology; IV = intravenous; PFS = powder for oral suspension; PK = pharmacokinetics; POS = posaconazole (MK-5592); SAE = serious adverse event; Wk = week.															

2 INTRODUCTION

2.1 Study Rationale

As the number of immunocompromised patients, both adult and pediatric, continues to increase, IFIs, including IA, play a significant role in the morbidity and mortality seen in this population. There continues to be a need for the development of effective treatment options, particularly in the pediatric population for whom there are limited clinical study data detailing the treatment of IA. Recent advances in the adult population include the approval of POS in the US, European Union, and other countries for the primary treatment of IA [E.U. Summary of Product Characteristics 2022] [U.S. Prescribing Information 2022]. These approvals were based upon the completion of a prospective, randomized, double-blind study that showed the safety and efficacy of POS IV and tablet for the treatment of IA in adolescents and adults [Maertens, J. A., et al 2021].

Invasive aspergillosis occurs in pediatric (and adult) populations that are immunocompromised due to various pathologic and other factors, eg, malignancy, solid organ transplant or HSCT, primary immunodeficiency. The incidence rates of IA in high -risk patients vary by disease type and, in some instances, by geography and the use of prophylactic agents.

In children, IA is associated with significant increases in hospital length of stay and high overall in hospital mortality rates. Analyses of data from several major US hospitals showed an overall incidence of IA among hospitalized immunocompromised children of 437/100,000 (0.4%) [Pana, Z. D., et al 2017], and mortality rates in children with IA between 18% and 37% [Burgos, A., et al 2008].

Despite this unmet medical need, IA infections in immunocompromised pediatric patients have been understudied. To date, pediatric IA treatment options have been extrapolated from IA adult studies and treatment options [Burgos, A., et al 2008]. Current guidelines [Patterson, T. F., et al 2016] for the management of IA in adult patients recommend voriconazole (VOR) for the primary treatment of invasive pulmonary aspergillosis, with posaconazole, isavuconazole, or lipid formulations of amphotericin B as alternative primary therapies[Douglas, A. P., et al 2021]. However, guidelines are limited given the paucity of prospective study data in the pediatric population.

A small number of studies have provided data regarding recent treatment choices for pediatric patients diagnosed with IA. A 2017 trial of VOR in pediatric participants with IA demonstrated that safety and efficacy outcomes were consistent with previous findings in adult patients [Martin, J. M., et al 2017]. In this 2017 trial, 31 children (14 with proven or probable infection) were enrolled and treated for IA for 6 to 12 weeks. After 6 to 12 weeks of VOR therapy, the global clinical response was 64%. AEs were common, with 30/31 (97%) participants reporting one or more AEs, and 16/31 (52%) of the AEs were considered related to study therapy. In this trial, an association between hepatic AEs and higher VOR exposures was noted, although due to the small sample size, data interpretation was limited. Overall, 48% of participants treated with VOR discontinued therapy early. These results indicate the need to identify alternate therapies for IA that are more efficacious and/or better tolerated.

POS is a highly active triazole with potential advantages in terms of tolerability and drug interactions. POS (IV, PFS, and tablet formulations) has been recently approved for use in children 2 to <18 years of age for the salvage treatment and the prophylaxis of IFIs. This approval was based on data from a prospective PK and safety study that evaluated POS IV and PFS in children 2 to 17 years of age who were at high risk of an invasive fungal infection [Groll, A. H., et al 2020].

The use of newer formulations of POS (PFS, tablet, and IV) provides higher POS concentrations in pediatric patients than the oral suspension formulation and supports the use of the IV formulation in pediatric patients who are unable to tolerate oral dosing. As the newer IV, tablet, and PFS formulations of POS achieve higher POS exposures, the newer formulations have the potential for improved clinical effectiveness.

As immunocompromised pediatric patients are at risk for IA, it is appropriate to study the efficacy and safety of POS IV and oral formulations (PFS and tablets) for the primary treatment of IA in children. This trial evaluates safety, efficacy, and PK of POS IV and oral formulations (PFS and tablets) in pediatric participants 2 to <18 years of age with IA.

2.2 Background

Refer to the IB and approved product labeling for detailed background information on posaconazole (MK-5592, POS).

2.2.1 Pharmaceutical and Therapeutic Background

POS is a broad-spectrum triazole antifungal compound approved for the prophylaxis and salvage treatment of IFIs in pediatric patients 2 to <18 years of age. POS is also approved for the primary treatment of IA as well as prophylaxis and salvage treatment of IFIs in adolescents and adults. The approved labeling of indications and patient populations vary by country. Currently approved POS formulations include an IV solution and 3 oral formulations (a pre-mixed liquid suspension, a powder for suspension to be mixed in a suspending vehicle, and a delayed-release tablet). The powder for suspension and the delayed-release gastric tablet formulations overcome limitations in the oral bioavailability of the oral suspension, allowing for once-daily dosing and, as such, are preferred oral formulations. The oral PFS formulation consists of the same delayed-release compound as the tablet in a formulation that allows for more flexible weight-based dosing. A Phase 1B trial [MK-5592-097 (P097)] has provided PK and safety data for use of the IV and PFS formulations, when given prophylactically to children at risk of IFI, that supported the approval of these formulations in patients 2 through 17 years of age.

2.2.2 Preclinical and Clinical Studies

Refer to the IB and approved POS labeling for detailed information on prior preclinical and clinical studies conducted with POS.

2.2.3 Ongoing Clinical Studies

There is 1 study ongoing, in addition to the current study, that is evaluating the use of POS in the pediatric population. In addition to this protocol, there is also another ongoing Phase 2 clinical study of POS evaluation in the treatment of invasive fungal infections in pediatric patients 0 to 2 years of age (P127).

2.3 Benefit/Risk Assessment

It cannot be guaranteed that participants in clinical studies will directly benefit from treatment during participation, as clinical studies are designed to provide information about the safety and effectiveness of an investigational medicine.

POS has been shown to be of benefit in the treatment of IA and other IFIs in controlled as well as uncontrolled studies and in salvage therapy studies using the IV, tablet, and oral suspension formulation in adults, and the IV and PFS formulation in pediatric participants [Walsh, T. J., et al 2007] [Lehrnbecher, T., et al 2010] [Maertens, J. A., et al 2021] [Groll, A. H., et al 2020]. In addition, literature reports of POS IV and tablet have indicated the benefit of the POS when used to treat mold infections, including IA, in high-risk patients [Conant, M. M., et al 2015] [Jeong, W., et al 2016]. The most common toxicities that have been reported with POS include gastrointestinal events (diarrhea, nausea, and vomiting), fever, headache, and cough. The following risks have been identified in patients treated with POS per product labeling: hypersensitivity, hepatic toxicity, QTc prolongation, and drug interactions. Refer to current POS (NOXAFIL™) product labeling for additional information. The safety and efficacy of POS in children <2 years of age has not been established. Current product labeling indicates approval in children 2 years of age and older and adults.

Additional details regarding specific benefits and risks for participants participating in this clinical trial may be found in the accompanying IB and informed consent documents.

3 HYPOTHESES, OBJECTIVES, AND ENDPOINTS

There are no hypotheses for this trial. The following objectives and endpoints will be evaluated in male and female pediatric participants aged 2 to <18 years with invasive aspergillosis (IA): time points at which each of the endpoints will be evaluated are presented in Section 9.1.

Objectives	Endpoints
Primary	
• Objective: To evaluate the safety of POS (IV and oral formulations overall)	• Treatment-related AEs
Secondary	
• Objective: To evaluate the efficacy of POS (IV and oral formulations overall) in participants with possible, probable, or proven IA	• Global clinical response (partial or complete response)
• Objective: To evaluate relapse in participants with possible, probable, or proven IA who have completed treatment with POS (IV and oral formulations overall) and achieved favorable global clinical response (complete or partial)	• Relapse of IA, defined as the re-emergence of clinical, radiographic, or other relevant abnormalities indicating IA
• Objective: To characterize the PK of POS overall and by formulation	• Key PK parameters, consisting of Cavg, Cmin, Cmax, AUC, and Tmax, using sparse plasma concentration sampling (steady-state trough and peak)<br>• Analysis of exposure-response (efficacy and safety) relationships
• Objective: To summarize the palatability and acceptability of POS powder for suspension (PFS) formulation	• Participants' categorical perception of the taste of the PFS formulation
Tertiary/Exploratory	
• Objective: To evaluate all-cause mortality in participants treated with POS (IV and oral formulations overall)	• Deaths

The timing of all assessments, including those not directly related to endpoints, is described elsewhere in the protocol (Sections 1.3, 8.2, 8.3, 8.4, 8.6, 9.1 and 9.4).

4 STUDY DESIGN

4.1 Overall Design

This is a nonrandomized, open-label, noncomparative, Phase 2, multi-site trial of POS IV and oral formulations in pediatric participants aged 2 to <18 years with IA, as defined by 2020 European Organization for Research and Treatment of Cancer/Mycoses Study Group (EORTC/MSG) consensus definitions [Donnelly, J. P., et al 2020]. Individuals who meet the criteria for possible, probable, or proven IA will be enrolled.

After a screening period of up to 14 days, participants will be allocated to receive open-label POS study treatment. All participants will initiate treatment with the POS IV formulation. After a minimum of 7 days on POS IV, and when the participant is considered clinically stable and able to take oral study treatment, per investigator judgment, participants may transition to oral study treatment with either the POS tablet formulation or the POS PFS formulation, based on their weight. The initial transition to POS oral formulation (either PFS or tablet) should occur no later than the Week 9 visit and must occur under medical supervision at the study site. After transitioning to one of the oral POS formulations, additional transitions between oral and IV formulations are permitted as clinically indicated.

The planned total duration of study treatment across all formulations is 6 to 12 weeks (42 to 84 days); the actual treatment duration for each participant will be based on the investigator's clinical judgment. Participants should complete all study visits regardless of when they complete study treatment. Doses and additional details of study treatment are provided in Section 6.1.

Safety, efficacy, and PK assessments will be conducted throughout the treatment period, and safety and efficacy assessments at the follow-up visits, 14 (+2) and 28 (+2) days after the last dose of study treatment and at Day 114 (+7 days), as specified in Section 1.3. All participants will be followed for survival through Day 114. The trial's objectives are stated in Section 3 and include safety (primary objective) as well as efficacy, PK, and palatability and acceptability (secondary objectives).

Participants will participate in the trial for up to approximately 128 days: up to 14 days of screening, up to 84 days of treatment, and approximately 30 days of follow-up after the 84-day planned treatment period.

Specific procedures to be performed during the study, as well as their prescribed times and associated visit windows, are outlined in the SoA in Section 1.3. Details of each procedure are provided in Section 8.

Approximately 30 participants will be enrolled to achieve at least 15 participants as characterized below in the FAS population (Section 9.5.1). Participants will be enrolled into the following age cohorts, which will enroll simultaneously:

- Age Cohort 1 (2 years to <12 years of age): approximately 20 participants enrolled to achieve at least 10 in the FAS population who transition to oral therapy (PFS or tablet) for at least 7 days after the end of IV treatment with POS.
- Age Cohort 2 (12 years to <18 years of age): approximately 10 participants enrolled to achieve at least 5 in the FAS population who transition to oral therapy (PFS or tablet) for at least 7 days after the end of IV treatment with POS.

For ongoing safety evaluations and to ensure safe study conduct, an independent, external DMC will be established. Specific details regarding the DMC will be described in the DMC charter.

4.2 Scientific Rationale for Study Design

As immunocompromised pediatric patients (aged <18 years of age) are at high risk for IA, newer treatments are needed for pediatric patient populations. POS IV and tablet formulations have been studied in adolescents and adults for the primary treatment of IA and have demonstrated efficacy and safety for this indication. Thus, it is reasonable to further study the safety and efficacy of POS IV, tablet, and PFS in the primary treatment of IA in the pediatric population (see Section 2.2.3).

4.2.1 Rationale for Endpoints

4.2.1.1 Efficacy Endpoints

Efficacy is not a primary endpoint in this study. The efficacy endpoint (secondary) is the proportion of participants with a diagnosis of possible, probable, or proven IA and a favorable global clinical response. This endpoint will be assessed by the investigator using the published definitions for defining responses to therapy and study outcomes in clinical trials of invasive fungal diseases [Segal, B. H., et al 2008] and as presented in Section 8.2.1. The assessment of the efficacy endpoint in pediatric participants with a classification of possible, probable, or proven IA is similar to the efficacy population analyzed in the adult study (MK-5592-069 P069) of IA. Additional efficacy endpoints are:

- An assessment of relapse of IA in participants with possible, probable, or proven IA who have achieved a favorable global clinical response after completion of study treatment (secondary endpoint, Section 9.4.1.1).
- An evaluation of all-cause mortality at Study Day 42 (exploratory endpoint, Section 9.4.1.3). All-cause mortality at Study Day 42 as an efficacy endpoint has been considered an accepted endpoint for recent clinical studies of IA in adults and adolescents[Martin, J. M., et al 2017]. All-cause mortality will also be evaluated at Study Day 114.

4.2.1.2 Safety Endpoints

The safety of POS in this pediatric population will be primarily assessed by the evaluation of treatment-related AEs. Other assessments may include evaluation of all AEs (regardless of causality), as well as laboratory and ECG safety parameters.

4.2.1.3 Pharmacokinetic Endpoints

Key PK parameters will be calculated from sparse plasma concentration data based on previously developed population PK models for each formulation in pediatric participants.

Exposure/response assessments will also be conducted, including an exploration of the relationship between PK parameters and measures of efficacy and safety.

4.2.1.4 Additional Endpoints

Palatability and acceptability parameters for the POS PFS formulation will be assessed in all participants who receive this formulation (refer to Section 8.3.5 for further details of the palatability assessment).

4.2.2 Rationale for the Use of Comparator/Placebo

This study is designed to obtain efficacy and safety information regarding the use of POS IV and oral formulations as primary treatment of IA in children ≥2 and <18 years of age. To obtain as much experience as possible with POS treatment in this trial, given the limited participant population, no comparator will be assessed. POS has previously been demonstrated to be efficacious in the primary treatment of IA in a double-blind comparative study of POS IV and tablet compared with VOR IV and oral formulations when given to adults and adolescents [Maertens, J. A., et al 2021].

Given the high rate of mortality of IA among untreated patients, the use of a placebo treatment arm has not been considered.

4.3 Justification for Dose

4.3.1 Starting Dose for This Study

All randomized participants will begin study dosing with the IV formulation of POS. The dose of 6 mg/kg/day for the IV formulation was chosen for this study based on the results of the Phase 1B PK and safety study of POS in children and adolescents (P097) as well as the approved dosing of POS IV for children 2 years of age and older. Participants will receive POS IV at a dose of 6 mg/kg/day BID on Day 1 and QD thereafter.

After at least 7 days and when clinically appropriate, participants can be switched to an oral formulation. Participants weighing ≤40 kg will transition to the PFS formulation. The dosing for the PFS formulation, shown in Table 1, reflects the approved pediatric weight-based dosing of this formulation and reflects the approved labeled dosing of this formulation. Weight-band dosing is expected to provide POS exposures similar to that previously

specified in the protocol before implementation of this amendment. Participants weighing >40 kg will transition to the tablet at a dose of 300 mg. The choice of 40 kg as the weight to transition to a 300-mg tablet dose aligns with the pediatric dosing of the tablet in approved product labeling for POS tablet. The dosing of the tablet also reflects the approved dosing of POS tablet as shown in product labelling for the treatment of IA.

Table 1 Dosing of the PFS

Weight (kg)	PFS Dose (volume)
10-<12 kg	90 mg (3 mL)
12-<17 kg	120 mg (4 mL)
17-<21 kg	150 mg (5 mL)
21-<26 kg	180 mg (6 mL)
26-<36 kg	210 mg (7 mL)
36-40 kg	240 mg (8 mL)
PFS= powder for delayed release oral suspension	

4.3.2 Maximum Dose/Exposure for This Study

For a given participant, the maximum planned dose of POS is 300 mg per administration (administered BID on Day 1 and QD on subsequent days for a maximum of 84 days). Participants who weigh >40 kg will receive the maximum planned dose of 300 mg. The maximum total daily dosing established for this study reflects the approved dose for POS solid oral tablet and POS IV solution formulations, which is 300 mg BID on Day 1 followed by 300 mg QD.

4.3.3 Rationale for Dose Interval and Study Design

In this study, all participants will initiate treatment with IV POS and may transition to oral POS after a minimum of 7 days on IV study treatment and as clinically indicated. Participants who weigh >40 kg will transition to the tablet as the oral formulation; participants who weigh ≤40 kg and are able to transition to an oral formulation will transition to PFS. Both IV and oral treatments have been used to treat IA and other IFIs in controlled studies of IA as well as in uncontrolled studies and in salvage therapy studies [Walsh, T. J., et al 2007] [Lehrnbecher, T., et al 2010] [Conant, M. M., et al 2015] [Jeong, W., et al 2016]. Consistent with the POS (NOXAFIL) label, POS is administered twice on the first day of study treatment and then QD for the duration of treatment.

The planned duration of study treatment is 6 to 12 weeks (42 to 84 days); this aligns with current recommendations for the treatment of IA, which call for a minimum duration of treatment of 6 to 12 weeks [Patterson, T. F., et al 2016].

In this study, participants with possible IA will begin study treatment before their diagnosis of IA is confirmed. Early initiation of antifungal treatment while the definitive diagnostic evaluation is in progress is recommended by current guidelines [Patterson, T. F., et al 2016].

4.4 Beginning and End of Study Definition

The overall study begins when the first participant (or their legally acceptable representative) provides documented informed consent/assent. The overall study ends when the last participant completes the last study-related contact, withdraws consent, or is lost to follow-up (ie, the participant is unable to be contacted by the investigator).

For the purposes of analysis and reporting, the overall study ends when the Sponsor receives the last laboratory result or at the time of final contact with the last participant, whichever comes last. If the study includes countries in the EEA, the local start of the study in the EEA is defined as First Site Ready in any Member State.

4.4.1 Clinical Criteria for Early Study Termination

The clinical study may be terminated early if the extent (incidence and/or severity) of emerging effects/clinical endpoints is such that the risk/benefit ratio to the study population as a whole is unacceptable. In addition, further recruitment in the study or at (a) particular study site(s) may be stopped due to insufficient compliance with the protocol, GCP and/or other applicable regulatory requirements, procedure-related problems or the number of discontinuations for administrative reasons is too high.

5 STUDY POPULATION

As stated in the Code of Conduct for Clinical Trials (Appendix 1), our studies include people of varying age, race, ethnicity, and sex. The collection and use of these demographic data are to follow all local laws and guidelines in keeping with the needs for participant confidentiality while supporting the study of the disease, its related factors, and the IMP under investigation.

Male/female participants with a diagnosis of possible, probable, or proven IA aged 2 to <18 years will be enrolled in this study.

Prospective approval of protocol deviations to recruitment and enrollment criteria, also known as protocol waivers or exemptions, is not permitted.

5.1 Inclusion Criteria

A participant will be eligible for inclusion in the study if the participant:

Type of Participant and Disease Characteristics

1. Has a diagnosis of possible, probable, or proven IA per 2020 European Organization for Research and Treatment of Cancer/Mycoses Study Group (EORTC/MSG) disease definitions (Appendix 9).

2. If enrolled with a possible or probable IA diagnosis, has one or more of the following risks as per 2020 EORTC/MSG disease definitions (Appendix 9).

   - Recent history of neutropenia (<0.5 × 10^9 neutrophils/L [<500 neutrophils/mm^3]) (within 30 days before screening).
   - Receipt of an allogeneic HSCT.
   - Receipt of a solid organ transplant.
   - Hematologic malignancy currently under treatment or has been treated in the recent past.
   - Treatment with other recognized T-cell immunosuppressants, such as calcineurin inhibitors, TNF-α blockers, specific monoclonal antibodies (such as alemtuzumab), or nucleoside analogues during the past 90 days.
   - Prolonged use corticosteroids for ≥3 weeks in the past 60 days (average minimum dose of ≥0.3 mg/kg/day of prednisone equivalent).
   - Congenital or inherited severe immunodeficiency (including, but not limited to chronic granulomatous disease, STAT 3 deficiency, CARD9 deficiency, STAT-1 gain of function, or severe combined immunodeficiency).
   - Treatment with recognized B-cell immunosuppressants, such as Bruton's tyrosine kinase inhibitors, eg, ibrutinib.
   - Acute graft-versus-host disease grade III or IV involving the gut, lungs, or liver that is refractory to first-line treatment with steroids.

3. If enrolled with a possible or probable IA diagnosis, meets mycologic and clinical criteria as per 2020 EORTC/MSG disease definitions (Appendix 9).

   - <u>Possible IA</u> includes participants with clinical criteria, with the anticipation that further diagnostic workup is in progress as clinically feasible and may result in updated classification of the invasive fungal infection as per the 2020 EORTC/MSG disease definitions (Appendix 9).
   - <u>Probable IA</u> includes participants with clinical criteria, along with mycological criteria including serum, plasma, CSF, or BAL fluid *Aspergillus* galactomannan antigen, or *Aspergillus* PCR test positive in plasma, serum, whole blood, or BAL fluid, or evidence of *Aspergillus* by histology or microscopy, or positive culture of a specimen taken by nonsterile sampling of an infected site as per 2020 EORTC/MSG disease definitions (Appendix 9).

4. If enrolled with a proven IA diagnosis, has demonstrated fungal elements (by cytology, microscopy, or histopathology, including *Aspergillus* nucleic acid probe where available) or positive culture for *Aspergillus* of a tissue specimen obtained from an otherwise sterile site as per 2020 EORTC/MSG disease definitions (Appendix 9).

5. Has a central line (eg, central venous catheter, peripherally inserted central catheter) in place or planned to be in place before beginning IV study treatment.

6. Has clinical symptoms consistent with an acute episode of IA, defined as duration of clinical syndrome of <30 days.

Demographics

7. Is male or female, and ≥2 years of age and <18 years of age at the time of first dose of study treatment and weighs at least 10 kg. Participants may be of any race/ethnicity.

Male Participants

Contraceptive use by males should be consistent with local regulations regarding the methods of contraception for those participating in clinical studies.

8. Male participants are eligible to participate if they agree to the following during the intervention period and for at least 30 days after the last dose of study treatment:

- Be abstinent from heterosexual intercourse as their preferred and usual lifestyle (abstinent on a long term and persistent basis) and agree to remain abstinent

  OR

- Must agree to use contraception unless confirmed to be azoospermic (vasectomized or secondary to medical cause Appendix 5) as detailed below:

  - Agree to use a male condom plus partner use of an additional contraceptive method when having penile-vaginal intercourse with a female of childbearing potential who is not currently pregnant. Note: Males with a pregnant or breastfeeding partner must agree to remain abstinent from penile-vaginal intercourse or use a male condom during each episode of penile-vaginal penetration.

Female Participants

Contraceptive use by females should be consistent with local regulations regarding the methods of contraception for those participating in clinical studies.

9. A female participant is eligible to participate if she is not pregnant or breastfeeding, and at least one of the following conditions applies:

   - Is not a female of childbearing potential

OR

- Is a female of childbearing potential and using a contraceptive method that is highly effective (with a failure rate of <1% per year), with low user dependency, or be abstinent from heterosexual intercourse as their preferred and usual lifestyle (abstinent on a long term and persistent basis), as described in Appendix 5 during the intervention period and for at least 30 days after the last dose of study intervention. The investigator should evaluate the potential for contraceptive method failure (ie, noncompliance, recently initiated) in relationship to the first dose of study intervention.

- A female of childbearing potential must have a negative highly sensitive pregnancy test (urine or serum as required by local regulations) within 24 hours before the first dose of study intervention.

- Additional requirements for pregnancy testing during and after study intervention are located in Appendix 2.

- The investigator is responsible for review of medical history, menstrual history, and recent sexual activity to decrease the risk for inclusion of a female with an early undetected pregnancy.

Informed Consent/Assent

10. The participant (or legally acceptable representative) has provided documented informed consent/assent for the study.

See Section 10.7, Appendix 7 for country-specific inclusion criteria requirements.

5.2 Exclusion Criteria

The participant must be excluded from the study if the participant:

Medical Conditions

1. Has chronic (≥30 days' duration) IA, relapsed/recurrent IA, or refractory IA that has not responded to prior antifungal treatment.

2. Has cystic fibrosis, pulmonary sarcoidosis, aspergilloma, or allergic bronchopulmonary aspergillosis.

3. Has known hypersensitivity or other serious adverse reaction to any azole antifungal therapy, or to any other ingredient of the study treatment used.

4. Has any known history of torsade de pointes, unstable cardiac arrhythmia or proarrhythmic conditions, a history of recent myocardial infarction, congenital or acquired QT prolongation, or cardiomyopathy in the context of cardiac failure within 90 days of time of first dose of study treatment.

5. Has known hereditary fructose intolerance.

6. Has known galactose intolerance, Lapp lactase deficiency, or glucose-galactose malabsorption.

7. Is on artificial ventilation at the time of first dose of study treatment.

8. Has any condition that, in the opinion of the investigator, may interfere with optimal participation in the study.

Prior/Concomitant Therapy

9. Has received any treatment specifically listed in Table 2 within the specified timeframes before the start of study treatment.

Table 2 Prohibited Medications Before Start of Study Treatment

**Prohibited Medications Before Start of Study Treatment**	**Prohibited Time Period Before Day 1 of Study Treatment[a]**
Any investigational drug (new chemical or biological entities): may be permitted with the approval of the Sponsor's Clinical Director	Only permitted with approval of the Sponsor's Clinical Director prior to start of study therapy
Systemic antifungal treatment (oral, intravenous, or inhaled) when used to treat current fungal infection episode	Prohibited if duration of treatment for current fungal infection is ≥96 hours before Day 1
Azole antifungal prophylaxis with mold activity (ie, itraconazole, voriconazole, isavuconazole)	Prohibited unless approval provided by the study Sponsor's Clinical Director prior to study randomization. Without Sponsor approval, prohibited if duration of mold-active azole prophylaxis therapy exceeds 13 days prior to study randomization
Astemizole	Prohibited if taken within 24 hours before Day 1
Cisapride, ebastine, halofantrine, pimozide, quinidine, and terfenadine	Prohibited if taken within 24 hours before Day 1
Ergot alkaloids (ergotamine, dihydroergotamine, or other members of this class)	Prohibited if taken within 24 hours before Day 1

Prohibited Medications Before Start of Study Treatment	Prohibited Time Period Before Day 1 of Study Treatment[a]
Disopyramide	Prohibited if taken within 24 hours before Day 1
Bepridil	Prohibited if taken within 24 hours before Day 1
Ibutilide	Prohibited if taken within 24 hours before Day 1
Haloperidol	Prohibited if taken within 24 hours before Day 1
HMG-CoA reductase inhibitors metabolized via CYP3A4 (eg, simvastatin, lovastatin, and atorvastatin)	Prohibited if taken within 24 hours before Day 1
Atazanavir, efavirenz, fosamprenavir, or ritonavir	Prohibited if taken within 24 hours before Day 1
Venetoclax	Prohibited if taken within 24 hours of Day 1

Abbreviations: CYP = cytochrome P450; HMG-CoA=beta-hydroxy-beta-methylglutaryl-CoA.

[a] These waiting times should be observed immediately before start of study treatment and during study treatment in participants receiving a prohibited treatment as prior treatment.

Prior/Concurrent Clinical Study Experience

10. Has enrolled previously in the current study and been discontinued.

Diagnostic Assessments

11. Has QTc prolongation (based on either Fridericia or Bazett's correction) at screening >500 msec.

12. Has significant liver dysfunction (defined as total bilirubin >1.5 × ULN AND AST or ALT >3 × ULN with normal alkaline phosphatase) at screening.

13. Has calculated creatinine clearance <20 mL/min/1.73 m^2 (modified Schwartz formula) at screening. Creatinine clearance will be calculated using the following equation:

Modified Schwartz formula

$$\text{Creatinine clearance} = \frac{K \times (\text{height in cm})}{(\text{creatinine in mg/dL})}$$

K (proportionality constant):
Male child (≥2 year to <13 years): K=0.55
Male child (≥13 year to <18 years): K=0.70
Female child (≥2 year to <18 years): K=0.55

Other Exclusions

14. Is not expected, in the opinion of the investigator, to survive for at least 1 month after the initiation of study treatment.

15. Is or has an immediate family member (eg, spouse, parent/legal guardian, sibling, or child) who is investigational site or Sponsor staff directly involved with this study.

See Section 10.7, Appendix 7 for country-specific exclusion criteria requirements.

5.3 Lifestyle Considerations

There are no diet or activity restrictions for this study.

5.4 Screen Failures

Screen failures are defined as participants who consent/assent to participate in the clinical study but are not subsequently entered into the study. A minimal set of screen failure information is required to ensure transparent reporting of screen failure participants to meet the Consolidated Standards of Reporting Trials (CONSORT) publishing requirements and to respond to queries from regulatory authorities. Minimal information includes demography, screen failure details, eligibility criteria, and any adverse events or serious adverse events (SAE) meeting reporting requirements as outlined in the data entry guidelines.

5.5 Participant Replacement Strategy

A participant who discontinues from study treatment or withdraws from the study will not be replaced.

6 STUDY INTERVENTION

Study treatment is defined as any investigational treatment(s), marketed product(s), placebo, or medical device(s) intended to be administered to a study participant according to the study protocol.

Clinical supplies (study interventions provided by the Sponsor) will be packaged to support enrollment. Clinical supplies will be affixed with a clinical label in accordance with regulatory requirements.

6.1 Study Intervention(s) Administered

The interventions to be used in this study are outlined in Table 3. For dosing of the PFS see Table 1.

Table 3 Study Treatments

Arm Name	Arm Type	Intervention Name	Intervention Type	Dose Formu-lation	Unit Dose Strength(s)	Dosage Level(s)	Route of Adminis-tration	Regimen/ Treatment Period	Use	IMP or NIMP/AxMP	Sourcing
Posaconazole	Experi-mental	IV	Drug	Vial	18 mg/mL	6 mg/kg<br><br>Doses are not to exceed 300 mg per administration	IV Infusion	Day 1: BID<br><br>Day 2 through end of IV dosing: QD	Test Product	IMP	Centrally sourced with the ability to source locally if required
Posaconazole	Experi-mental	Powder for suspension (PFS)	Drug	Powder for Suspension	30 mg/mL	Dosing based on weight-band.<br>To be administered to participants ≤40 kg	Oral	Days 8 – 84: QD	Test Product	IMP	Centrally sourced with the ability to source locally if required
Posaconazole	Experi-mental	Tablet	Drug	Tablet	100 mg	300 mg<br><br>To be administered to participants >40 kg	Oral	Days 8 – 84: QD	Test Product	IMP	Centrally sourced with the ability to source locally if required

Abbreviations: AxMP Auxiliary Medical Product, EEA=European Economic Area, IMP= Investigational medical product, NIMP=non-investigational medical product, QD= Once daily

The classification of Investigational Medicinal Product (IMP) and Non-Investigational Medicinal Product (NIMP) in this table is based on guidance issued by the European Commission and applies to countries in the European Economic Area (EEA). Country differences with respect to the definition/classification of IMP/NIMP may exist. In these circumstances, local legislation is followed.

All supplies indicated in Table 3 will be provided per the "Sourcing" column depending upon local country operational requirements. If local sourcing, every attempt should be made to source these supplies from a single lot/batch number where possible (eg, not applicable in the case where multiple lots or batches may be required due to the length of the study, etc).

Refer to Section 8.1.8 for details regarding administration of the study intervention.

6.2 Preparation/Handling/Storage/Accountability

6.2.1 Dose Preparation

The rationale for the selection of doses to be used in this study is provided in Section 4.3, Justification for Dose.

The participant's body weight at Day 1 will be used to calculate the dose of POS IV study treatment. The participant's body weight as measured within 3 days before the first day of oral POS treatment will be used to calculate all subsequent doses of oral POS: any participant whose weight is ≤40 kg will transition to the PFS formulation; any participant whose weight is >40 kg will transition to the tablet formulation and will receive 300 mg as 3 × 100-mg tablets. For any subsequent switch from oral to IV formulation, the IV dose will be calculated based on the participant's weight before the initial switch to an oral formulation.

Instructions for preparation of the IV infusion and the PFS oral solution are found in the Pharmacy Manual. Instructions for administration of PFS will be provided to caregivers separately.

6.2.2 Handling, Storage, and Accountability

The investigator or designee must confirm appropriate temperature conditions have been maintained during transit for all study intervention received, and any discrepancies are reported and resolved before use of the study intervention.

Only participants enrolled in the study may receive study intervention, and only authorized site staff may supply or administer study intervention. All study interventions must be stored in a secure, environmentally controlled, and monitored (manual or automated) area in accordance with the labeled storage conditions with access limited to the investigator and authorized site staff.

The investigator, institution, or the head of the medical institution (where applicable) is responsible for study intervention accountability, reconciliation, and record maintenance (ie, receipt, reconciliation, and final disposition records).

For all study sites, the local country Sponsor personnel or designee will provide appropriate documentation that must be completed for drug accountability and return, or local discard and destruction if appropriate. Where local discard and destruction is appropriate, the investigator is responsible for ensuring that a local discard/destruction procedure is documented.

The study site is responsible for recording the lot number, manufacturer, and expiry date for any locally purchased product (if applicable) as per local guidelines unless otherwise instructed by the Sponsor.

The investigator shall take responsibility for and shall take all steps to maintain appropriate records and ensure appropriate supply, storage, handling, distribution, and usage of study interventions in accordance with the protocol and any applicable laws and regulations.

6.3 Measures to Minimize Bias: Randomization and Blinding

6.3.1 Intervention Assignment

Participants in this study will be allocated by nonrandom assignment.

All participants will receive POS as study treatment throughout the study, with the initial formulation for all participants being the IV formulation. Allocation numbers will be assigned by an IRT system.

6.3.2 Stratification

Treatment randomization/allocation will be stratified according to the following factors:

Participant's age at the time of first dose of study treatment:

1. Age Cohort 1: 2 years to <12 years of age.
2. Age Cohort 2: 12 years to <18 years of age.

6.3.3 Blinding

This study is an open-label study; therefore, the Sponsor, investigator and participant will know the treatment administered.

6.4 Study Intervention Compliance

The volume of injection and the number of tablets/sachets remaining in study packaging will be counted, reviewed, and recorded at regular intervals to monitor treatment compliance.

The shifting of the timing of a dose (within the window provided in Section 8.1.8 to accommodate a participant's schedule) is not considered an interruption.

Interruptions from the protocol-specified treatment plan for ≥7 days require consultation between the investigator and the Sponsor and written documentation of the collaborative decision on participant management.

6.5 Concomitant Therapy

Medications or vaccinations specifically prohibited in the exclusion criteria are not allowed during time periods specified by this protocol for that medication. If there is a clinical

indication for any medications or vaccinations specifically prohibited, discontinuation from study intervention may be required. The investigator should discuss any questions regarding this with the Sponsor Clinical Director. The final decision on any supportive therapy or vaccination rests with the investigator and/or the participant's primary physician. However, the decision to continue the participant on study intervention requires the mutual agreement of the investigator, the Sponsor, and the participant.

Medications prohibited during study treatment are presented in Table 4.

Table 4 Prohibited Medications During Study Treatment

**Prohibited Medications During Study Treatment**	**Time Period[a]**
Any investigational drug (new chemical or biological entities): Investigational use of approved products or chemotherapy regimens may be permitted with the approval of the Sponsor's Clinical Director before use.	Prohibited during study treatment
Systemic antifungals other than echinocandins (oral, intravenous, or inhaled) as outlined below	Prohibited during study treatment
Echinocandin antifungals (caspofungin, anidulafungin, micafungin)	Prohibited during study treatment except when given for the treatment of invasive candidiasis for a maximum duration of 14 days
Astemizole	Prohibited during study treatment
Cisapride, ebastine, halofantrine, pimozide, quinidine, and terfenadine	Prohibited during study treatment
Ergot alkaloids (ergotamine, dihydroergotamine, or other members of this class)	Prohibited during study treatment
Disopyramide	Prohibited during study treatment
Bepridil	Prohibited during study treatment
Ibutilide	Prohibited during study treatment
Haloperidol	Prohibited during study treatment
Barbiturates, carbamazepine, cimetidine, isoniazid, phenytoin, rifabutin, rifampin, and St. John's Wort (*Hypericum perforatum*)	Prohibited during study treatment
HMG-CoA reductase inhibitors metabolized via CYP3A4 (eg, simvastatin, lovastatin, and atorvastatin)	Prohibited during study treatment
Atazanavir, efavirenz, fosamprenavir, or ritonavir	Prohibited during study treatment
Venetoclax	Prohibited during study treatment

Abbreviations: CYP = cytochrome P450; HMG-CoA=beta-hydroxy-beta-methylglutaryl-CoA.

[a] Medications prohibited during study treatment should not be given during study treatment and for 7 days following the cessation of study treatment. Any deviations to this time period must be discussed with the study team/clinical monitor before initiating any concomitant therapies listed above.

All medications not listed in Table 4 are permitted in the study. Specific medications that are allowed with caution and/or monitoring are presented in Section 6.5.1.

The following topical nonabsorbable antifungals may be used for the treatment of oropharyngeal candidiasis, vaginal candidiasis, or cutaneous fungal infection: oral amphotericin B, miconazole (oral or topical), nystatin (oral or topical), and clotrimazole (oral or topical). All other antifungal therapies must be approved by the Sponsor before use.

6.5.1 Medications Allowed With Caution and Clinical Monitoring

Clinical and/or QTc monitoring is recommended when the study treatment is coadministered with one of the following drugs that have reported a potential risk of torsades de pointes:

- Antiarrhythmics (amiodarone, dofetilide, procainamide, sotalol)
- Chlorpromazine
- Clarithromycin
- Domperidone
- Droperidol
- Levomethadyl
- Mesoridazine
- Methadone
- Erythromycin
- Sparfloxacin
- Thioridazine

Medications that are allowed with caution and/or monitoring are presented in Table 5. These drugs are permitted, although their efficacy and safety should be clinically monitored and/or serum levels followed with appropriate dosage adjustments as necessary at the initiation of study treatment, periodically during treatment, and after discontinuation of study treatment.

Table 5 Additional Medications Allowed During the Study That Require Monitoring and/or Dose Adjustment

**Medication Class**	**Medication Name[a]**	**Recommendation**
Anticoagulants	Coumadin-type	Coumadin-type anticoagulants are permitted, although their efficacy and safety should be clinically monitored and/or serum levels followed with appropriate dosage adjustments as necessary at the initiation of study treatment, periodically during treatment, and after discontinuation of study treatment.
Antiretroviral Therapy	Multiple	As HIV protease inhibitors and NNRTIs are CYP3A4 substrates, it is expected that POS will increase plasma levels of these antiretroviral agents. Participants should be carefully monitored for any occurrence of toxicity during the coadministration of POS and these agents.<br>NOTE: Coadministration of POS with atazanavir, efavirenz, fosamprenavir, or ritonavir is not permitted during the treatment phase of the study.
Benzodiazepines	Alprazolam<br>Midazolam<br>Triazolam	POS 200 mg orally once daily increased the AUC of midazolam by 83% following IV administration. Due to the inhibition of intestinal CYP3A4 by POS, an even greater effect of POS on the AUC of midazolam is expected following oral administration. Dose adjustments should be considered for all benzodiazepines that are metabolized through CYP3A4. POS interferes with the hepatic clearance of triazolam and midazolam, and thus, may enhance the sedative effects of these agents. Therefore, these agents should not be used unless monitoring is provided for excessive sedation
Calcium Channel Blockers	Diltiazem<br>Nifedipine<br>Nisoldipine<br>Verapamil	For calcium channel blockers metabolized through CYP3A4 (eg, diltiazem, nifedipine, nisoldipine, verapamil), frequent monitoring for AEs and toxicity related to calcium channel blockers is recommended during coadministration with POS. Dose adjustment of calcium channel blockers may be required.
Cardiac Glycosides	Digoxin	Administration of other azoles has been associated with increases in digoxin levels. Digoxin levels should be monitored when initiating or discontinuing POS treatment.

Medication Class	Medication Name[a]	Recommendation
Antineoplastic/ Immunosuppressive	Cyclophosphamide	Periodic blood work should be obtained to monitor complete blood count as well as the function of other organs (such as kidneys and liver). Toxicity monitoring to include monitoring for potential myelosuppression, bone marrow failure, urinary tract and renal toxicity, cardiotoxicity including myocarditis, congestive heart failure, and arrhythmia, and pulmonary toxicity. Close monitoring for potential toxicity should occur during coadministration.
Antineoplastic	Trisenox	Prior to initiating therapy with Trisenox, a 12-lead ECG should be performed, and serum electrolytes (potassium, calcium, and magnesium) and creatinine should be assessed. Pre-existing electrolyte abnormalities should be corrected and, if possible, drugs that are known to prolong the QT interval should be discontinued. If it is not possible to discontinue the interacting drug, perform cardiac monitoring frequently. Monitor ECG weekly, and more frequently for clinically unstable patients.
Chemotherapeutic Agents	Anthracycline-based chemotherapeutics	During coadministration, close monitoring for potential cardiotoxicity and hepatotoxicity should occur.
Chemotherapeutic Agents	Vinca alkaloids (eg, vincristine and vinblastine)	Concomitant administration of azole antifungals, including POS, may increase the plasma concentrations of vinca alkaloids, which may lead to neurotoxicity and other serious adverse reactions. Reserve azole antifungals, including POS, for patients receiving a vinca alkaloid, including vincristine, who have no alternative antifungal treatment options.
Immunosuppressive	Sirolimus	Concomitant administration of POS with sirolimus increases the sirolimus blood concentrations by approximately 9-fold and can result in sirolimus toxicity. Monitoring of sirolimus blood levels should be performed on initiation, during coadministration, and on discontinuation of POS treatment, with adjustment of sirolimus doses as necessary.
Immunosuppressive	Cyclosporine	In heart transplant, patients on stable doses of cyclosporine, POS 200 mg QD increased cyclosporine concentrations requiring dose reductions of up to 29%. Cases of elevated cyclosporine concentrations resulting in SAEs, including nephrotoxicity were reported in clinical efficacy studies. Monitoring of cyclosporine blood levels should be performed on initiation, during coadministration, and on discontinuation of POS treatment, with adjustment of cyclosporine doses as necessary.

**Medication Class**	**Medication Name[a]**	**Recommendation**
Immunosuppressive	Tacrolimus	POS increased Cmax and AUC of tacrolimus (0.05 mg/kg body weight single-dose) by 121% and 358%, respectively. Clinically significant interactions resulting in hospitalization and/or POS discontinuation were reported in clinical efficacy studies. When initiating POS treatment in participants already receiving tacrolimus, the dose of tacrolimus dose should be reduced (eg, to about one-third of the current dose). Thereafter, blood levels of tacrolimus should be monitored carefully during coadministration, and on discontinuation of POS, and the dose of tacrolimus should be adjusted as necessary.
Oral Hypoglycemic Agents/Sulfonylureas	Glipizide	Glucose concentrations decreased in some healthy participants when glipizide was coadministered with POS. Monitoring of glucose concentrations is recommended in diabetic participants.
Tyrosine kinase inhibitors	Ibrutinib	During coadministration with POS, dose adjustment of ibrutinib per product label should occur. Interruption of ibrutinib dose/dose modification for Grade 3 or greater hematologic toxicity is recommended.
Xanthine Derivatives	Theophylline	Theophylline is permitted, although its efficacy and safety should be clinically monitored and/or serum levels followed with appropriate dosage adjustments as necessary at the initiation of study treatment, periodically during treatment, and after discontinuation of study treatment.

Abbreviations: AUC=area under the concentration-time curve; Cmax=observed maximal concentration; CYP=cytochrome P450; ECG=electrocardiogram; HIV=human immunodeficiency virus; IV=intravenous; NNRTI=non-nucleoside reverse transcriptase inhibitor; POS=posaconazole; QD=once daily; SAE=serious adverse event.

[a] Not all medications from a specific medication class are necessarily listed here.

6.5.2 Rescue Medications and Supportive Care

No rescue or supportive medications are specified for use in this study.

6.6 Dose Modification (Escalation/Titration/Other)

No dose modifications are planned in this study.

6.7 Intervention After the End of the Study

There is no study-specified treatment following the end of the study. Following the completion of study treatment, study participants may continue to receive additional non-study antifungal treatment for the episode of IA or as secondary antifungal prophylaxis as clinically indicated.

6.8 Clinical Supplies Disclosure

This study is open-label; therefore, the participant, the study site personnel, the Sponsor and/or designee are not blinded. Study treatment (name, strength or potency) is included in the label text; random code/disclosure envelopes or lists are not provided.

7 DISCONTINUATION OF STUDY INTERVENTION AND PARTICIPANT WITHDRAWAL

7.1 Discontinuation of Study Intervention

Discontinuation of study intervention does not represent withdrawal from the study.

As certain data on clinical events beyond study intervention discontinuation may be important to the study, they must be collected through the participant's last scheduled follow-up, even if the participant has discontinued study intervention. Therefore, all participants who discontinue study intervention before completion of the protocol-specified treatment period will still continue to participate in the study as specified in Section 1.3 and Section 8.9.2.3.

Participants may discontinue study intervention at any time for any reason or be discontinued from the study intervention at the discretion of the investigator should any untoward effect occur. In addition, a participant may be discontinued from study intervention by the investigator or the Sponsor if study intervention is inappropriate, the study plan is violated, or for administrative and/or other safety reasons. Specific details regarding procedures to be performed at study intervention discontinuation are provided in Section 8.1.9 and Section 8.9.2.3.

A participant must be discontinued from study intervention but continue to be monitored in the study for any of the following reasons:

- The participant or participant's legally acceptable representative requests to discontinue study intervention.
- The participant has a medical condition or personal circumstance, which, in the opinion of the investigator and/or Sponsor, placed the participant at unnecessary risk from continued administration of study intervention.
- The participant has a confirmed positive pregnancy test.
- Serious adverse experience related to study therapy or the occurrence of an adverse experience (clinical or laboratory) that, in the judgment of the investigator, warrants withdrawal of study therapy.
- If the QTc interval is >500 msec (based on either Fridericia or Bazett's correction), study therapy may be interrupted for up to 3 days with concomitant monitoring and correction of electrolytes. Study therapy may be resumed if QTc interval decreases to <500 msec.

- If during study treatment the participant has AST or ALT ≥3 × ULN and bilirubin ≥2 × ULN and alkaline phosphatase <2 × ULN, then study therapy may be interrupted for up to 3 days with repeat monitoring of liver function testing. Study therapy may be resumed if repeated liver function testing parameters no longer meet the criteria outlined above and the change is unrelated to study intervention based on the opinion of the investigator. Study therapy should be permanently discontinued if liver function testing continues to exceed the specified parameters with repeat testing regardless of relationship to study medication.

For participants who are discontinued from study intervention but continue to be monitored in the study, all visits and procedures, as outlined in the SoA, should be completed.

See Section 10.7, Appendix 7 for country-specific discontinuation of study intervention requirements.

7.2 Participant Withdrawal From the Study

A participant must be withdrawn from the study if the participant or participant's legally acceptable representative withdraws consent from the study.

If a participant withdraws from the study, they will no longer receive study treatment or be followed at scheduled protocol visits.

Specific details regarding procedures to be performed at the time of withdrawal from the study are outlined in Section 8.1.9 – Withdrawal/Discontinuation. Participants with possible IA for whom proven or probable IA cannot be confirmed will not be withdrawn from the study. The procedures to be performed should a participant repeatedly fail to return for scheduled visits and/or if the study site is unable to contact the participant are outlined in Section 7.3.

7.3 Lost to Follow-up

If a participant fails to return to the clinic for a required study visit and/or if the site is unable to contact the participant, the following procedures are to be performed:

- The site must attempt to contact the participant and reschedule the missed visit. If the participant is contacted, the participant should be counseled on the importance of maintaining the protocol-specified visit schedule.

- The investigator or designee must make every effort to regain contact with the participant at each missed visit (eg, phone calls and/or a certified letter to the participant's last known mailing address or locally equivalent methods). These contact attempts should be documented in the participant's medical record.

  Note: A participant is not considered lost to follow-up until the last scheduled visit for the individual participant. The missing data for the participant will be managed via the pre-specified statistical data handling and analysis guidelines.

8 STUDY ASSESSMENTS AND PROCEDURES

- Study procedures and their timing are summarized in the SoA.
- Adherence to the study design requirements, including those specified in the SoA, is essential and required for study conduct.
- The investigator is responsible for assuring that procedures are conducted by appropriately qualified or trained staff. Delegation of study site personnel responsibilities will be documented in the Investigator Trial File Binder (or equivalent).
- All screening evaluations must be completed and reviewed to confirm that potential participants meet all eligibility criteria. The investigator will maintain a screening log to record details of all participants screened and to confirm eligibility or record reasons for screening failure, as applicable.
- Procedures conducted as part of the participant's routine clinical management (eg, blood count) and obtained before documented ICF may be utilized for screening or baseline purposes provided the procedure met the protocol-specified criteria and were performed within the time frame defined in the SoA.
- Additional evaluations/testing may be deemed necessary by the investigator and or the Sponsor for reasons related to participant safety. In some cases, such evaluation/testing may be potentially sensitive in nature (eg, HIV, Hepatitis C, etc.), and thus local regulations may require that additional informed consent, and assent if applicable, be obtained from the participant. In these cases, such evaluations/testing will be performed in accordance with those regulations.

The maximum amount of blood collected from each participant over the duration of the trial, including any extra assessments that may be required, will not exceed 78.6 mL Table 12.

Repeat or unscheduled samples may be taken for safety reasons or for technical issues with the samples.

8.1 Administrative and General Procedures

8.1.1 Informed Consent/Assent

The investigator or medically qualified designee (consistent with local requirements) must obtain documented informed consent, and assent if applicable, from each potential participant or their legally acceptable representative prior to participating in this clinical study. If there are changes to the participant's status during the study (eg, health or age of majority requirements), the investigator or medically qualified designee must ensure the appropriate documented consent is in place.

8.1.1.1 General Informed Consent/Assent

Informed consent/assent given by the participant or their legally acceptable representative must be documented on a consent form. The form must include the trial protocol number, trial protocol title, dated signature, and agreement of the participant (or his/her legally acceptable representative) and of the person conducting the consent/assent discussion.

A copy of the signed and dated informed consent/assent form should be given to the participant (or their legally acceptable representative) before participation in the study.

The initial ICF, any subsequent revised ICF, and any written information provided to the participant must receive the IRB/IEC's approval/favorable opinion in advance of use. The participant or his/her legally acceptable representative should be informed in a timely manner if new information becomes available that may be relevant to the participant's willingness to continue participation in the study. The communication of this information will be provided and documented via a revised consent/assent form or addendum to the original consent form that captures the participant's or participant's legally acceptable representative's dated signature.

If the investigator recommends continuation of study intervention beyond disease progression, the participant or their legally acceptable representative will be asked to provide documented informed consent/assent.

Specifics about the study and the study population are to be included in the study informed consent/assent form.

Informed consent/assent will adhere to IRB/IEC requirements, applicable laws and regulations, and Sponsor requirements.

8.1.2 Inclusion/Exclusion Criteria

All inclusion and exclusion criteria will be reviewed by the investigator, who is a qualified physician, to ensure that the participant qualifies for the study.

8.1.3 Participant Identification Card

All participants will be given a Participant Identification Card identifying them as participants in a research study. The card will contain study site contact information (including direct telephone numbers) to be utilized in the event of an emergency. The investigator or qualified designee will provide the participant with a Participant Identification Card immediately after the participant provides documented informed consent/assent. At the time of treatment allocation/randomization, site personnel will add the treatment/randomization number to the Participant Identification Card.

8.1.4 Medical History

A medical history will be obtained by the investigator or qualified designee. The medical history should include details regarding underlying conditions that may qualify the participant for possible, probable, or proven IA according to 2020 EORTC/MSG criteria (Appendix 9). The medical history should include details regarding any ongoing or prior cancer diagnoses, immunosuppressive conditions (including HIV), transplant procedures (HSCT or solid organ), and any history of graft-versus-host disease (acute or chronic). The history should also include details regarding recent or ongoing bacterial infectious episodes and any prior histories of invasive fungal infection, as well as details regarding the current episode of IA. Any history of hepatitis should also be recorded.

8.1.5 Prior and Concomitant Medications Review

8.1.5.1 Prior Medications

The investigator or qualified designee will review prior medication use, including any protocol-specified washout requirement and prohibited medications, for the 14 days before initiation of POS treatment. Chemotherapeutic agents used for any chemotherapy regimen for active treatment of malignancy, as well as T-cell and B-cell immunosuppressive therapies and antifungal treatment within 30 days before the first dose of study treatment, are to be recorded.

- Record prior medication (prescription or over the counter) taken within 14 days before starting the trial, including protocol-specified prohibited medications, as outlined in Section 5.2 (Table 2).
- The identity of the therapy, the dates started and stopped (or notation of "continuing" if that is the case), and the reason for use must be recorded for all medications.
- Participants receiving other systemic antifungal agents (IV, oral or nasal/inhaled) as prophylactic therapy must discontinue these treatments before study treatment administration. No other systemic antifungal agent may be administered during the study treatment phase (ie, POS IV solution/PFS/tablet) without Sponsor approval.

8.1.5.2 Concomitant Medications

The investigator or qualified designee will record medication, if any, taken by the participant during the study. The identity of the therapy, the dates started and stopped (or notation of continuing if that is the case), and the reason for use must be recorded. The use of any concomitant medication must relate to an AE or the participant's medical history.

8.1.6 Assignment of Screening Number

All consented participants will be given a unique screening number that will be used to identify the participant for all procedures that occur prior to treatment allocation. Each participant will be assigned only one screening number. Screening numbers must not be re-used for different participants.

Specific details on the screening visit requirements are provided in Section 8.9.1.

8.1.7 Assignment of Treatment/Randomization Number

All eligible participants will be allocated, by nonrandom assignment, and will receive a treatment/randomization number. The treatment/randomization number identifies the participant for all procedures occurring after treatment allocation/randomization. Once a treatment/randomization number is assigned to a participant, it can never be re-assigned to another participant.

A single participant cannot be assigned more than 1 treatment/randomization number.

8.1.8 Study Intervention Administration

IV infusions should be administered via a central line at approximately the same time each day. On the first 24 hours of IV dosing, there is a loading period during which 2 doses are given, separated by 12 hours. Subsequently, a single dose is given once daily. For the third dose, the dose should be timed to be given approximately 24 hours after the initial loading dose (ie, approximately 12 hours after the second loading dose). All subsequent IV doses should be administered approximately 24 hours apart. If for any reason the timing of the medication needs to be adjusted after the first dose, the dose time may be adjusted by ±3 hours.

Doses of POS PFS and tablets will be administered every 24 hours during the oral treatment period, without regard to timing of food intake. If for any reason, the timing of the medication needs to be adjusted after the first dose, the dose time may be adjusted by ± 3 hours. Participants who vomit within 30 minutes of POS PFS or tablet administration should be given a replacement dose as soon as the participant is able to take the new dose. Repeated episodes of vomiting should be recorded on the AE electronic case report form (eCRF) and the Study Medication eCRF.

If the dosing of study treatment is delayed, the dose should be administered as soon as possible. If it is more than 6 hours since the missed dose, the missed dose should be skipped, and the next dose should be administered at the regularly scheduled time. Any missed doses should be properly documented in the participant's eCRF and source documentation.

8.1.9 Discontinuation and Withdrawal

Participants who discontinue study treatment prior to completion of the treatment period should be encouraged to continue to be followed for all remaining study visits.

When a participant withdraws from participation in the study, all applicable activities scheduled for the EOT Visit should be performed (at the time of withdrawal). Any adverse events which are present at the time of withdrawal should be followed in accordance with the safety requirements outlined in Section 8.4.

In addition, participants and caregivers/legally acceptable representatives should be informed that the site will attempt to contact them at Day 114 for a mortality assessment.

8.1.10 Participant Blinding/Unblinding

This is an open-label study; there is no blinding for this study.

8.1.11 Calibration of Equipment

The investigator or qualified designee has the responsibility to ensure that any device or instrument used for a clinical evaluation/test during a clinical study that provides information about inclusion/exclusion criteria and/or safety or efficacy parameters shall be suitably calibrated and/or maintained to ensure that the data obtained are reliable and/or reproducible. Documentation of equipment calibration must be retained as source documentation at the study site.

8.2 Efficacy/Immunogenicity Assessments

8.2.1 Global Clinical Response

The investigator will assess global clinical response at the Week 6 and Week 12 visits; in the event of early therapy discontinuation, global clinical response will be assessed at the EOT Visit. All data related to the evaluation of global clinical response will be collected, including clinical signs and symptoms, imaging, serologic testing, and fungal culture and histology. A global clinical response will be assessed by the investigator as favorable (positive) if the participant is alive and has a complete or partial response, using the definitions of responses to therapy and study outcomes in clinical trials of invasive fungal diseases at the time points of interest [Segal, B. H., et al 2008] (Table 6).

Table 6 Global Clinical Response using 2008 EORTC/MSG Definitions

Outcome, Response	Definition
Success	
Complete response	Survival within the prespecified period of observation, resolution of all attributable symptoms and signs of disease, resolution of radiological lesion(s), and documented clearance of infected sites that are accessible to repeated sampling.
Partial response	Survival within the prespecified period of observation, improvement in attributable symptoms and signs of disease, improvement of radiological lesion(s)[a], and evidence of clearance of infected sites that are accessible to repeated sampling.<br>In the case of radiological stabilization[b], resolution of all attributable symptoms and signs of fungal disease; or where biopsy of an infected site shows no evidence of hyphae; or where culture is negative.
Failure	
Stable response	Survival within the prespecified period of observation and minor or no improvement in fungal disease; or persistent isolation of *Aspergillus spp* or histological present in infected sites.
Progression of fungal disease	Worsening of clinical symptoms and signs of disease plus new sites of disease or radiological worsening; or persistent isolation of *Aspergillus spp* from infected sites.
Death	Death during the prespecified period of evaluation, regardless of attribution.

[a] Improvement of radiological lesions is defined as at least 25% reduction in diameter of radiological lesion.
[b] Radiological stabilization is defined as 0%-25% reduction in the diameter of the lesion.

8.2.2 Diagnostic Imaging

High-resolution CT scan is required for all participants with pulmonary sites of infection, as chest X-ray is deemed insufficient for assessment of pulmonary IA. Each site of infection identified during screening should be followed throughout the study by repeating the same type of scan and the same imaging modality. All imaging will be performed locally, and evaluation of infection will be recorded on the eCRF. Recording of imaging should include sufficient details to reflect use of the 2020 EORTC/MSG criteria to support the classification of IA.

At screening and end of treatment, diagnostic imaging is required for all sites of infection. Imaging can be conducted at other times if the clinical infectious condition changes relative to the previous assessment and repeat imaging is judged warranted by the clinician. If diagnostic imaging is conducted before Screening and within 7 days of Day 1, the results may be used for screening purposes.

8.2.3 Mycology Testing

Mycology testing includes both histology samples and standard fungal cultures from all sites of suspected *Aspergillus* infection, as clinically appropriate. Mycology testing also includes galactomannan testing of plasma, serum, BAL, and CSF, as well as *Aspergillus* PCR testing of whole blood, serum, plasma, and BAL. Unless clinically inappropriate or not warranted due to the participant's health, condition or disease progression/regression, mycology testing may occur at the following visits: Screening, Week 6, Week 12, and EOT. Additional mycology testing will be performed as clinically indicated and should correlate with potential disease regression/progression. All fungal culture results (positive or negative) as well as all galactomannan and *Aspergillus* PCR test results (positive or negative) are to be recorded on the eCRF.

8.2.4 IFI Assessment

IFI assessment will be performed at every visit and should include all clinical signs and symptoms that may be related to fungal or bacterial infections. Any clinical sign or symptom that is possibly, probably, or definitely related to a fungal infection should be identified. All participants will be evaluated for the presence of IA based on 2020 EORTC/MSG definitions at all visits (Appendix 9).

8.2.5 Relapse Assessment

Relapse of IA infection will be evaluated only in participants who have achieved favorable clinical response. Relapse will be assessed by the investigator through 28 days following completion of study treatment in those participants who have achieved a favorable clinical response.

8.2.6 Mortality Assessment

For each participant, including those who withdraw from the study, a survival assessment (ie, whether the participant is alive or has died) will be performed through Day 114. In the event of death, the cause and date of death will be recorded. This assessment can occur by review of medical records or by telephone contact with the participant or a family member/legally acceptable representative.

8.2.7 *Aspergillus* Galactomannan EIA and PCR

Samples (ie, serum, BAL, plasma, or CSF) may be collected for *Aspergillus* galactomannan enzyme immunoassay (EIA) at Screening and subsequent visits to support the IA diagnosis. Samples (plasma, serum, whole blood, and BAL) may also be collected for *Aspergillus* PCR testing. The quantitative value and time of collection of the galactomannan sample should be documented for all galactomannan tests performed on participants. The results of all galactomannan samples taken during the period from screening to the follow-up visit should be provided. The results (positive or negative) of all samples taken for *Aspergillus* PCR testing should be provided.

If samples for *Aspergillus* galactomannan EIA were collected before screening and within 7 days of Day 1, results may be used for screening purposes. Galactomannan EIA serological test results may not be used to confirm a probable diagnosis of IA if participants have taken piperacillin/tazobactam within 72 hours of serum sampling. Indirect testing criteria that are supportive of a diagnosis of probable IA include:

Galactomannan detected in plasma, serum, BAL, or CSF:

- Single serum or plasma ≥1.0
- BAL fluid ≥1.0
- Single serum or plasma ≥0.7 with BAL fluid ≥0.8
- CSF ≥1.0

Aspergillus PCR testing, any 1 of the following:

- Plasma, serum, or whole blood 2 or more consecutive PCR tests positive
- BAL fluid 2 or more duplicate PCR tests positive
- At least 1 PCR test positive in plasma, serum, or whole blood and 1 PCR test positive in BAL fluid

8.3 Safety Assessments

Details regarding specific safety procedures/assessments to be performed in this study are provided. The total amount of blood/tissue to be drawn/collected over the course of the study (from prestudy to poststudy visits), including approximate blood/tissue volumes drawn/collected by visit and by sample type per participant, can be found in Appendix 2.

Planned time points for all safety assessments are provided in the SoA.

8.3.1 Physical Examinations

A complete physical examination will be conducted by an investigator or medically qualified designee (consistent with local requirements) as per institutional standard. The complete physical examination includes the following assessments: general appearance, head, eyes, ears/nose/throat, neck, lymph nodes, skin, lungs, heart, abdomen, musculoskeletal, and neurologic evaluations. Breast, rectal, and genitourinary/pelvic exams should be performed when clinically indicated.

Height and weight will also be measured and recorded. Height measurements should be taken without shoes and be recorded to the nearest cm. Body weight at baseline will be used to calculate the participant's dose for the IV treatment phase of the study. The participant's weight measured within 3 days before the first dose of oral treatment should be used to calculate all subsequent doses of the PFS oral formulation, as well doses of the IV formulation if the participant subsequently switches from an oral to the IV formulation. Body

weight should be obtained without shoes. At each required visit, it is preferred that the weight be measured on the same scale for the same individual. Measurements should be recorded to the nearest 0.1 kg.

A brief directed physical examination will be conducted by an investigator or medically qualified designee (consistent with local requirements) per institutional standard at any other visit when clinically indicated.

Investigators should pay special attention to clinical signs related to previous serious illnesses.

8.3.2 Vital Signs

- Vital signs will include heart rate, blood pressure, respiratory rate, and body temperature.

8.3.3 Electrocardiograms

- Single 12-lead ECG will be obtained as outlined in the SoA using an ECG machine that automatically calculates the heart rate and measures PR, QRS, QT, and QTc intervals. Any clinically significant abnormality on study treatment must be followed until stabilization or return to baseline. Refer to Section 7.1 for QTc withdrawal criteria and additional QTc readings that may be necessary.

- For small participants whose size does not allow for a standard 12-lead ECG, a modified 12-lead ECG may be obtained.

Note: When the collection of vital signs, ECGs, and PK samples coincide, the blood samples for PK determination should be collected first (so that the PK samples are collected on time), followed by the vital signs, and then the ECG. It is preferred that the ECG be performed at the same time each day (eg, morning) to reduce diurnal variation.

8.3.4 Clinical Safety Laboratory Assessments

- Refer to Appendix 2 for the list of clinical laboratory tests to be performed and to the SoA for the timing and frequency.

- The investigator or medically qualified designee (consistent with local requirements) must review the laboratory report, document this review, and record any clinically relevant changes occurring during the study in the AE section of the case report form (CRF). The laboratory reports must be filed with the source documents. Clinically significant abnormal laboratory findings are those which are not associated with the underlying disease, unless judged by the investigator to be more severe than expected for the participant's condition.

- All protocol-required laboratory assessments, as defined in Appendix 2, must be conducted in accordance with local or central laboratory requirement, as applicable, and the SoA.

- If laboratory values from nonprotocol-specified laboratory assessments performed at the institution's local laboratory require a change in study participant management or are considered clinically significant by the investigator (eg, SAE or AE or dose modification), then the results must be recorded in the appropriate CRF (eg, SLAB).

- For any laboratory tests with values considered clinically significantly abnormal during participation in the study or within 14 days after the last dose of study intervention, every attempt should be made to perform repeat assessments until the values return to normal or baseline or if a new baseline is established as determined by the investigator.

8.3.5 Assessment of Palatability and Acceptability

Palatability and acceptability will be assessed using the Palatability Acceptance Assessment. Palatability is measured using a 5-point facial hedonic scale depicting various degrees of tolerance. Participants will also be asked to report any problems experienced while taking the medication. All participants will rate the palatability and acceptability of the PFS formulation on the first and last days of the PFS treatment phase.

The Palatability Acceptance Assessment should be implemented with the following recommendations:

- Ages 2 through 4 years: Completion by observer (parent/caregiver or health care provider).
- Ages 5 through 13 years: Combined completion: the child participant should complete the faces question and the observer should complete the remaining questions.
- Ages 14 through 17 years: Completion directly by the child participant when possible.

Observer assessments should be based on what the parent/legally acceptable representative/ caregiver/health care provider has observed directly during and after medication administration, including the participant's facial expressions, behavior, and what the participant says. Only individuals who have directly observed the participant taking the study treatment should complete the assessment.

8.4 Adverse Events (AEs), Serious Adverse Events (SAEs), and Other Reportable Safety Events

The definitions of an AE or SAE, as well as the method of recording, evaluating, and assessing causality of AE and SAE and the procedures for completing and transmitting AE, SAE, and other reportable safety event reports can be found in Appendix 3.

AE, SAEs, and other reportable safety events will be reported by the participant (or, when appropriate, by a caregiver, surrogate, or the participant's legally authorized representative).

The investigator and any designees are responsible for detecting, documenting, and reporting events that meet the definition of an AE or SAE as well as other reportable safety events. Investigators need to document if an AE and/or SAE was associated with a medication error, misuse, or abuse.

Investigators remain responsible for following up AE, SAEs, and other reportable safety events for outcome according to Section 8.4.3.

The investigator, who is a qualified physician, will assess events that meet the definition of an AE or SAE as well as other reportable safety events with respect to seriousness, intensity/toxicity and causality.

8.4.1 Time Period and Frequency for Collecting AE, SAE, and Other Reportable Safety Event Information

All AEs, SAEs, and other reportable safety events that occur after the participant provides documented informed consent but before intervention allocation must be reported by the investigator if the participant is receiving placebo run-in or other run-in treatment, if the event causes the participant to be excluded from the study, or is the result of a protocol-specified intervention, including but not limited to washout or discontinuation of usual therapy, diet, or a procedure.

From the time of intervention allocation through 14 days following cessation of treatment, all AEs, SAEs, and other reportable safety events must be reported by the investigator.

Additionally, any SAE brought to the attention of an investigator at any time outside of the time period specified in the previous paragraph must be reported immediately to the Sponsor if the event is considered related to study intervention.

Investigators are not obligated to actively seek AEs or SAEs or other reportable safety events in former study participants. However, if the investigator learns of any SAE, including a death, at any time after a participant has been discharged from the study, and he/she considers the event to be reasonably related to the study intervention or study participation, the investigator must promptly notify the Sponsor.

All initial and follow-up AEs, SAEs, and other reportable safety events will be recorded and reported to the Sponsor or designee within the time frames as indicated in Table 7.

Table 7 Reporting Time Periods and Time Frames for Adverse Events and Other Reportable Safety Events

**Type of Event**	**<u>Reporting Time Period:</u>**<br>**Consent to Randomization/ Allocation**	**<u>Reporting Time Period:</u>**<br>**Randomization/ Allocation through Protocol-specified Follow-up Period**	**<u>Reporting Time Period:</u>**<br>**After the Protocol-specified Follow-up Period**	**Time Frame to Report Event and Follow-up Information to Sponsor:**
Nonserious Adverse Event (NSAE)	Report if:<br>- due to protocol-specified intervention<br>- causes exclusion<br>- participant is receiving placebo run-in or other run-in treatment	Report all	Not required	Per data entry guidelines
Serious Adverse Event (SAE)	Report if:<br>- due to protocol-specified intervention<br>- causes exclusion<br>- participant is receiving placebo run-in or other run-in treatment	Report all	Report if:<br>- drug/vaccine related.<br>(Follow ongoing to outcome)	Within 24 hours of learning of event
Pregnancy/Lactation Exposure	Report if:<br>- due to intervention<br>- causes exclusion	Report all	Previously reported – Follow to completion/termination; report outcome	Within 24 hours of learning of event
Event of Clinical Interest (require regulatory reporting)	Report if:<br>- due to intervention<br>- causes exclusion	Report<br>- Potential drug-induced liver injury (DILI)<br>- Require regulatory reporting	Not required	Within 24 hours of learning of event
Event of Clinical Interest (do not require regulatory reporting)	Report if:<br>- due to intervention<br>- causes exclusion	Report<br>- non-DILI ECIs and other events not requiring regulatory reporting	Not required	Within 5 calendar days of learning of event
Cancer	Report if:<br>- due to intervention<br>- causes exclusion	Report all	Not required	Within 5 calendar days of learning of event
Overdose	Report if:<br>- receiving placebo run-in or other run-in medication	Report all	Not required	Within 5 calendar days of learning of event

8.4.2 Method of Detecting AEs, SAEs, and Other Reportable Safety Events

Care will be taken not to introduce bias when detecting AEs and/or SAEs and other reportable safety events. Open-ended and nonleading verbal questioning of the participant is the preferred method to inquire about AE occurrence.

8.4.3 Follow-up of AE, SAE, and Other Reportable Safety Event Information

After the initial AE/SAE report, the investigator is required to proactively follow each participant at subsequent visits/contacts. All AEs, SAEs, and other reportable safety events, including pregnancy and exposure during breastfeeding, events of clinical interest (ECIs), cancer, and overdose will be followed until resolution, stabilization, until the event is otherwise explained, or the participant is lost to follow-up (as defined in Section 7.3). In addition, the investigator will make every attempt to follow all nonserious AEs that occur in allocated participants for outcome. Further information on follow-up procedures is given in Appendix 3.

8.4.4 Regulatory Reporting Requirements for SAE

Prompt notification (within 24 hours) by the investigator to the Sponsor of SAE is essential so that legal obligations and ethical responsibilities towards the safety of participants and the safety of a study intervention under clinical investigation are met.

The Sponsor has a legal responsibility to notify both the local regulatory authority and other regulatory agencies about the safety of a study intervention under clinical investigation. The Sponsor will comply with country-specific regulatory requirements and global laws and regulations relating to safety reporting to regulatory authorities, IRB/IECs, and investigators.

Investigator safety reports must be prepared for suspected unexpected serious adverse reactions (SUSARs) according to local regulatory requirements and Sponsor policy and forwarded to investigators as necessary.

An investigator who receives an investigator safety report describing an SAE or other specific safety information (eg, summary or listing of SAE) from the Sponsor will file it along with the IB and will notify the IRB/IEC, if appropriate according to local requirements.

8.4.5 Pregnancy and Exposure During Breastfeeding

Although pregnancy and infant exposure during breastfeeding are not considered adverse events, any pregnancy or infant exposure during breastfeeding in a participant (spontaneously reported to the investigator or their designee) that occurs during the study are reportable to the Sponsor.

All reported pregnancies must be followed to the completion/termination of the pregnancy. Pregnancy outcomes of spontaneous abortion, missed abortion, benign hydatidiform mole, blighted ovum, fetal death, intrauterine death, miscarriage and stillbirth must be reported as

serious events (Important Medical Events). If the pregnancy continues to term, the outcome (health of infant) must also be reported.

8.4.6 Disease-related Events and/or Disease-related Outcomes Not Qualifying as AEs or SAEs

Not applicable.

8.4.7 Events of Clinical Interest (ECIs)

Selected nonserious and SAEs are also known as ECIs and must be reported to the Sponsor.

Events of clinical interest for this study include:

1. An elevated AST or ALT lab value that is greater than or equal to 3X the upper limit of normal and an elevated total bilirubin lab value that is greater than or equal to 2X the upper limit of normal and, at the same time, an alkaline phosphatase lab value that is less than 2X the upper limit of normal, as determined by way of protocol-specified laboratory testing or unscheduled laboratory testing.*

   *Note: This section reflects the handling of nonserious ECI. ECI events meeting SAE criteria are to be reported in the same manner as SAE. The purpose of the criteria is to specify a threshold of abnormal hepatic tests that may require an additional evaluation for an underlying etiology. The study site guidance for assessment and follow-up of these criteria can be found in the Investigator Study File Binder (or equivalent).

8.5 Treatment of Overdose

In this study, an overdose is any dose higher than the protocol-specified dose as provided in Section 6.1.

Decisions regarding dose interruptions or modifications will be made by the investigator in consultation with the Sponsor's Clinical Director based on the clinical evaluation of the participant.

8.6 Pharmacokinetics

Blood samples for the determination of POS plasma concentrations are to be collected predose, as near the dose as possible, at Day 1 and at the Week 1, 2, 4, 6, 9, and 12 Visits. For participants on the IV formulation, additional samples are to be collected within 15 minutes after the end of infusion at Weeks 1, 2, 4, 6, 9, and 12. The Week 1 postdose PK sample is to be collected after a minimum of 7 days' treatment with POS IV. For participants on oral tablet or PFS formulations who are hospitalized, an additional sample is to be collected between 3 and 6 hours postdose at Weeks 2, 4, 6, 9, and 12. The actual time of blood sample collection should be documented in the participant's eCRF.

If a participant discontinues study treatment early, a plasma sample for drug concentration should be obtained between 8 and 24 hours after the last dose of study treatment. However, if this recommended timing is not feasible, a blood sample should be taken and the date and time of the previous POS dose, as well as the date and time of the blood sample, should be noted in the participant's eCRF.

8.6.1 Blood Collection for Plasma MK-5592

Sample collection, storage, and shipment instructions for plasma samples will be provided in the operations/laboratory manual.

8.7 Pharmacodynamics

Pharmacodynamic parameters will not be evaluated in this study.

8.8 Future Biomedical Research Sample Collection

Future biomedical research will not be conducted in this study.

8.9 Visit Requirements

Visit requirements are outlined in Section 1.3 (Schedule of Activities). Specific procedure-related details are provided above in Section 8.

8.9.1 Screening

All screening procedures outlined in the SoA must be performed within 14 days before enrollment into the study. Informed consent/assent must be obtained from the participant or legally acceptable representative of each participant before any study-specific procedures are performed other than those procedures conducted for the purpose of routine clinical care. Assent will be obtained from minors according to institutional practices. Before treatment assignment, potential participants must be evaluated to determine whether they fulfill the entry requirements as set forth in Sections 5.1 and 5.2. Local laboratory tests (pregnancy, hematology/chemistry, imaging, and mycology testing) will be used to evaluate eligibility criteria. Only potential participants with clinically acceptable results from all screening criteria are to be enrolled in the study.

8.9.2 Treatment Period Visits

8.9.2.1 Day 1 Visit

Day 1 procedures/assessments listed in the SoA must be performed before initiation of study therapy. Only hematology/chemistry and/or pregnancy tests are to be repeated if it has been more than 2 days or 1 day, respectively, since screening labs were performed. After confirming that all study inclusion/exclusion criteria are met, participant will be assigned a study allocation number by an IRT and POS treatment will be administered.

8.9.2.2 Treatment Period

All participants will initiate treatment with IV POS and may transition to oral POS after a minimum of 7 days on IV study treatment, as clinically indicated. Dosing selection and preparation are described in Section 4.3 and Section 6.2, respectively. Study visits in the treatment period will occur at Day 3 and Weeks 1, 2, 4, 6, 9 and 12. At these scheduled visits, procedures/assessments are to be performed according to the SoA.

8.9.2.3 End of Treatment (EOT) Visits

Participants who discontinue study therapy before 12 weeks should have an EOT Visit within 3 days after the EOT. If the EOT Visit falls within the window for a regular scheduled visit, the EOT Visit should occur instead of the scheduled visit and participants should complete all follow-up visits at 14 and 28 days after the last dose.

Every effort should be made, for participants who withdraw consent for participation or who are withdrawn from the study for any reason, to complete the EOT Visit as well as the final mortality assessment at Day 114.

8.9.3 Post-Treatment Visits

Participants will be required to return to the clinic approximately 14 days after the last dose of study treatment for the post-treatment visit. If the post-treatment visit occurs less than 14 days after the last dose of study treatment, a subsequent follow-up phone call should be made at 14 days after the last dose to determine if any AEs have occurred since the post-treatment clinic visit.

Participants will also be required to return to the clinic approximately 28 days after the last dose of study treatment for the post-treatment visit.

In addition, mortality data will be collected at Day 114 for all participants regardless of whether the participant discontinues study therapy early. If the Day 114 visit occurs after the 28-day follow-up visit, the mortality follow-up can be conducted by phone call.

9 STATISTICAL ANALYSIS PLAN

This section outlines the statistical analysis strategy and procedures for the study. Changes to analyses made after the protocol has been finalized will be documented in a supplemental SAP and referenced in the Clinical Study Report for the study. Post hoc exploratory analyses will be clearly identified in the Clinical Study Report.

9.1 Statistical Analysis Plan Summary

Key elements of the statistical analysis plan are summarized below; the comprehensive plan is provided in Sections 9.2 through 9.12.

**Study Design Overview**	A Phase 2, Open-Label, Non-Comparative Clinical Trial to Study the Safety and Efficacy of Posaconazole (POS, MK-5592) in Pediatric Participants Aged 2 to Less Than 18 Years With Invasive Aspergillosis
**Treatment Assignment**	Pediatric participants aged 2 to <18 years with IA will be allocated to receive POS (Day 1-7 IV formulation, Day 8-84 either IV, PFS or oral formulation). Participants will be enrolled into 2 age cohorts: Age Cohort 1 (2 to <12 years) or Age Cohort 2 (12 to <18 years).
**Analysis Populations**	**Safety**: All Participants as Treated (APaT).<br>**Efficacy**:<br>• Full Analysis Set (FAS) and<br>• Responder Population.<br>**Pharmacokinetics**: All treated participants who receive at least 7 days of POS IV solution and have postdose PK sampling while on POS IV therapy (PK Analysis Population).<br>Analysis populations are also defined in Section 9.5.
**Primary Endpoint(s)**	The primary safety endpoint is the proportion of participants in the APaT population who experience 1 or more treatment-related AEs during POS study treatment (IV or oral) plus 14 days of follow-up.<br>There are no primary efficacy or pharmacokinetic endpoints.
**Secondary Endpoints**	• Proportion of participants in the FAS population with a favorable global clinical response (partial or complete response) at the Week 6 (Day 42) Visit, at the Week 12 (Day 84) Visit, and at the EOT Visit (if different).<br>• Proportion of participants in the Responder Population who have a relapse of IA at any point after achieving favorable global clinical response through 28 days post-treatment.<br>• Cmin, Cmax, and Tmax, based upon sparse PK sampling (peak and trough concentration).<br>• Analysis of exposure-response (efficacy and safety) relationships.<br>• Participants' categorical perception of palatability and acceptability of the PFS formulation.
**Statistical Methods for Efficacy/Immunogenicity/ Pharmacokinetic Analyses**	Efficacy endpoints– overall proportion of participants in the FAS population with a favorable global clinical response at the Week 6, the Week 12, and the EOT Visits – will be estimated and the corresponding 95% confidence interval (CI) provided using the Clopper-Pearson method.<br>In addition, the overall proportion of participants in the Responder Population who had a relapse of IA through 28 days post-treatment will be provided along with other descriptive statistics.<br>A population PK analysis will be conducted as described in a separate Modeling Analysis Plan based on population PK models developed from prior pediatric and adult PK data for each formulation. Model-predicted individual concentration-time profiles will be used to derive Cmax, Cmin, Cavg, AUC, and Tmax. PK parameters for POS (Cmax, Cmin, Cavg, AUC, and Tmax) derived from the population PK analysis will be listed and summarized by formulation using descriptive statistics and to be summarized in a separate report.

**Statistical Methods for Safety Analyses**	The APaT population will be used for safety analyses. The percentage of participants who experience drug-related AEs during the treatment period plus the first 14 days of follow-up will be provided along with the corresponding 95% CI using the Clopper-Pearson method.
**Interim Analyses**	There is no prespecified interim analysis planned for this open-label trial. However, interim reviews of safety and efficacy data will be conducted by the external DMC in accordance with its charter.
**Multiplicity**	No multiplicity adjustment is planned.
**Sample Size and Power**	The sample size was chosen based on clinical, not statistical, considerations.

9.2 Responsibility for Analyses/In-House Blinding

The statistical analysis of the data obtained from this study will be the responsibility of the Clinical Biostatistics department of the Sponsor.

This study is being conducted as a nonrandomized, open-label study, ie, participants, investigators, and Sponsor personnel will be aware of participant treatment assignments after each participant is enrolled and treatment is assigned.

9.3 Hypotheses/Estimation

Objectives of the study are stated in Section 3. There are no hypotheses in this study.

9.4 Analysis Endpoints

Efficacy, pharmacokinetic and safety endpoints that will be evaluated are listed below.

9.4.1 Efficacy/Pharmacokinetic Endpoints

9.4.1.1 Efficacy Endpoints

There are no primary efficacy endpoints in this study. Efficacy endpoints include:

- Proportions of participants in the FAS population who have a favorable global clinical response (partial or complete response), as assessed by the investigator using the definitions in Section 8.2.1 (Table 6), at the Week 6 Visit, Week 12 Visit, and EOT Visit.

- Proportion of participants in the Responder Population who have a relapse of IA at any point after achieving favorable global clinical response through 28 days post-treatment. Relapse is defined as the re-emergence of clinical, radiographic, or other relevant abnormalities indicating IA.

9.4.1.2 Pharmacokinetic Endpoints

Observed peak and trough plasma concentrations will be measured directly from sparse sampling of plasma concentrations. Secondary PK parameters Cavg, Cmin, Cmax, AUC, and

Tmax will be estimated by population PK analysis and reported separately. An analysis of exposure-response (efficacy and safety) relationship will be conducted as part of the population PK analysis.

9.4.1.3 Tertiary/Exploratory Endpoints

The tertiary/exploratory endpoint is the proportion of participants in the APaT population who die at any point from the first dose of study treatment through Study Day 42 and through Study Day 114.

9.4.2 Safety Endpoints

The primary safety endpoint is the proportion of participants in the APaT population who experience 1 or more treatment-related AEs during POS study treatment phase (IV or oral) plus 14 days of follow-up.

9.4.3 Additional Endpoints

An additional secondary endpoint is participants' categorical perception of the palatability and acceptability of the PFS formulation (Section 8.3.5).

9.4.4 Derivations of Efficacy/Immunogenicity/Pharmacokinetics Endpoints

Based on the plasma concentration data obtained within this study, a separate population PK analysis will be performed. The prospective details of this analysis will be specified in a separate Modeling Analysis Plan.

9.5 Analysis Populations

9.5.1 Efficacy Analysis Populations

The FAS population will serve as the primary population for the analysis of global clinical response data in this study. The efficacy analysis population to be studied (possible, probable, or proven IA) is similar to the population used for the primary efficacy analyses conducted in the adult study (P069) of invasive aspergillosis. The FAS population consists of all allocated participants who:

- have possible, probable, or proven IA (based on modified 2020 EORTC/MSG definitions) as classified by the investigator,
- receive at least 1 dose of study treatment,
- have at least 1 post-allocation observation for the analysis endpoint subsequent to at least 1 dose of study treatment, and
- have baseline data for those analyses that require baseline data.

The Responder Population will serve as the population for the analysis of relapse.

- The Responder Population consists of participants from the FAS population who have achieved a favorable global clinical response at the end of study treatment.

9.5.2 Pharmacokinetic Analysis Population

The primary PK population for this study will be all treated participants who receive at least 7 days of POS IV solution treatment and have postdose POS PK sampling while on POS IV therapy.

9.5.3 Safety Analysis Populations

The APaT population will be used for the analysis of safety data and all-cause mortality in this study. This population consists of all enrolled participants who received at least 1 dose of study treatment, regardless of their IA classification.

9.6 Statistical Methods

9.6.1 Efficacy Analysis

The secondary efficacy endpoint, overall proportions of participants with a favorable global clinical response at Week 6, Week 12, and the EOT Visit, will be estimated and the corresponding 95% CI will be provided using the Clopper-Pearson method [Clopper, C. J. and Pearson, E. S. 1934]. A favorable global clinical response is defined in Section 8.2.1.

In addition, among those who had a favorable global clinical response, the overall proportion of participants who had a relapse of IA through 28 days post-treatment will be provided along with other descriptive statistics.

9.6.2 Pharmacokinetic Analysis

A population PK analysis will be conducted as described in a separate Modeling Analysis Plan based on population PK models developed from prior pediatric and adult PK data. The modeling analysis will be based on actual dose administered, which will account for differences in dosing regimen. Model-predicted individual concentration-time profiles will be used to determine Cmax, Cmin, Cavg, AUC, and Tmax. PK parameters for POS derived from the population PK analysis will be reported in a separate modeling and simulation report. Exposure/response assessments, including an exploration of the relationship between PK/PD indices with efficacy and safety time points, as available data allow, will be conducted as part of the population PK analysis and will also be reported separately.

9.6.3 Safety Analysis

Safety and tolerability will be assessed by clinical review of all relevant parameters including AEs and laboratory parameters. The primary safety analysis will summarize the safety data for participants during the treatment period (IV and oral treatment phases) plus 14 days of

follow-up. Additional safety summaries will be presented in aggregate and by age cohort. No further summary breakdowns by formulations will be performed due to small sample sizes and potential of switching participants back and forth among formulations.

In this descriptive study, the primary safety endpoint of the proportion of participants with drug-related AEs will be provided along with the corresponding 95% CI. The 95% CIs for the safety parameters will be estimated using the Clopper-Pearson method, which is a conservative exact method of providing CIs in this single-arm study.

All other safety parameters will be summarized using descriptive statistics. These include the proportion of participants with the following safety parameters: (1) at least 1 AE; (2) an SAE; (3) a serious and drug-related AE; (4) an AE leading to discontinuation; (5) specific AEs by System Organ Class; and (6) predefined limits of change in specific laboratory parameters. In addition, vital signs, 12-lead ECGs, and standard laboratory safety tests at time points specified in the SoA will be summarized as change from baseline.

The analysis strategy for the safety parameters is summarized in Table 8.

Table 8 Analysis Strategy for Safety Parameters

Safety Endpoint	95% CI	Descriptive Statistics
Any AE		X
Any SAE		X
Any Drug-Related AE	X	X
Any Serious and Drug-Related AE		X
Discontinuation due to AE		X
Specific AEs, SOCs or PDLCs		X
Change from Baseline Results (laboratory, ECGs, vital signs)		X
Abbreviations: AE=adverse event; CI=confidence interval; ECG = electrocardiogram; PDLC=Predefined Limit of Change; SAE=serious adverse event; SOC=System Organ Class; X=results will be provided.		

Missing values will be handled using the Data-As-Observed approach; that is, any missing values will be excluded from the analysis.

9.6.4 Other Analyses

Palatability and acceptability of the POS PFS formulation, measured using a 5-point facial hedonic scale depicting various degrees of tolerance, will be summarized. The proportion of participants responding in each category to each of the questions on the first and on the last days of the PFS treatment phase will be tabulated.

9.7 Interim Analyses

There is no prespecified interim analysis planned for this open-label trial. However, interim reviews of safety and efficacy data will be conducted by the external DMC in accordance with its charter.

9.8 Multiplicity

No multiplicity adjustments are planned for this study.

9.9 Sample Size and Power Calculations

9.9.1 Efficacy Analysis

The sample size was chosen based on clinical considerations, not statistical ones. However, of interest is the precision of the estimates of efficacy. The width of the 95% CI for the global clinical response rate will depend on the size of the population and the number of participants who achieve a favorable clinical response. In a study completed in 2019 (P069) in 575 adults and adolescents to study efficacy and safety of POS versus VOR for the treatment of invasive aspergillosis, the favorable global clinical response rates at Week 6 and Week 12 for the participants on POS were 45% and 42%, respectively [Maertens, J. A., et al 2021].

Table 9 summarizes the observed global clinical response rate and the corresponding 95% CI for a variety of observed response rates for a population of 15 participants (the overall evaluable population).

Table 9 Summary of Potential Observed Global Clinical Response Rates and 95% Confidence Intervals

Number of Participants in Population	Number Achieving Clinical Response	Observed Global Clinical Response	95% Confidence Interval[a]
15	9	60.0%	[32.3, 83.7]
	6	40.0%	[16.3, 67.7]
	3	20.0%	[4.3, 48.1]

[a] Confidence intervals are based on the Clopper-Pearson method [Clopper, C. J. and Pearson, E. S. 1934].

9.9.2 Safety Analysis

The primary safety analysis will summarize the safety data for participants during the treatment period (IV and oral treatment phases) plus 14 days of follow-up.

The APaT population is expected to include approximately 30 participants. The estimate of and the upper bound of the 95% CI for the underlying percentage of participants with a specific AE, given various hypothetical observed number of participants with that specific

AE within the study, are provided in Table 10. These calculations are based on the exact binomial method proposed by Clopper and Pearson.

Table 10 Estimate of Incidence of a Specific AE and Upper Limit of 95% CI Based on a Hypothetical Number of Participants with that Specific AE

N	Hypothetical Number of Participants with AE	Estimate of Incidence	95% CI Upper Limit[a]
30	0	0.0%	9.5%
	2	6.7%	22.1%
	5	16.7%	34.7%
	10	33.3%	52.8%
	15	50.0%	68.7%

Abbreviations: AE=adverse event; CI = confidence interval.
[a] Based on the 2-sided exact 95% confidence interval (CI) on a binomial proportion [Clopper, C. J. and Pearson, E. S. 1934].

9.10 Subgroup Analyses

No subgroup analyses are planned in this study.

9.11 Compliance (Medication Adherence)

The date and time of administration of each dose of POS, whether IV or oral (tablet or PFS) formulation, will be collected via eCRF to assess compliance for each participant. For the IV formulation, length of infusion will also be recorded.

For each participant who completes or who discontinues from the study or discontinues from study treatment, percent compliance will be calculated using the following formula:

$$\text{Percent Compliance} = \frac{\text{Number of Days on Therapy}}{\text{Number of Days Should be on Therapy}} \times 100.$$

The numerator, ie, ‘Number of Days on Therapy’, is defined as the total number of days on which a participant receives actual treatment (ie, at least 1 infusion, sachet [containing PFS] or tablet of POS). The denominator, ie, ‘Number of Days Should be on Therapy’, is defined as the total number of days from the date of the first dose through the date of the last dose of study treatment.

Summary statistics such as mean and range of percent compliance for POS study treatment (IV, tablet, and PFS formulations) will be provided by age cohort and overall for the APaT population.

9.12 Extent of Exposure

The extent of exposure to study treatment will be evaluated by summary statistics (N, mean, median, standard deviation) and frequency for the "Number of Days on Therapy" by age cohort and overall.

10 SUPPORTING DOCUMENTATION AND OPERATIONAL CONSIDERATIONS

10.1 Appendix 1: Regulatory, Ethical, and Study Oversight Considerations

10.1.1 Code of Conduct for Clinical Trials

Merck Sharp & Dohme LLC, Rahway, NJ, USA (MSD)

Code of Conduct for Interventional Clinical Trials

I. Introduction

A. Purpose

MSD, through its subsidiaries, conducts clinical trials worldwide to evaluate the safety and effectiveness of our products. As such, we are committed to designing, implementing, conducting, analyzing, and reporting these trials in compliance with the highest ethical and scientific standards. Protection of participants in clinical trials is the overriding concern in the design and conduct of clinical trials. In all cases, MSD clinical trials will be conducted in compliance with local and/or national regulations (including all applicable data protection regulations), and International Council for Harmonisation Good Clinical Practice (ICH-GCP), and also in accordance with the ethical principles that have their origin in the Declaration of Helsinki.

B. Scope

Highest ethical and scientific standards shall be endorsed for all clinical interventional investigations sponsored by MSD irrespective of the party (parties) employed for their execution (e.g., contract research organizations, collaborative research efforts). This Code is not intended to apply to trials that are observational in nature, or which are retrospective. Further, this Code does not apply to investigator-initiated trials, which are not under the full control of MSD.

II. Scientific Issues

A. Trial Conduct

1. Trial Design

Except for pilot or estimation trials, clinical trial protocols will be hypothesis-driven to assess safety, efficacy and/or pharmacokinetic or pharmacodynamic indices of MSD or comparator products. Alternatively, MSD may conduct outcomes research trials, trials to assess or validate various endpoint measures, or trials to determine patient preferences, etc.

The design (i.e., participant population, duration, statistical power) must be adequate to address the specific purpose of the trial. Participants must meet protocol entry criteria to be enrolled in the trial.

2. Site Selection

MSD selects investigative sites based on medical expertise, access to appropriate participants, adequacy of facilities and staff, previous performance in clinical trials, as well as budgetary considerations. Prior to trial initiation, sites are evaluated by MSD personnel (or individuals acting on behalf of MSD) to assess the ability to successfully conduct the trial.

3. Site Monitoring/Scientific Integrity

Investigative trial sites are monitored to assess compliance with the trial protocol and Good Clinical Practice (GCP). MSD reviews clinical data for accuracy, completeness, and consistency. Data are verified versus source documentation according to standard operating procedures. Per MSD policies and procedures, if fraud,

scientific/research misconduct or serious GCP-non-compliance is suspected, the issues are investigated. When necessary, the clinical site will be closed, the responsible regulatory authorities and ethics review committees notified.

B. Publication and Authorship

Regardless of trial outcome, MSD commits to publish the primary and secondary results of its registered trials of marketed products in which treatment is assigned, according to the pre-specified plans for data analysis. To the extent scientifically appropriate, MSD seeks to publish the results of other analyses it conducts that are important to patients, physicians, and payers. Some early phase or pilot trials are intended to be hypothesis-generating rather than hypothesis testing; in such cases, publication of results may not be appropriate since the trial may be underpowered and the analyses complicated by statistical issues such as multiplicity.

MSD's policy on authorship is consistent with the recommendations published by the International Committee of Medical Journal Editors (ICMJE). In summary, authorship should reflect significant contribution to the design and conduct of the trial, performance or interpretation of the analysis, and/or writing of the manuscript. All named authors must be able to defend the trial results and conclusions. MSD funding of a trial will be acknowledged in publications.

III. Participant Protection

A. Regulatory Authority and Ethics Committee Review (Institutional Review Board [IRB]/Independent Ethics Committee [IEC])

All protocols and protocol amendments will be submitted by MSD for regulatory authority acceptance/authorization prior to implementation of the trial or amendment, in compliance with local and/or national regulations.

The protocol, protocol amendment(s), informed consent form, investigator's brochure, and other relevant trial documents must be reviewed and approved by an IRB/IEC before being implemented at each site, in compliance with local and/or national regulations. Changes to the protocol that are required urgently to eliminate an immediate hazard and to protect participant safety may be enacted in anticipation of ethics committee approval. MSD will inform regulatory authorities of such new measures to protect participant safety, in compliance with local and/or national regulations.

B. Safety

The guiding principle in decision-making in clinical trials is that participant welfare is of primary importance. Potential participants will be informed of the risks and benefits of, as well as alternatives to, trial participation. At a minimum, trial designs will take into account the local standard of care.

All participation in MSD clinical trials is voluntary. Participants enter the trial only after informed consent is obtained. Participants may withdraw from an MSD trial at any time, without any influence on their access to, or receipt of, medical care that may otherwise be available to them.

C. Confidentiality

MSD is committed to safeguarding participant confidentiality, to the greatest extent possible. Unless required by law, only the investigator, Sponsor (or individuals acting on behalf of MSD), ethics committee, and/or regulatory authorities will have access to confidential medical records that might identify the participant by name.

D. Genomic Research

Genomic research will only be conducted in accordance with a protocol and informed consent authorized by an ethics committee.

IV. Financial Considerations

A. Payments to Investigators

Clinical trials are time- and labor-intensive. It is MSD's policy to compensate investigators (or the sponsoring institution) in a fair manner for the work performed in support of MSD trials. MSD does not pay incentives to enroll participants in its trials. However, when enrollment is particularly challenging, additional payments may be made to compensate for the time spent in extra recruiting efforts.

MSD does not pay for participant referrals. However, MSD may compensate referring physicians for time spent on chart review and medical evaluation to identify potentially eligible participants.

B. Clinical Research Funding

Informed consent forms will disclose that the trial is sponsored by MSD, and that the investigator or sponsoring institution is being paid or provided a grant for performing the trial. However, the local ethics committee may wish to alter the wording of the disclosure statement to be consistent with financial practices at that institution. As noted above, all publications resulting from MSD trials will indicate MSD as a source of funding.

C. Funding for Travel and Other Requests

Funding of travel by investigators and support staff (e.g., to scientific meetings, investigator meetings, etc.) will be consistent with local guidelines and practices.

V. Investigator Commitment

Investigators will be expected to review MSD's Code of Conduct as an appendix to the trial protocol, and in signing the protocol, agree to support these ethical and scientific standards.

10.1.2 Financial Disclosure

Financial Disclosure requirements are outlined in the US Food and Drug Administration Regulations, Financial Disclosure by Clinical Investigators (21 CFR Part 54). It is the Sponsor's responsibility to determine, based on these regulations, whether a request for Financial Disclosure information is required. It is the investigator's/subinvestigator's responsibility to comply with any such request.

The investigator/subinvestigator(s) agree, if requested by the Sponsor in accordance with 21 CFR Part 54, to provide his/her financial interests in and/or arrangements with the Sponsor to allow for the submission of complete and accurate certification and disclosure statements. The investigator/subinvestigator(s) further agree to provide this information on a Certification/Disclosure Form, commonly known as a financial disclosure form, provided by the Sponsor. The investigator/subinvestigator(s) also consent to the transmission of this information to the Sponsor in the United States for these purposes. This may involve the transmission of information to countries that do not have laws protecting personal data.

10.1.3 Data Protection

Participants will be assigned a unique identifier by the Sponsor. Any participant records or datasets that are transferred to the Sponsor will contain the identifier only; participant names or any information that would make the participant identifiable will not be transferred.

The participant must be informed that his/her personal study-related data will be used by the Sponsor in accordance with local data protection law. The level of disclosure must also be explained to the participant.

The participant must be informed that his/her medical records may be examined by Clinical Quality Assurance auditors or other authorized personnel appointed by the Sponsor, by appropriate IRB/IEC members, and by inspectors from regulatory authorities.

10.1.3.1 Confidentiality of Data

By signing this protocol, the investigator affirms to the Sponsor that information furnished to the investigator by the Sponsor will be maintained in confidence, and such information will be divulged to the IRB, IEC, or similar or expert committee; affiliated institution and employees, only under an appropriate understanding of confidentiality with such board or committee, affiliated institution and employees. Data generated by this study will be considered confidential by the investigator, except to the extent that it is included in a publication as provided in the Publications section of this protocol.

10.1.3.2 Confidentiality of Participant Records

By signing this protocol, the investigator agrees that the Sponsor (or Sponsor representative), IRB/IEC, or regulatory authority representatives may consult and/or copy study documents to verify worksheet/CRF data. By signing the consent form, the participant agrees to this process. If study documents will be photocopied during the process of verifying worksheet/CRF information, the participant will be identified by unique code only; full names/initials will be masked prior to transmission to the Sponsor.

By signing this protocol, the investigator agrees to treat all participant data used and disclosed in connection with this study in accordance with all applicable privacy laws, rules and regulations.

10.1.3.3 Confidentiality of IRB/IEC Information

The Sponsor is required to record the name and address of each IRB/IEC that reviews and approves this study. The Sponsor is also required to document that each IRB/IEC meets regulatory and ICH GCP requirements by requesting and maintaining records of the names and qualifications of the IRB/IEC members and to make these records available for regulatory agency review upon request by those agencies.

10.1.4 Committees Structure

10.1.4.1 Executive Oversight Committee

The EOC is comprised of members of Sponsor Senior Management. The EOC will receive and decide on any recommendations made by the external DMC regarding the study.

10.1.4.2 External Data Monitoring Committee

To supplement the routine study monitoring outlined in this protocol, an external DMC will monitor the interim data from this study. The voting members of the committee are external to the Sponsor. The members of the DMC must not be involved with the study in any other way (eg, they cannot be study investigators) and must have no competing interests that could affect their roles with respect to the study.

The DMC will make recommendations to the EOC regarding steps to ensure both participant safety and the continued ethical integrity of the study. Also, the DMC will review interim study results, consider the overall risk and benefit to study participants and recommend to the EOC whether the study should continue in accordance with the protocol.

Specific details regarding composition, responsibilities, and governance, including the roles and responsibilities of the various members and the Sponsor protocol team, meeting facilitation, the study governance structure, and requirements for and proper documentation of DMC reports, minutes, and recommendations will be described in the DMC charter that is reviewed and approved by all the DMC members.

10.1.5 Publication Policy

The results of this study may be published or presented at scientific meetings. The Sponsor will comply with the requirements for publication of study results. In accordance with standard editorial and ethical practice, the Sponsor will generally support publication of multicenter studies only in their entirety and not as individual site data. In this case, a coordinating investigator will be designated by mutual agreement.

If publication activity is not directed by the Sponsor, the investigator agrees to submit all manuscripts or abstracts to the Sponsor before submission. This allows the Sponsor to protect proprietary information and to provide comments.

Authorship will be determined by mutual agreement and in line with International Committee of Medical Journal Editors authorship requirements.

10.1.6 Compliance with Study Registration and Results Posting Requirements

Under the terms of the Food and Drug Administration Amendments Act (FDAAA) of 2007 and the European Medicines Agency (EMA) clinical trial Directive 2001/20/EC, the Sponsor of the study is solely responsible for determining whether the study and its results are subject to the requirements for submission to http://www.clinicaltrials.gov, www.clinicaltrialsregister.eu or other local registries. MSD, as Sponsor of this study, will review this protocol and submit the information necessary to fulfill these requirements. MSD entries are not limited to FDAAA or the EMA clinical trial directive mandated trials. Information posted will allow participants to identify potentially appropriate studies for their disease conditions and pursue participation by calling a central contact number for further information on appropriate study locations and study site contact information.

By signing this protocol, the investigator acknowledges that the statutory obligations under FDAAA, the EMA clinical trials directive, or other locally mandated registries are that of the Sponsor and agrees not to submit any information about this study or its results to those registries.

10.1.7 Compliance with Law, Audit, and Debarment

By signing this protocol, the investigator agrees to conduct the study in an efficient and diligent manner and in conformance with this protocol; generally accepted standards of GCP (eg, International Council for Harmonisation of Technical Requirements for Registration of Pharmaceuticals for Human Use GCP: Consolidated Guideline and other generally accepted standards of GCP); and all applicable federal, state and local laws, rules and regulations relating to the conduct of the clinical study.

The Code of Conduct, a collection of goals and considerations that govern the ethical and scientific conduct of clinical investigations sponsored by MSD, is provided in this appendix under the Code of Conduct for Clinical Trials.

The investigator agrees not to seek reimbursement from participants, their insurance providers, or from government programs for procedures included as part of the study reimbursed to the investigator by the Sponsor.

The investigator will promptly inform the Sponsor of any regulatory authority inspection conducted for this study.

The investigator agrees to provide the Sponsor with relevant information from inspection observations/findings to allow the Sponsor to assist in responding to any citations resulting from regulatory authority inspection and will provide the Sponsor with a copy of the proposed response for consultation before submission to the regulatory authority.

Persons debarred from conducting or working on clinical studies by any court or regulatory authority will not be allowed to conduct or work on this Sponsor’s studies. The investigator will immediately disclose in writing to the Sponsor if any person who is involved in conducting the study is debarred or if any proceeding for debarment is pending or, to the best of the investigator’s knowledge, threatened.

10.1.8 Data Quality Assurance

All participant data relating to the study will be recorded on printed or electronic CRF unless transmitted to the Sponsor or designee electronically (eg, laboratory data). The investigator or qualified designee is responsible for verifying that data entries are accurate and correct by physically or electronically signing the CRF.

Detailed information regarding Data Management procedures for this protocol will be provided separately.

The investigator must maintain accurate documentation (source data) that supports the information entered in the CRF.

The investigator must permit study-related monitoring, audits, IRB/IEC review, and regulatory agency inspections and provide direct access to source data documents.

Study documentation will be promptly and fully disclosed to the Sponsor by the investigator upon request and also shall be made available at the study site upon request for inspection, copying, review, and audit at reasonable times by representatives of the Sponsor or any regulatory authorities. The investigator agrees to promptly take any reasonable steps that are requested by the Sponsor or any regulatory authorities as a result of an audit or inspection to cure deficiencies in the study documentation and worksheets/CRFs.

The Sponsor or designee is responsible for the data management of this study including quality checking of the data.

Study monitors will perform ongoing source data review and verification to confirm that data entered into the CRF by authorized site personnel are accurate, complete, and verifiable from source documents; that the safety and rights of participants are being protected; and that the study is being conducted in accordance with the currently approved protocol and any other study agreements, ICH GCP, and all applicable regulatory requirements.

Records and documents, including participants; documented informed consent, pertaining to the conduct of this study must be retained by the investigator for 15 years after study completion unless local regulations or institutional policies require a longer retention period. No records may be destroyed during the retention period without the written approval of the Sponsor. No records may be transferred to another location or party without written notification to the Sponsor.

10.1.9 Source Documents

Source documents provide evidence for the existence of the participant and substantiate the integrity of the data collected. The investigator/institution should maintain adequate and accurate source documents and study records that include all pertinent observations on each of the site's participants. Source documents and data should be attributable, legible, contemporaneous, original, accurate, and complete. Changes to source data should be traceable, should not obscure the original entry, and should be explained if necessary (eg, via an audit trail). Source documents are filed at the investigator's site.

Data reported on the CRF or entered in the eCRF that are transcribed from source documents must be consistent with the source documents or the discrepancies must be explained. The investigator/institution may need to request previous medical records or transfer records, depending on the study. Also, current medical records must be available.

10.1.10 Study and Site Closure

The Sponsor or its designee may stop the study or study site participation in the study for medical, safety, regulatory, administrative, or other reasons consistent with applicable laws, regulations, and GCP.

In the event the Sponsor prematurely terminates a particular study site, the Sponsor or designee will promptly notify that study site's IRB/IEC as specified by applicable regulatory requirement(s).

10.2 Appendix 2: Clinical Laboratory Tests

- The tests detailed in Table 11 will be performed by the local or central laboratory, as applicable.
- Protocol-specific requirements for inclusion or exclusion of participants are detailed in Sections 5.1 and 5.2 of the protocol.
- Additional tests may be performed at any time during the study as determined necessary by the investigator or required by local regulations.

Table 11 Protocol-Required Safety and Other Laboratory Assessments

**Laboratory Assessments**	**Parameters**			
Hematology	Platelet count		White blood cell (WBC) count with Differential	
	Absolute neutrophil count (ANC)			
	Hemoglobin			
	Hematocrit			
Chemistry	Blood urea nitrogen (BUN)	Potassium	Aspartate aminotransferase (AST)	Total bilirubin (and direct bilirubin, if total bilirubin is elevated above the upper limit of normal)
	Albumin	Bicarbonate	Chloride	Magnesium
	Creatinine	Sodium	Alanine aminotransferase (ALT)	
	Glucose (fasting or non-fasting)	Calcium	Alkaline phosphatase	
Other Tests	• Urine or Serum β human chorionic gonadotropin (β-hCG) pregnancy test (as needed for females of childbearing potential)<br>• Samples for *Aspergillus* galactomannan enzyme immunoassay (EIA)			

Investigators must document their review of each laboratory safety report.

Table 12 Approximate Blood/Tissue Volumes Drawn/Collected by Trial Visit and by Sample Types

**Study Period**	**Screening**		**Treatment**								**Follow-Up**		**Unsch**	**Maximum Total estimated volume per participant (mL)**
**Visit Number/Title**	**1 Screening**	**2 Baseline**	**3**	**4**	**5**	**6**	**7**	**8**	**9**	**EOT**	**10**	**11**	**Unsch**	
**Scheduled Day/ Week and Window**	**Days -14 to -1**	**Day 1**	**Day 3 (±1 day)**	**Wk 1 (Day 7-9)**	**Wk 2 (Day 12-18)**	**Wk 4 (Day 25-33)**	**Wk 6 (Day 39-47)**	**Wk 9 (Day 60-68)**	**Wk 12 (Day 81-89)**	**EOT[a]**	**14 (+2) days after last dose**	**28 (+2) days after last dose**	**Unsch[b]**	
	**Approximate Blood Volume**													
Urine or Serum Pregnancy Test[c]	1	1	-	-	-	1	-	1	1	1	1	1	-	8
Hematology and Serum Chemistry[e]	4	4	4	4	4	4	4	4	4	4	4	4	4	52
Serum or plasma for *Aspergillus* Galactomannan EIA	1	1	1	1	1	1	1	1	1	1	1	1	1	13
Plasma Pharmacokinetic Assessment[d]	-	0.35	-	0.7	0.7	0.7	0.7	0.7	0.7	0.35	-	-	0.7	5.6

eCRF=electronic case report form; EIA=enzyme immunoassay; EOT=End of treatment; IV=intravenous; PK=pharmacokinetics; POS=Posaconazole; Unsch.=unscheduled; WK=Week

a. Participants who discontinue study therapy before 12 weeks should have an EOT Visit within 3 days after the EOT. If the EOT Visit falls within the window for a regularly scheduled visit, the EOT Visit should occur instead of that regularly scheduled visit.

b. Blood sample collection at unscheduled visits should only be performed as clinically appropriate.

c. Blood volume applies to serum pregnancy test

d. Samples to be collected predose at Day 1 and Weeks 1, 2, 4, 6, 9, & 12. The Week 1 postdose PK sample is to be collected after a minimum of 7 days' treatment with POS IV. For participants on IV formulation, additional samples to be collected within 15 minutes after the end of infusion at Weeks 1, 2, 4, 6, 9, & 12; for participants on either oral formulation who are hospitalized, additional samples to be collected between 3 and 6 hours postdose. If participant discontinues study treatment early, a PK sample is to be collected ≥8 and ≤24 hours after the last dose of study treatment, irrespective of formulation; however, if this timing is not feasible, a blood sample should be taken and the date and time of the previous POS dose, as well as the date and time of the blood sample, should be noted in the participant's eCRF (additional details in Section 8.6).

e. If weight is <12 kg, the blood volume for each visit should be reduced in accordance with local guidelines for blood sample volumes to be collected from pediatric participants.

10.3 Appendix 3: Adverse Events: Definitions and Procedures for Recording, Evaluating, Follow-up, and Reporting

10.3.1 Definitions of Medication Error, Misuse, and Abuse:

Medication Error

This is an unintended failure in the drug treatment process that leads to or has the potential to lead to harm to the patient.

Misuse

This refers to situations where the medicinal product is intentionally and inappropriately used not in accordance with the terms of the product information.

Abuse

This corresponds to the persistent or sporadic, intentional excessive use of a medicinal product for a perceived psychological or physiological reward or desired non-therapeutic effect.

10.3.2 Definition of AE

AE definition

- An AE is any untoward medical occurrence in a clinical study participant, temporally associated with the use of study intervention, whether or not considered related to the study intervention.

- NOTE: An AE can therefore be any unfavorable and unintended sign (including an abnormal laboratory finding), symptom, or disease (new or exacerbated) temporally associated with the use of a study intervention.

- NOTE: For purposes of AE definition, study intervention (also referred to as Sponsor's product) includes any pharmaceutical product, biological product, vaccine, diagnostic agent, or protocol specified procedure whether investigational or marketed (including placebo, active comparator product, or run-in intervention), manufactured by, licensed by, provided by, or distributed by the Sponsor for human use in this study.

Events meeting the AE definition

- Any abnormal laboratory test results (hematology, clinical chemistry, or urinalysis) or other safety assessments (eg, ECG, radiological scans, vital signs measurements), including those that worsen from baseline, considered clinically significant in the medical and scientific judgment of the investigator.

- Exacerbation of a chronic or intermittent pre-existing condition including either an increase in frequency and/or intensity of the condition.

- New conditions detected or diagnosed after study intervention administration even though it may have been present before the start of the study.

- Signs, symptoms, or the clinical sequelae of a suspected drug-drug interaction.

- Signs, symptoms, or the clinical sequelae of a suspected overdose of either study intervention or a concomitant medication.

- For all reports of overdose (whether accidental or intentional) with an associated AE, the AE term should reflect the clinical symptoms or abnormal test result. An overdose without any associated clinical symptoms or abnormal laboratory results is reported using the terminology "accidental or intentional overdose without adverse effect."

- Any new cancer or progression of existing cancer.

Events NOT meeting the AE definition

- Medical or surgical procedure (eg, endoscopy, appendectomy): the condition that leads to the procedure is the AE.

- Situations in which an untoward medical occurrence did not occur (social and/or convenience admission to a hospital).

- Anticipated day-to-day fluctuations of pre-existing disease(s) or condition(s) present or detected at the start of the study that do not worsen.

- Surgery planned prior to informed consent to treat a pre-existing condition that has not worsened.

- Refer to Section 8.4.6 for protocol-specific exceptions.

10.3.3 Definition of SAE

If an event is not an AE per definition above, then it cannot be an SAE even if serious conditions are met.

An SAE is defined as any untoward medical occurrence that, at any dose:

a. **Results in death**

b. **Is life-threatening**

  - The term "life-threatening" in the definition of "serious" refers to an event in which the participant was at risk of death at the time of the event. It does not refer to an event, which hypothetically might have caused death, if it were more severe.

c. **Requires inpatient hospitalization or prolongation of existing hospitalization**

- Hospitalization is defined as an inpatient admission, regardless of length of stay, even if the hospitalization is a precautionary measure for continued observation. (Note: Hospitalization for an elective procedure to treat a pre-existing condition that has not worsened is not an SAE. A pre-existing condition is a clinical condition that is diagnosed prior to the use of an MSD product and is documented in the participant's medical history.

d. **Results in persistent or significant disability/incapacity**

- The term disability means a substantial disruption of a person's ability to conduct normal life functions.

- This definition is not intended to include experiences of relatively minor medical significance such as uncomplicated headache, nausea, vomiting, diarrhea, influenza, and accidental trauma (eg, sprained ankle) that may interfere with or prevent everyday life functions but do not constitute a substantial disruption.

e. **Is a congenital anomaly/birth defect**

- In offspring of participant taking the product regardless of time to diagnosis.

f. **Other important medical events**

- Medical or scientific judgment should be exercised in deciding whether SAE reporting is appropriate in other situations such as important medical events that may not be immediately life-threatening or result in death or hospitalization but may jeopardize the participant or may require medical or surgical intervention to prevent 1 of the other outcomes listed in the above definition. These events should usually be considered serious.

- Examples of such events include invasive or malignant cancers, intensive treatment in an emergency room or at home for allergic bronchospasm, blood dyscrasias or convulsions that do not result in hospitalization, or development of drug dependency or drug abuse.

10.3.4 Additional Events Reported

Additional events that require reporting

In addition to the above criteria, AEs meeting either of the below criteria, although not serious per ICH definition, are reportable to the Sponsor.

- Is a cancer

- Is associated with an overdose

10.3.5 Recording AE and SAE

AE and SAE recording

- When an AE/SAE occurs, it is the responsibility of the investigator to review all documentation (eg, hospital progress notes, laboratory, and diagnostics reports) related to the event.

- The investigator will record all relevant AE/SAE information on the AE CRFs/worksheets at each examination.

- It is not acceptable for the investigator to send photocopies of the participant's medical records to the Sponsor in lieu of completion of the AE CRF page.

- There may be instances when copies of medical records for certain cases are requested by the Sponsor. In this case, all participant identifiers, with the exception of the participant number, will be blinded on the copies of the medical records before submission to the Sponsor.

- The investigator will attempt to establish a diagnosis of the event based on signs, symptoms, and/or other clinical information. In such cases, the diagnosis (not the individual signs/symptoms) will be documented as the AE/SAE.

Assessment of intensity/toxicity

- An event is defined as "serious" when it meets at least 1 of the predefined outcomes as described in the definition of an SAE, not when it is rated as severe.

- The investigator will make an assessment of intensity for each AE and SAE (and other reportable safety event) according to the NCI CTCAE, version 5.0. Any AE that changes CTCAE grade over the course of a given episode will have each change of grade recorded on the AE CRFs/worksheets.

  - Grade 1: Mild; asymptomatic or mild symptoms; clinical or diagnostic observations only; intervention not indicated.
  - Grade 2: Moderate; minimal, local or noninvasive intervention indicated; limiting age-appropriate instrumental ADL.
  - Grade 3: Severe or medically significant but not immediately life-threatening; hospitalization or prolongation of hospitalization indicated; disabling; limiting self-care ADL.
  - Grade 4: Life threatening consequences; urgent intervention indicated.
  - Grade 5: Death related to AE.

Assessment of causality

- Did the Sponsor's product cause the adverse event?

- The determination of the likelihood that the Sponsor's product caused the adverse event will be provided by an investigator who is a qualified physician. The investigator's signed/dated initials on the source document or worksheet that supports the causality noted on the AE form, ensures that a medically qualified assessment of causality was done. This initialed document must be retained for the required regulatory time frame. The criteria below are intended as reference guidelines to assist the investigator in assessing the likelihood of a relationship between the test product and the adverse event based upon the available information

- **The following components are to be used to assess the relationship between the Sponsor's product and the AE;** the greater the correlation with the components and their respective elements (in number and/or intensity), the more likely the Sponsor's product caused the adverse event:

  - **Exposure:** Is there evidence that the participant was actually exposed to the Sponsor's product such as: reliable history, acceptable compliance assessment (pill count, diary, etc.), expected pharmacologic effect, or measurement of drug/metabolite in bodily specimen?
  - **Time Course:** Did the AE follow in a reasonable temporal sequence from administration of the Sponsor's product? Is the time of onset of the AE compatible with a drug-induced effect (applies to studies with investigational medicinal product)?
  - **Likely Cause:** Is the AE not reasonably explained by another etiology such as underlying disease, other drug(s)/vaccine(s), or other host or environmental factors
  - **Dechallenge:** Was the Sponsor's product discontinued or dose/exposure/frequency reduced?
    - If yes, did the AE resolve or improve?
    - If yes, this is a positive dechallenge.
    - If no, this is a negative dechallenge.

      (Note: This criterion is not applicable if: (1) the AE resulted in death or permanent disability; (2) the AE resolved/improved despite continuation of the Sponsor's product; (3) the study is a single-dose drug study); or (4) Sponsor's product(s) is/are only used one time.)
  - **Rechallenge:** Was the participant re-exposed to the Sponsor's product in this study?
    - If yes, did the AE recur or worsen?
    - If yes, this is a positive rechallenge.
    - If no, this is a negative rechallenge.

(Note: This criterion is not applicable if: (1) the initial AE resulted in death or permanent disability, or (2) the study is a single-dose drug study); or (3) Sponsor's product(s) is/are used only one time.)

NOTE: IF A RECHALLENGE IS PLANNED FOR AN ADVERSE EVENT WHICH WAS SERIOUS AND WHICH MAY HAVE BEEN CAUSED BY THE SPONSOR'S PRODUCT, OR IF RE-EXPOSURE TO THE SPONSOR'S PRODUCT POSES ADDITIONAL POTENTIAL SIGNIFICANT RISK TO THE PARTICIPANT THEN THE RECHALLENGE MUST BE APPROVED IN ADVANCE BY THE SPONSOR CLINICAL DIRECTOR, AND IF REQUIRED, THE INSTITUTIONAL REVIEW BOARD/INDEPENDENT ETHICS COMMITTEE.

- **Consistency with Study treatment Profile:** Is the clinical/pathological presentation of the AE consistent with previous knowledge regarding the Sponsor's product or drug class pharmacology or toxicology?

- The assessment of relationship will be reported on the case report forms /worksheets by an investigator who is a qualified physician according to his/her best clinical judgment, including consideration of the above elements.

- Use the following scale of criteria as guidance (not all criteria must be present to be indicative of a Sponsor's product relationship).

  - Yes, there is a reasonable possibility of Sponsor's product relationship:
    There is evidence of exposure to the Sponsor's product. The temporal sequence of the AE onset relative to the administration of the Sponsor's product is reasonable. The AE is more likely explained by the Sponsor's product than by another cause.
  - No, there is not a reasonable possibility of Sponsor's product relationship:
    Participant did not receive the Sponsor's product OR temporal sequence of the AE onset relative to administration of the Sponsor's product is not reasonable OR the AE is more likely explained by another cause than the Sponsor’s product. (Also entered for a participant with overdose without an associated AE.)

- For each AE/SAE, the investigator must document in the medical notes that he/she has reviewed the AE/SAE and has provided an assessment of causality.

- There may be situations in which an SAE has occurred and the investigator has minimal information to include in the initial report to the Sponsor. However, it is very important that the investigator always make an assessment of causality for every event before the initial transmission of the SAE data to the Sponsor.

- The investigator may change his/her opinion of causality in light of follow-up information and send an SAE follow-up report with the updated causality assessment.

- The causality assessment is one of the criteria used when determining regulatory reporting requirements

- For studies in which multiple agents are administered as part of a combination regimen, the investigator may attribute each adverse event causality to the combination regimen or to a single agent of the combination. In general, causality attribution should be assigned to the combination regimen (ie, to all agents in the regimen). However, causality attribution may be assigned to a single agent if in the investigator's opinion, there is sufficient data to support full attribution of the adverse event to the single agent.

Follow-up of AE and SAE

- The investigator is obligated to perform or arrange for the conduct of supplemental measurements and/or evaluations as medically indicated or as requested by Sponsor to elucidate the nature and/or causality of the AE or SAE as fully as possible. This may include additional laboratory tests or investigations, histopathological examinations, or consultation with other health care professionals.

- New or updated information will be recorded in the CRF.

- The investigator will submit any updated SAE data to the Sponsor within 24 hours of receipt of the information.

10.3.6 Reporting of AEs, SAEs, and Other Reportable Safety Events to the Sponsor

AE, SAE, and other reportable safety event reporting to Sponsor via electronic data collection tool

- The primary mechanism for reporting to the Sponsor will be the electronic data collection (EDC) tool.
  - Electronic reporting procedures can be found in the EDC data entry guidelines (or equivalent).
  - If the electronic system is unavailable for more than 24 hours, then the site will use the paper AE Reporting form.
    - Reference Section 8.4.1 for reporting time requirements.

- The site will enter the SAE data into the electronic system as soon as it becomes available.

- After the study is completed at a given site, the EDC tool will be taken off-line to prevent the entry of new data or changes to existing data.

- If a site receives a report of a new SAE from a study participant or receives updated data on a previously reported SAE after the EDC tool has been taken off-line, then the site can report this information on a paper SAE form or by telephone (see next section).

- Contacts for SAE reporting can be found in the Investigator Study File Binder (or equivalent).

SAE reporting to the Sponsor via paper CRF

- If the EDC tool is not operational, facsimile transmission or secure e-mail of the SAE paper CRF is the preferred method to transmit this information to the Sponsor.

- In rare circumstances and in the absence of facsimile equipment, notification by telephone is acceptable with a copy of the SAE data collection tool sent by overnight mail or courier service.

- Initial notification via telephone does not replace the need for the investigator to complete and sign the SAE CRF pages within the designated reporting time frames.

- Contacts and instructions for SAE reporting and paper reporting procedures can be found in the Investigator Study File Binder (or equivalent).

10.4 Appendix 4: Device Events, Adverse Device Events, and Medical Device Incidents: Definitions, Collection, and Documentation

Not applicable

10.5 Appendix 5: Contraceptive Guidance

10.5.1 Definitions

Females of Childbearing Potential

A female is considered fertile following menarche and until becoming postmenopausal unless permanently sterile (see below):

If fertility is unclear (eg, amenorrhea in adolescents or athletes) and a menstrual cycle cannot be confirmed before first dose of study intervention, additional evaluation should be considered.

Females in the following categories are not considered females of childbearing potential:

- Premenarchal
- Premenopausal female with 1 of the following:
  - Documented hysterectomy
  - Documented bilateral salpingectomy
  - Documented bilateral oophorectomy

  For individuals with permanent infertility due to an alternate medical cause other than the above (eg, Mullerian agenesis, androgen insensitivity), investigator discretion should be applied to determining study entry.

  Note: Documentation can come from the site personnel's review of the participant's medical records, medical examination, or medical history interview.
- Postmenopausal female
  - A postmenopausal state is defined as no menses for 12 months without an alternative medical cause.
    - A high FSH level in the postmenopausal range may be used to confirm a postmenopausal state in females not using hormonal contraception or HRT. However, in the absence of 12 months of amenorrhea, confirmation with two FSH measurements in the postmenopausal range is required.
  - Females on HRT and whose menopausal status is in doubt will be required to use one of the nonhormonal highly effective contraception methods if they wish to continue their HRT during the study. Otherwise, they must discontinue HRT to allow confirmation of postmenopausal status before study enrollment.

10.5.2 Contraception Requirements

Contraceptives allowed during the study include[a]:

Highly Effective Contraceptive Methods That Have Low User Dependency
Failure rate of <1% per year when used consistently and correctly.

- Progestogen-only subdermal contraceptive implant[b,c]
- IUS[c,d]
- IUD
- Bilateral tubal occlusion

- Azoospermic partner (vasectomized or secondary to medical cause)
  This is a highly effective contraception method provided that the partner is the sole male sexual partner of the female of childbearing potential and the absence of sperm has been confirmed. If not, an additional highly effective method of contraception should be used. A spermatogenesis cycle is approximately 90 days.

  Note: Documentation of azoospermia can come from the site personnel's review of the participant's medical records, medical examination, or medical history interview.

Sexual Abstinence

- Sexual abstinence is considered a highly effective method only if defined as refraining from heterosexual intercourse during the entire period of risk associated with the study intervention. The reliability of sexual abstinence needs to be evaluated in relation to the duration of the study and the preferred and usual lifestyle of the participant.

Contraceptive use by males or females should be consistent with local regulations regarding the use of contraceptive methods for participants of clinical studies.

If locally required, in accordance with CTFG guidelines, acceptable contraceptive implants are limited to those which inhibit ovulation.

Male condoms must be used in addition to hormonal contraception.

IUS is a progestin-releasing IUD.

Note: The following are not acceptable methods of contraception:

- Periodic abstinence (calendar, symptothermal, post-ovulation methods), withdrawal (coitus interruptus), spermicides only, and LAM.
- Male condom with cap, diaphragm, or sponge with spermicide.
- Male and female condom should not be used together (due to risk of failure with friction).

10.6 Appendix 6: Collection and Management of Specimens for Future Biomedical Research

Not applicable

10.7 Appendix 7: Country-specific Requirements

South Korea:

- Section 5.1, Inclusion Criterion 3: CSF galactomannan testing may be performed at the investigator's discretion; however, in this case, there should be other supporting criteria to establish IA diagnosis and classification (Appendix 9).

- Section 5.2, Exclusion Criterion 13: In South Korea, an individual who has calculated creatinine clearance < 50 mL/min/1.73 m^2 (modified Schwartz formula) at screening will be excluded from participation.

Italy:

- Section 1.3, Schedule of Activities: In Italy, ECGs are to be performed at each treatment visit (Screening, Day 1, Day 3, Week 1, Week 2, Week 4, Week 6, Week 9, Week 12, EOT, and Unscheduled). Investigators have the flexibility, based on their judgment, to perform ECGs more frequently if warranted.

10.8 Appendix 8: Abbreviations

Abbreviation	Expanded Term
ADL	activities of daily living
AE	adverse event
ALT	alanine aminotransferase
APaT	all participants as treated (population)
AST	aspartate aminotransferase
AUC	area under the concentration-time curve
BID	twice daily
CAC	Clinical Adjudication Committee
Cavg	average plasma concentration
CI	confidence interval
Cmax	observed maximal concentration
Cmin	observed minimal concentration
CNS	central nervous system
CRF	Case Report Form
CRU	clinical research unit
CSF	cerebral spinal fluid
C-SSRS	Columbia-Suicide Severity Rating Scale
CT	computerized tomography
CTCAE	Common Terminology Criteria for Adverse Events
CTFG	Clinical Trial Facilitation Group
DILI	drug-induced liver injury
DMC	Data Monitoring Committee
ECG	electrocardiogram
ECI	event of clinical interest
eCRF	electronic Case Report Form
eCTA	exploratory Clinical Trial Application
EDC	electronic data collection
EEA	European Economic Area
EIA	enzyme immunoassay
EMA	European Medicines Agency
EOC	Executive Oversight Committee
EOT	end of treatment
EU	European Union
FAS	Full Analysis Set
FDA	Food and Drug Administration
FDAAA	Food and Drug Administration Amendments Act
FSH	Follicle stimulating hormone
GCP	Good Clinical Practice
HRT	hormone replacement therapy
HSCT	hematopoietic stem cell transplant
IA	invasive aspergillosis
IB	Investigator's Brochure
ICF	Informed Consent Form
ICH	International Council for Harmonisation
IEC	Independent Ethics Committee
IFI	invasive fungal infection
IND	Investigational New Drug
IRB	Institutional Review Board
IRT	Interactive Response Technology

Abbreviation	Expanded Term
ITZ	itraconazole
IUD	intrauterine device
IUS	intrauterine hormone-releasing system
MTD	maximum tolerated dose
NDA	New Drug Application
NNRTIs	non-nucleoside reverse transcriptase inhibitors
PCR	polymerase chain reaction
PFS	gastro-resistant powder and solvent for oral suspension (formulation of posaconazole)
PK	pharmacokinetic
POS	Posaconazole
QD	once daily
QP2	(Sponsor's department of quantitative pharmacology and pharmacometrics)
RNA	ribonucleic acid
SAC	Scientific Advisory Committee
SAE	serious adverse event
SAP	Statistical Analysis Plan
SoA	schedule of activities
SUSAR	suspected unexpected serious adverse reaction
Tmax	time to maximum concentration
TNF-α	tumor necrosis factor-alpha
ULN	upper limit of normal
VOR	voriconazole

10.9 Appendix 9: Criteria for Diagnosis of Invasive Aspergillosis

To determine if a potential participant meets the 2020 EORTC/MSG consensus criteria [Donnelly, J. P., et al 2020] for possible, probable, or proven invasive aspergillosis, use the criteria below.

A potential participant can be diagnosed as having…	…if they have:
PROVEN Invasive Aspergillosis	One of the required criteria
PROBABLE Invasive Aspergillosis	One host factor AND<br>One clinical criterion AND<br>One mycological criterion
POSSIBLE Invasive Aspergillosis	One host factor AND<br>One clinical criterion

**Criteria for Proven Invasive Aspergillosis**
• Tissue nucleic acid diagnosis: Amplification of fungal DNA by PCR combined with DNA sequencing when molds are seen in formalin-fixed paraffin-embedded tissue<br>OR<br>• Microscopic analysis of sterile material: Tissue histopathologic, cytopathologic, or direct microscopic examination of a needle aspiration or biopsy specimen showing hyphal forms with evidence of associated tissue damage (either microscopically or as an infiltrate or lesion by imaging)[a]<br>OR<br>• Culture of sterile material: Recovery of *Aspergillus* species by culture from a sample obtained by a sterile procedure from a normally sterile and clinically or radiologically abnormal site consistent with an infectious disease process, excluding BAL, paranasal or mastoid sinus cavity, and urine.
a: Tissue and cells submitted for histopathology or cytopathology should be stained by Grocott-Gomori methenamine silver stain or by periodic acid Schiff stain to facilitate inspection of fungal structures. Where possible, wet mounts of specimens from foci related to invasive fungal infectious disease should be stained with a fluorescent dye (eg, calcofluor or blankophor).

**Evidence for <u>Probable and Possible</u> Invasive Aspergillosis Host Factors**
• Recent history of neutropenia (<0.5 × 10^9 neutrophils/L [<500 neutrophils/mm3]) temporally related to the onset of fungal disease[a].<br>• Hematologic malignancy, ie, active malignancy, in receipt of treatment for this malignancy, and those in remission in the recent past. These patients would comprise largely acute leukemias and lymphomas.<br>• Receipt of solid organ transplant.<br>• Receipt of an allogeneic HSCT.<br>• Treatment with other recognized T-cell immune suppressants, such as calcineurin inhibitors, tumor necrosis factor-a blockers, lymphocyte specific monoclonal antibodies, immunosuppressive nucleoside analogues during the past 90 days.<br>• Prolonged use corticosteroid (excluding among patients with allergic bronchopulmonary aspergillosis) at a mean minimum dose of ≥0.3 mg/kg/day of prednisone equivalent for ≥3 weeks in the past 60 days.<br>• Treatment with recognized B-cell immunosuppressants, such as Bruton's tyrosine kinase inhibitors, eg, ibrutinib.<br>• Acute graft-versus-host disease grade III or IV involving the gut, lungs, or liver that is refractory to first-line treatment with steroids.<br>• Inherited severe immunodeficiency (such as chronic granulomatous disease, severe combined immunodeficiency, or STAT 3 deficiency).
a: Modified from the 2020 EORTC/MSG consensus criteria: any duration of neutropenia is acceptable for possible criteria in this study.

**Evidence for <u>Probable and Possible</u> Invasive Aspergillosis Clinical Criteria[a]**
• Pulmonary aspergillosis [b]<br>○ The presence of 1 of the following 4 signs on CT:<br>▪ Dense, well-circumscribed lesions(s) with or without a halo sign<br>▪ Air-crescent sign<br>▪ Cavity<br>▪ Wedge-shaped and segmental or lobar consolidation<br>• Tracheobronchitis<br>○ Tracheobronchial ulceration, nodule, pseudomembrane, plaque, or eschar seen on bronchoscopic analysis<br>• Sinonasal disease<br>○ Acute localized pain (including pain radiating to the eye)<br>○ Nasal ulcer with black eschar<br>○ Extension from the paranasal sinus across bony barriers, including into the orbit<br>• CNS infection<br>○ At least 1 of the following 2 signs:<br>▪ Focal lesions on imaging<br>▪ Meningeal enhancement on MRI or CT
a: Must be consistent with the mycological findings, if any, and must be temporally related to current episode.<br>b: Every reasonable attempt should be made to exclude an alternative etiology.

**Evidence for <u>Probable</u> Invasive Aspergillosis**<br>**Mycological Criteria**
• Direct test<br>o Pulmonary disease<br>▪ *Aspergillus*, recovered by culture from sputum, BAL, bronchial brush, or aspirate<br>▪ Microscopical detection of fungal elements in sputum, BAL, bronchial brush, or aspirate indicating a mold<br>o Tracheobronchitis<br>▪ *Aspergillus* recovered by culture of BAL or bronchial brush<br>▪ Microscopic detection of fungal elements in BAL or bronchial brush indicating a mold<br>o Sinonasal diseases<br>▪ Mold recovered by culture of sinus aspirate samples<br>▪ Microscopic detection of fungal elements in sinus aspirate samples indicating a mold<br>• Indirect tests (detection of antigen or cell-wall constituents)<br>o Galactomannan detected in plasma, serum, BAL, or CSF<br>▪ Any one of the following:<br>• Single serum or plasma $\geq$1.0<br>• BAL fluid $\geq$1.0<br>• Single serum or plasma $\geq$0.7 with BAL fluid $\geq$0.8<br>• CSF $\geq$1.0<br>o Aspergillus PCR<br>▪ Any one of the following:<br>▪ Plasma, serum, or whole blood 2 or more consecutive PCR tests positive<br>▪ BAL fluid 2 or more duplicate PCR tests positive<br>▪ At least 1 PCR test positive in plasma, serum, or whole blood and 1 PCR test positive in BAL fluid

11 REFERENCES

[Burgos, A., et al 2008]	Burgos A, Zaoutis TE, Dvorak CC, Hoffman JA, Knapp KM, Nania JJ, et al. Pediatric invasive aspergillosis: A multicenter retrospective analysis of 139 contemporary cases. Pediatrics 2008;121(5):e1286-e1294.	[00QXJD]
[Clopper, C. J. and Pearson, E. S. 1934]	Clopper CJ, Pearson ES. The use of confidence or fiducial limits illustrated in the case of the binomial. Biometrika 1934;26(4):404-13.	[03Q7PH]
[Conant, M. M., et al 2015]	Conant MM, Sha BE, Proia LA. Use of posaconazole delayed-release tablets for treatment of invasive aspergillosis. Mycoses. 2015 May;58(5):313-4.	[04C4NY]
[Donnelly, J. P., et al 2020]	Donnelly JP, Chen SC, Kauffman CA, Steinbach WJ, Baddley JW, Verweij PE, et al. Revision and update of the consensus definitions of invasive fungal disease from the European Organization for Research and Treatment of Cancer and the Mycoses Study Group Education and Research Consortium. Clin Infect Dis. 2020 Sep 15;71(6):1367-76.	[080C85]
[Douglas, A. P., et al 2021]	Douglas AP, Smibert OC, Bajel A, Halliday CL, Lavee O, McMullan B, et al. Consensus guidelines for the diagnosis and management of invasive aspergillosis, 2021. Intern Med J. 2021;51(suppl 7):143-76.	[080S5K]
[E.U. Summary of Product Characteristics 2022]	E.U. Summary of Product Characteristics: Noxafil 40 mg/mL oral suspension; Merck Sharp & Dohme B.V., Netherlands: [date available online only]. Available from: https://www.medicines.org.uk/emc/medicine/17339/SPC/Noxafil+40mg+ml+oral+suspension/#gref.	[083GPY]

[Groll, A. H., et al 2020]	Groll AH, Abdel-Azim H, Lehrnbecher T, Steinbach WJ, Paschke A, Mangin E, et al. Pharmacokinetics and safety of posaconazole intravenous solution and powder for oral suspension in children with neutropenia: an open-label, sequential dose-escalation trial. Int J Antimicrob Agents. 2020;56:106084.	[05KXVF]
[Jeong, W., et al 2016]	Jeong W, Haywood P, Shanmuganathan N, Lindsay J, Urbancic K, Ananda-Rajah MR, et al. Safety, clinical effectiveness and trough plasma concentrations of intravenous posaconazole in patients with haematological malignancies and/or undergoing allogeneic haematopoietic stem cell transplantation: off-trial experience. J Antimicrob Chemother. 2016 Dec;71(12):3540-3547.	[04RH59]
[Lehrnbecher, T., et al 2010]	Lehrnbecher T, Attarbaschi A, Duerken M, Garbino J, Gruhn B, Kontny U, et al. Posaconazole salvage treatment in paediatric patients: A multicentre survey. Eur J Clin Microbiol Infect Dis 2010;29:1043-5.	[00QXJX]
[Maertens, J. A., et al 2021]	Maertens JA, Rahav G, Lee DG, Ponce-de-Leon A, Ramirez Sanchez IC, Klimko N, et al. Posaconazole versus voriconazole for primary treatment of invasive aspergillosis: a phase 3, randomised, controlled, non-inferiority trial. Lancet. 2021 Feb 6;397:499-509.	[080TVK]
[Martin, J. M., et al 2017]	Martin JM, Macias-Parra M, Mudry P, Conte U, Yan JL, Liu P, et al. Safety, efficacy, and exposure-response of voriconazole in pediatric patients with invasive aspergillosis, invasive candidiasis or esophageal candidiasis. Pediatr Infect Dis J. 2017 Jan;36(1):e1-e13.	[04QMXQ]
[Pana, Z. D., et al 2017]	Pana ZD, Roilides E, Warris A, Groll AH, Zaoutis T. Epidemiology of invasive fungal disease in children. J Pediatric Infect Dis Soc. 2017 Sep 1;6(suppl_1):S3-S11.	[04SB7R]

[Patterson, T. F., et al 2016]	Patterson TF, Thompson GR 3rd, Denning DW, Fishman JA, Hadley S, Herbrecht R, et al. Practice guidelines for the diagnosis and management of aspergillosis: 2016 update by the infectious diseases society of America. Clin Infect Dis. 2016 Aug 15;63(4):e1-e60.	[04QMXK]
[Segal, B. H., et al 2008]	Segal BH, Herbrecht R, Stevens DA, Ostrosky-Zeichner L, Sobel J, Viscoli C, et al. Defining responses to therapy and study outcomes in clinical trials of invasive fungal diseases: Mycoses study group and European organization for research and treatment of cancer consensus criteria. Clin Infect Dis 2008;47:674-83.	[00QXH5]
[U.S. Prescribing Information 2022]	U.S. Prescribing Information: NOXAFIL (posaconazole) injection, for intravenous use; NOXAFIL (posaconazole) delayed-release tablets, for oral use; NOXAFIL (posaconazole) oral suspension; NOXAFIL powdermix (posaconazole) for delayed-release oral suspension: Jan 2022.	[083GPX]
[Walsh, T. J., et al 2007]	Walsh TJ, Raad I, Patterson TF, Chandrasekar P, Donowitz GR, Graybill R, et al. Treatment of invasive aspergillosis with posaconazole in patients who are refractory to or intolerant of conventional therapy: An externally controlled trial. Clin Infect Dis 2007;44:2-12.	[00QXJ0]
